# Lipid A Mimetics Based on Unnatural Disaccharide Scaffold as Potent TLR4 Agonists for Prospective Immunotherapeutics and Adjuvants

**DOI:** 10.1002/chem.202200547

**Published:** 2022-05-11

**Authors:** Sebastian Strobl, Karin Hofbauer, Holger Heine, Alla Zamyatina

**Affiliations:** ^1^ Department of Chemistry University of Natural Resources and Life Sciences Muthgasse 18 Vienna 1190 Austria; ^2^ Research Group Innate Immunity Research Center Borstel-Leibniz Lung Center, Airway Research Center North (ARCN), German Center for Lung Disease (DZL) Parkallee 22 Borstel 23845 Germany

**Keywords:** carbohydrates, glycosylation, lipopolysaccharide, adjuvant, modulation of the innate immune responses

## Abstract

TLR4 is a key pattern recognition receptor that can sense pathogen‐ and danger‐ associated molecular patterns to activate the downstream signaling pathways which results in the upregulation of transcription factors and expression of interferons and cytokines to mediate protective pro‐inflammatory responses involved in immune defense. Bacterial lipid A is the primary TLR4 ligand with very complex, species‐specific, and barely predictable structure‐activity relationships. Given that therapeutic targeting of TLR4 is an emerging tool for management of a variety of human diseases, the development of novel TLR4 activating biomolecules other than lipid A is of vast importance. We report on design, chemical synthesis and immunobiology of novel glycan‐based lipid A‐mimicking molecules that can activate human and murine TLR4‐mediated signaling with picomolar affinity. Exploiting crystal structure ‐ based design we have created novel disaccharide lipid A mimetics (DLAMs) where the inherently flexible β(1→6)‐linked diglucosamine backbone of lipid A is exchanged with a conformationally restrained non‐reducing βGlcN(1↔1′)βGlcN scaffold. Excellent stereoselectivity in a challenging *β*,*β*‐1,1′ glycosylation was achieved by tuning the reactivities of donor and acceptor molecules using protective group manipulation strategy. Divergent streamlined synthesis of β,β‐1,1′‐linked diglucosamine‐derived glycolipids entailing multiple long‐chain (*R*)‐3‐ acyloxyacyl residues and up two three phosphate groups was developed. Specific 3D‐molecular shape and conformational rigidity of unnatural *β*,*β*‐1,1′‐linked diglucosamine combined with carefully optimized phosphorylation and acylation pattern ensured efficient induction of the TLR4‐mediated signaling in a species‐independent manner.

## Graphical abstract

**TLR4 activating lipid A mimetics with picomolar affinity** were assembled on the basis of an unnatural synthetic β,β‐1,1′‐linked diglucosamine scaffold. Excellent stereoselectivity in a challenging β,β‐1,1′‐glycosylation was achieved by tuning the reactivities of donor and acceptor molecules using protective group manipulation strategy. Specific 3D‐molecular shape of βGlcN(1↔1′)βGlcN disaccharide was decisive for induction of pro‐inflammatory signaling by disaccharide lipid A mimetics (*ββ*‐DLAMs) based thereof. Potent immunostimulating potential was confirmed in human and murine immune cells rendering *ββ*‐DLAMs promising vaccine adjuvant candidates.

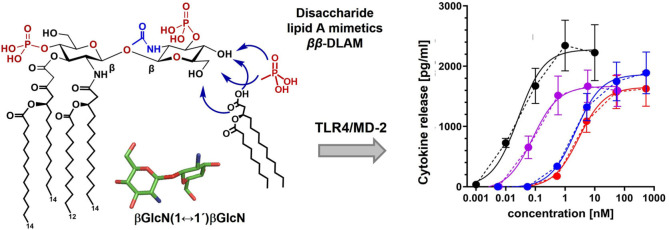

## Introduction

TLR4, an important component of the innate immune system, is a germline‐encoded pattern‐recognition receptor (PRR) responsible for the induction of defensive innate immune responses against bacterial infection. Activation of TLR4 by bacteria‐derived pathogen‐associated molecular patterns (PAMPs) such as lipopolysaccharide (LPS) as well as by danger associated molecular patterns (DAMPs) which can be generated during viral or bacterial infection or by several endogenous molecules results in induction of diverse intracellular pro‐inflammatory signaling pathways. Pathologic dysregulation of the TLR4‐mediated signaling can lead to acute or chronic inflammation and contribute to progression of a number of human diseases such as allergic asthma, atherosclerosis, arthritis, cancer and Alzheimer disease.^[1]^ Importantly, TLR4 activation also promotes antigen processing and presentation through augmented expression of costimulatory molecules on the surface of antigen‐presenting cells (APCs) which are the key parameters in initiation and regulation of the adaptive immunity.^[2]^ Thus, TLR4 agonists represent first−choice candidates for development of novel vaccine adjuvants.^[3]^ TLR4 is broadly expressed on the cell surface of immune cells such as monocytes, macrophages, and dendritic cells where it responds to various “danger” signals such as circulating PAMPs or DAMPs, as well as in tissue‐relevant cell populations, for example in the lung and bronchial epithelium, in the heart, intestinal and other tissues.

The major TLR4‐activating PAMP ‐ lipopolysaccharide (LPS, a heterogeneous glycan of ca. 10–20 kDa) found in the outer membrane of Gram‐negative bacteria contains a relatively small glycolipid fragment (ca. 2 kDa) representing the minimal entity recognised by the TLR4 complex.^[4]^ The LPS‐recognition cascade involves several soluble and membrane‐bound proteins which “deliver” LPS to the TLR4 complex where the lipid A portion of LPS can be recognized and successively bound by a secreted accessory molecule myeloid differentiation factor‐2 (MD‐2) that is physically associated with TLR4.[^4b^, ^5^] LPS‐driven dimerization of two TLR4/MD‐2/LPS complexes leads to recruitment of cytosolic adaptor molecules and the assembly of intracellular multiprotein signaling platforms.^[6]^ The latter event ultimately results in the activation of transcription factors such as NF‐κB, followed by induction of expression of interferons and cytokines.^[7]^

The LPS‐sensing receptor TLR4 was highlighted as therapeutic target already two decades ago and a number of genetically engineered LPS variants, isolated or biochemically modified lipid A derivatives as well as several synthetic compounds were suggested as candidates for pharmacological manipulation of the TLR4 system.^[8]^ However, very few TLR4 agonists were licensed for therapeutic application: MPLA (monophosphoryl lipid A–a dephosphorylated/deacylated derivative of *S. minnesota* lipid A)^[9]^ and its synthetic analogue GLA are currently in use as vaccine adjuvants along with E6020 originally developed by EISAI.^[3b]^ Especially attractive are modern developments in the syntheses of self‐adjuvanting vaccine candidates where a TLR‐ligand is covalently linked to an antigenic component.^[10]^ Therapeutic activation of the TLR4 signaling was suggested to improve the treatment outcome of disorders with immunopathological background such as asthma, allergy, arthritis, cancer or Alzheimer disease‐related pathology.^[11]^

Despite extensive and vastly successful research, the structure‐activity relationships in sensing LPS/lipid A by the TLR4/MD‐2 complex are vaguely defined which reflects a very complex nature of ligand‐protein interactions and a pronounced species‐specificity (human versus mice) in ligand recognition by the TLR4 system. This requires the development of novel structurally defined TLR4 ligands suitable for tailored modulation of the TLR4‐mediated immune responses in a well‐defined, predictable, and species‐independent manner.

## Results and Discussion

**Figure 1:**
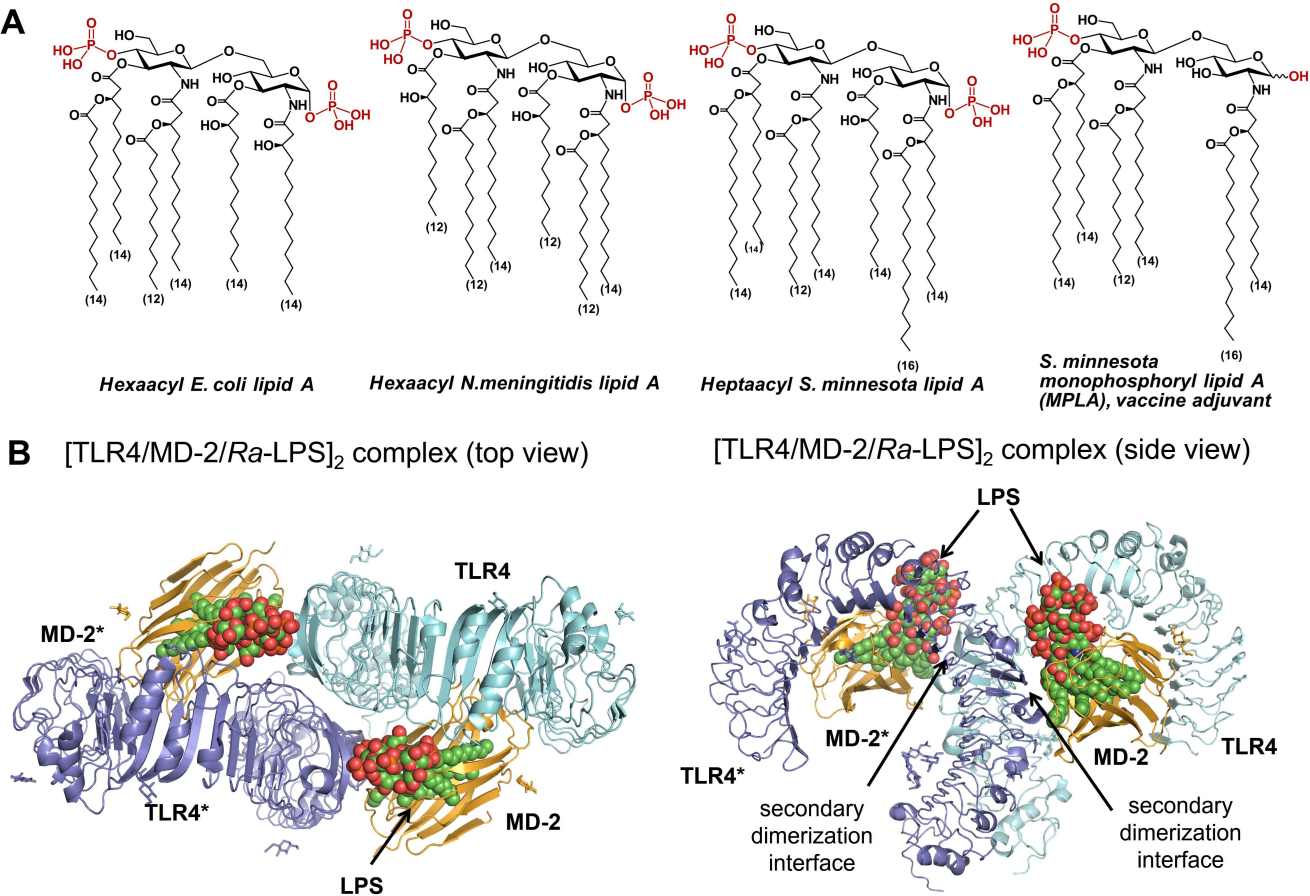
A) Chemical structure of lipid A variants from different bacterial species. B) LPS‐induced TLR4 complex dimerization. Co‐crystal structure of a hexameric [TLR4/MD‐2/*Ra*‐LPS]_2_ complex (top and side view), PDB code: 3FXI. Images were generated with ChemDoodle and PyMol.

Lipid A, the endotoxic entity of LPS, is a β(1→6)‐diglucosamine ‐ derived glycolipid that commonly contains two phosphate groups and a number of β‐hydroxylated long‐chain acyl tails. The phosphate groups are linked at positions 1‐ and/or 4′‐ and the β‐hydroxyalkanoyl and/or β‐alkanoyloxyalkanoyl chains are 2,2′‐ and/or 3,3′‐linked (Figure 1A).^[12]^ The presence of both phosphate groups of lipid A is essential for proper recognition by the TLR4/MD‐2 complex,[^4b^, ^13^] besides, number, length and distribution of lipid chains also control the TLR4 activating potential of LPS.^[8b]^ Along these lines, lipid A variants containing six long‐chain (C_12_−C_14_) (*R*)‐3‐hydroxyalkanoyl or/and (*R*)‐3‐ alkanoyloxyalkanoyl residues (such as *E. coli* and *N. meningitidis* lipid A) perform as highly endotoxic TLR4 ligands, whereas underacylated lipid A variants (penta‐ to tetra‐lipidated) are often inactive (Figure 1A).^[14]^

**Figure 2:**
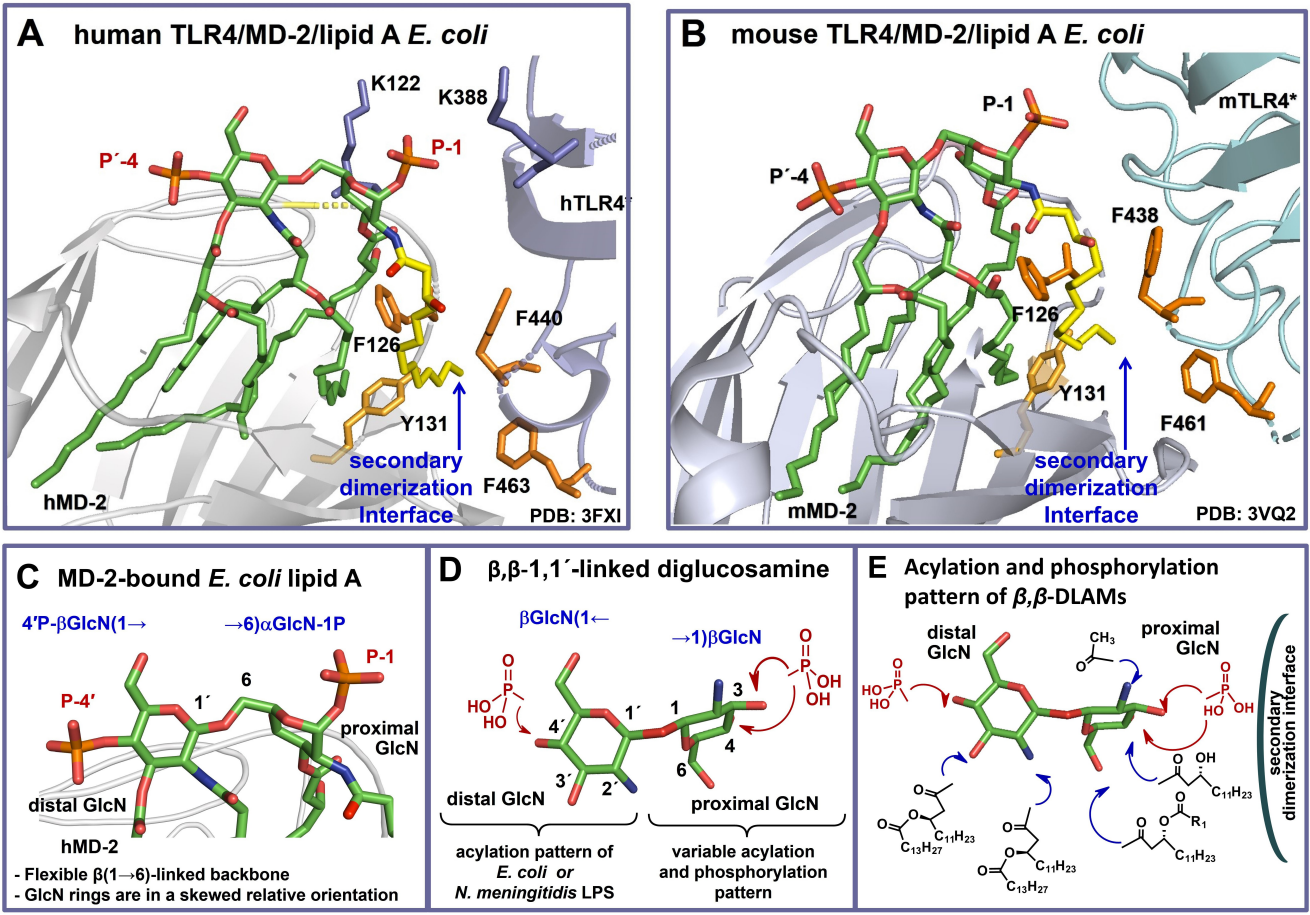
A) Co‐crystal structure of human TLR4/MD‐2/*Ra*‐LPS *E. coli* complex (PDB code: 3FXI), only lipid A portion of LPS is shown for clarity. The exterior‐positioned 2 *N*‐acyl chain (colored yellow) of hTLR4/MD‐2–bound lipid A is involved in formation of a secondary dimerization interface with the second hTLR4* complex. The secondary dimerization interface is maintained via hydrophobic attraction between 2 *N*‐acyl chain and F126/Y131 side‐chains of hMD‐2 and F440/F463 side‐chains of the second hTLR4*.^[24]^ B) Co‐crystal structure of mouse TLR4/MD‐2/*Re*‐LPS *E. coli* complex (PDB code: 3VQ2), only lipid A portion of LPS is shown for clarity. The 2 *N*‐acyl chain (colored yellow) of mTLR4/MD‐2–bound lipid A is involved in formation of a secondary dimerization interface with mTLR4*. The secondary dimerization interface is supported via hydrophobic forces involving 2 *N*‐acyl chain of lipid A and F126/Y131 of mMD‐2 together with F438/F461 of the second mTLR4*. C) Molecular shape of the β(1→6)‐linked diglucosamine backbone of TLR4/MD‐2–bound *E.coli* lipid A (PDB code: 3FXI); D) 3D‐tertiary structure of an artificial β,β‐(1↔1′)‐linked diglucosamine scaffold. The orientation of a distal pyranose ring which entails the acylation/phosphorylation pattern of *E. coli* lipid A in the target structures is set similar for both snapshots (C) and (D), such that a “skewed” topology of a proximal GlcN ring becomes evident; E) Co‐crystal structure‐based design of disaccharide lipid A mimetics (DLAMs) as potential TLR4 agonists. The acylation and phosphorylation pattern of “distal” GlcN corresponds to *E. coli* or *N. meningitidis* LPS, whereas “proximal” GlcN entails variable acylation and phosphorylation patterns. Images were generated with PyMol.

The interior of a deep hydrophobic binding pocket of MD‐2 which is lined with Leu‐ and Phe‐ residues can accommodate up to five C_12_−C_14_ lipid chains of LPS, whereas the rim of the binding pocket of human MD‐2 is crowned with multiple positively charged Arg and Lys residues which can establish salt bridges with the phosphate groups of lipid A.[^4b^, ^15^] The binding of a typical TLR4 agonist, hexaacylated LPS of *E. coli*, by the TLR4/MD‐2 complex induces receptor complex dimerization and the assembly of a hexameric [TLR4/MD‐2/LPS]_2_ complex (Figure 1B).[^4b^, ^16^] The latter occasion brings cytosolic TIR domains of TLR4 into near vicinity which directs the recruitment of adaptor proteins resulting in the assembly of a macromolecular signaling complex known as “Myddosome” which, in turn, triggers induction of the pro‐inflammatory signaling cascades.[^6a^, ^17^] The dimerization of the TLR4/MD‐2/ligand complex is enabled through hydrophobic attraction of the 2 *N*‐acyl chain attached at the proximal GlcN residue of lipid A towards hydrophobic side‐chains of the protein complex (F126/Y131 residues of MD‐2 and F440/F463 of the second TLR4*) defined as a “secondary dimerization interface” (Figure 2A ).[^5b^, ^18^]

Our design of lipid A mimetics was based on the analysis of co‐crystal structures of the TLR4‐activating lipid A variants: *E. coli Ra*‐ and *Re*‐LPS in complex with human (h)TLR4/MD‐2 complex (PDB Code: 3FXI) or mouse (m) TLR4/MD‐2 complex (PDB Code: 3VQ2), respectively (Figure 2A and B). In agreement with the PDB 3FXI and 3VQ2, five lipid chains of *Ra(Re)*‐LPS molecule are fully buried in the hydrophobic binding pocket of MD‐2, whereas the sixth 2‐*N*‐lipid chain is showing to solvent (Figure 2A). This lipid chain is involved in the formation of a secondary dimerization interface with another TLR4*/MD*‐2/LPS complex which is decisive for induction of the downstream signaling cascades.[^4b^, ^19^]

Commonly, very small variations in the length of lipid chains (e. g., C_14_ versus C_16_) or in the pattern of acyl chains distribution along the diglucosamine backbone of native lipid A are sufficient to “reprogram” the TLR4‐mediated activity and to render a TLR4 agonist inactive.[^8e^, ^12^, ^13^] This phenomenon can be rationalized by the inherent plasticity of both binding partners: the lipid A‐binding co‐receptor MD‐2 and its natural ligand lipid A. Depending on the presence/absence and the structure of the ligand, MD‐2 can unveil multiple conformational states.[^16^, ^20^] The lipid A itself is an intrinsically flexible molecule, so that the molecular shape of an easily bendable three‐bond linked carbohydrate skeleton of lipid A (βGlcN(1→6)GlcN) can be easily altered^[21]^ in a course of ligand‐protein interaction (Figure 2C).

Considering all these structural peculiarities we deemed that restricting conformational flexibility of a lipid A‐mimicking molecule would be advantageous for customized TLR4 complex dimerization and activation. As we have recently shown, TLR4 activation induced by lipid A mimetics based on a synthetic conformationally confined α,α‐1,1′‐linked disaccharide scaffold could be predictably and adjustably regulated by specific chemical modifications.^[22]^ Along these lines, we endeavored design, synthesis and biofunctional studies of conformationally confined lipid A mimetics wherein an easily rotatable glycosidic and oxymethyl linkage in the βGlc(1→6)GlcN backbone of native lipid A is exchanged for a rigid β,β‐(1↔1′) connection (Figure 2D). The skewed relative arrangement of two pyranose rings in the nonreducing β,β‐linked disacchrides[^21^, ^23^] resembles the molecular shape of the MD‐2‐bound β(1→6)‐linked diglucosamine backbone of lipid A (as in PDB: 3FXI, 3VQ1 and 3VQ2) (Figure 2C and D); at the same time, a specific orientation of hydroxyl‐ and amino groups along unnatural β,β‐(1↔1′) linked diglucosamine offers appropriate attachment sites for lipid chains and phosphate groups.

As designed, the “distal” GlcN ring reflects the configuration (β‐gluco‐) and the acylation/phosphorylation pattern of the “distal” β‐GlcN ring of *E. coli* lipid A, whereas the “proximal” GlcN ring (which is supposed to face the secondary dimerization interface) entails variable acylation and phosphorylation patterns (Figure 2E). Noteworthy, all by now synthesized lipid A mimicking compounds were based either on the native β(1→6)‐[AZ1] diglucosamine backbone or on the even more flexible skeletons where one or both GlcN residues of lipid A were exchanged for linear aglycons.[^3b^, ^3c^, ^8f^, ^24^]

For the synthesis of target biomolecules possessing non‐symmetrically distributed functional groups (Figure 2E) a convergent approach inferring first the preparation of a fully orthogonally protected β,β‐1,1′‐linked diglucosamine scaffold followed by regioselective acylation with optically active (*R*)‐3‐acyloxyacyl long‐chain fatty acids of variable chain lengths and phosphorylation was envisaged. The necessity to concurrently control the stereochemistry at two anomeric centers renders the chemical synthesis of nonreducing disaccharide a formidable challenge. Approaches to 1,1′‐glycosylation commonly suffer from either lack of stereoselectivity and concomitant formation of diastereomeric by‐products, or moderate yields. Whereas the synthesis of α,α‐1,1′‐linked and β,α‐1,1′‐linked disaccharides received adequate attention, the assembly of β,β‐1,1′−connected nonreducing disaccharides is still an abandoned topic.^[25]^ Very few reports describe the β,β‐1,1′‐glycosylation, however, these studies were majorly performed with simply protected (acetylated/benzoylated or benzylated) disaccharides.[^25b^, ^25c^]

**Scheme 1:**
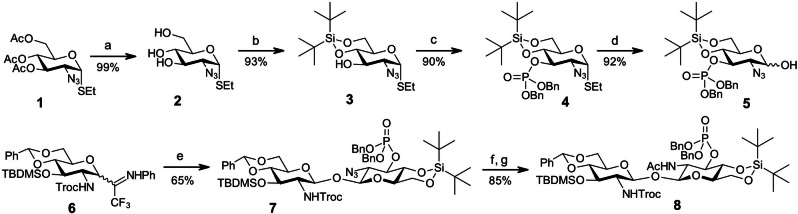
Synthesis of lactol acceptor and β,β‐1,1′‐glycosylation. Reagents and conditions: a) K_2_CO_3_, MeOH, then Dowex 50 (H^+^), b) DTBS(OTf)_2_, Py, −10 °C; c) (BnO)_2_PN(*i*Pr)_2_, 1*H*‐tetrazole, DCM, r.t., then *m*−CPBA, −78 °C; d) NBS, acetone / water (20 : 1), 0 °C; e) **5**, TMSOTf, DCM, 0 °C; f) Sn(SPh)_2_, Et_3_N, PhSH, toluene; g) Ac_2_O, Py.

Our glycosylation approach towards a fully orthogonally protected βGlcN(1↔1′)βGlcN scaffold relied on a combination of common rules for 1,2‐*trans* glycosylation on the side of glycosyl donor (participating 2‐*N*‐protecting group) and on a scrupulously elaborated structure of a matching GlcN‐derived lactol acceptor.^[26]^ Among several temporary 2‐*N*‐protecting groups tested, only 2‐azido group ensured slight conformational preference towards β−configured lactols. Although the anomeric ratio in the 2‐N_3_ protected GlcN lactols varied within α/β=1 : 1.1 to 1 : 1.3 so that a formation of diastereomeric mixture on the side of glycosyl acceptor could be expected, several electronic effects significantly improved the steric outcome of β,β‐1,1′‐glycosylation. Preliminary experiments in which variably substituted GlcN lactols were subjected to TMSOTf‐promoted glycosylation revealed that the electron‐withdrawing protecting (or functional) groups are advantageous for enhancing the β‐stereoselectivity. Also, locking the pyranose ring in a ^4^C_1_ conformation by using 4,6‐di‐*O*−cyclic protecting group proved beneficial for preponderant formation of the β−configured products.^[27]^ Therefore, lactol acceptor **5** was protected with electronically disarming 2‐azido‐ and 3‐*O*‐phosphate groups, as well as with a cyclic 4,6‐di‐*O*‐DTBS protecting group. To this end, lactol acceptor **5** was prepared in four steps from the known thioglycoside **1** (Scheme 1). Deacetylation of **1** with potassium carbonate afforded triol **2** which was regioselectively converted to 4,6‐*O*‐di‐*tert*‐butylsilylene‐protected thioglycoside **3** using di‐*tert*‐butylsilyl bis(trifluoromethanesulfonate (DTBS(OTf)_2_). The remaining 3‐OH group in **3** was phosphorylated using phosphoramidite approach to provide **4**. *N*‐Bromosuccinimide (NBS)‐mediated hydrolysis of **4** furnished lactol glycosyl acceptor **5** (α/β=1 : 1.1).

Due to significantly lower nucleophilicity, the α−configured lactol **5‐**α was resistant towards TMSOTf‐promoted glycosylation with the *N*‐phenyl trifluoroacetimidate donor **6**,^[28]^ whereas the β−configured lactol **5‐**β was instantly glycosylated to form the β,β‐linked disaccharide **7** (Scheme 1). The anomeric configuration of a newly formed β,β‐1,1′‐glycosidic linkage was confirmed by NMR (^3^ *J*_1,2_=^3^ *J*_1′,2′_=8.1 Hz). The unreacted glycosyl acceptor **5** applied in a 2‐fold excess (as an α/β= 1 : 1.1 mixture) in relation to glycosyl donor could be fully recovered using silica gel and size‐exclusion chromatography. The yield of β,β‐1,1′ disaccharide **7** was calculated based on either glycosyl donor (57 %) or on consumed glycosyl acceptor **5**‐β (65 %).

Next, the 2‐azido group in **7** was smoothly reduced with [Et_3_NH][Sn(SPh_3_)] complex^[29]^ and the liberated 2‐amino group was acetylated to provide acetamide **8**. The fully orthogonally protected β,β‐1,1′‐linked diglucosamine **8** served as versatile intermediate for the synthesis of differently functionalised disaccharide lipid A mimetics (*ββ*‐DLAMs).

**Scheme 2:**
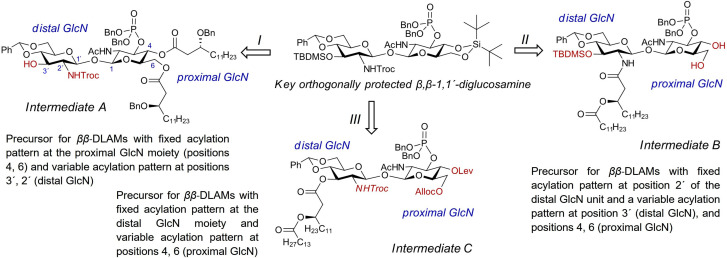
Planning divergent synthetic routes to variably acylated *ββ*‐DLAMs

When planning the synthesis of DLAMs having variable acylation pattern at the distal GlcN moiety (e. g., corresponding to different natural LPS structures), it would be most straightforward to remove the 4,6‐di‐*O*‐DTBS group first and to acylate 4‐OH [AZ2] and 6‐OH [AZ3] groups at the proximal GlcN residue next (*Intermediate A*), followed by attachment of acyl chains of variable length/structure at the 3′‐OH and 2′‐NH_2_ groups of the distal GlcN moiety (Scheme 2, Route I). For the synthesis of DLAMs having C_12_−C_14_ acyloxyacyl chain at position 2′ of the distal GlcN residue corresponding to either *E. coli* or *N. meningitidis* acylation pattern, the *intermediate B* having a pre‐installed 2′‐*N*‐acyloxyacyl chain and a possibility to differentiate the acylation and phosphorylation pattern at 3′‐OH and at 4‐OH/6‐OH of the proximal GlcN ring would be of interest (Scheme 2, Route II). If the ”distal” GlcN residue carries permanent acylation and phosphorylation pattern corresponding to *E. coli* lipid A, it would be most straightforward to remove 3′‐*O*‐TBDMS and 2′‐*N*‐Troc groups followed by introduction of 2′‐, 3′‐acyloxyacyl lipid chains first (as in the *intermediate C*) and then to manipulate acylation/phosphorylation pattern at positions 4‐ and 6‐ of the proximal GlcN residue (Scheme 2, Route III).

**Scheme 3:**
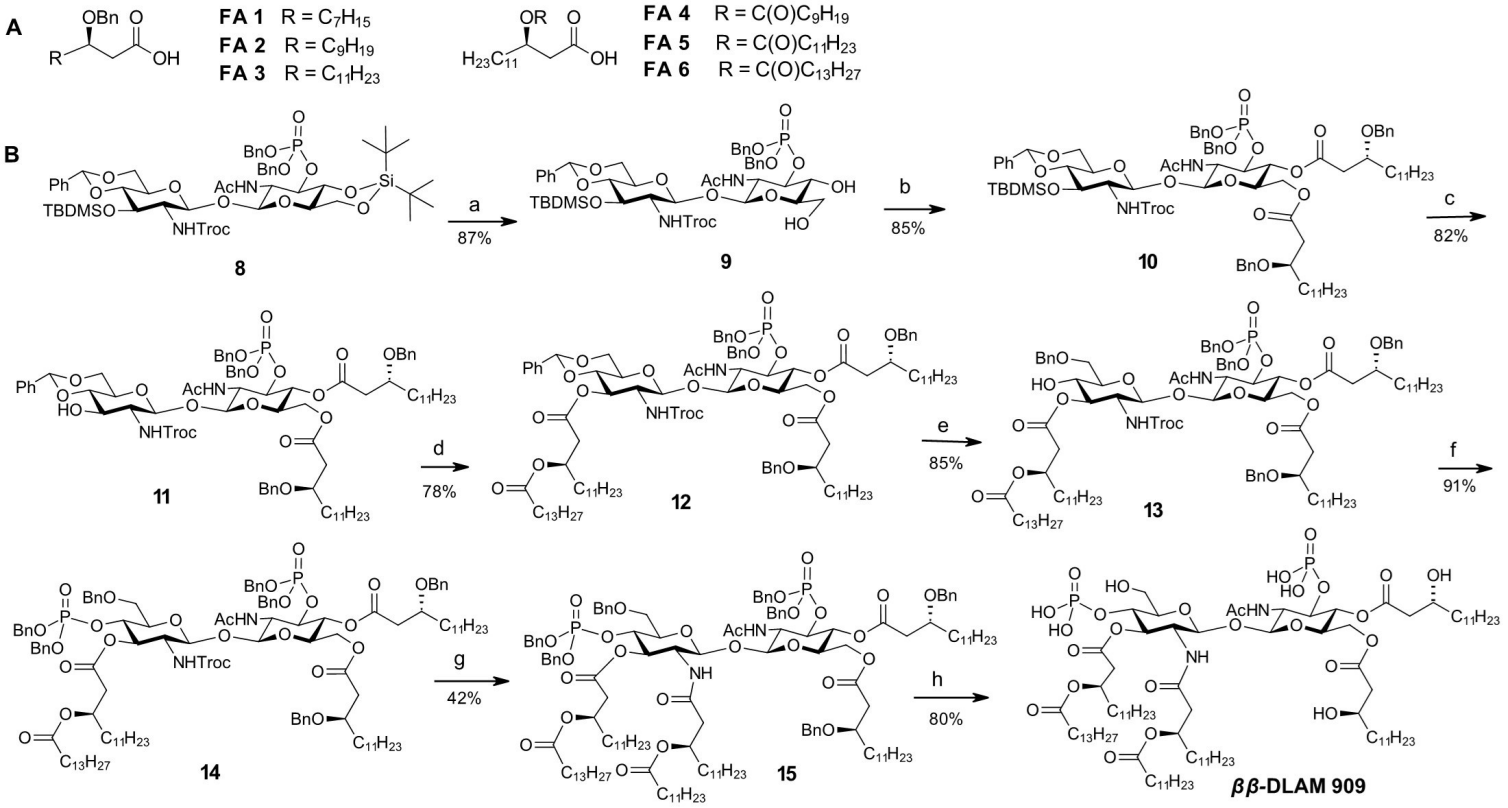
A) Chemical structure of long‐chain β‐hydroxy fatty acids. B) Synthesis of ***β*** ***β*‐DLAM909**. Reagents and conditions: a) HF ⋅ Py (70 %, 20 μL per mL THF), THF, 0 °C; b); **FA3**, DIC, DMAP, DCM; c) Et_3_N ⋅ 3HF (TREAT‐HF), DCM; d) **FA6**, DIC, DMAP, DCM; e) TfOH, Et_3_SiH, DCM, −78 °C; f) (BnO)_2_PN(*i*Pr)_2_, 1*H*‐tetrazole, DCM, r.t., then *m*−CPBA, −78° C; g) Zn, AcOH then **FA5**, HATU, DIPEA, CHCl_3_; h) Pd black, H_2_ (8.5 bar), toluene/MeOH (4 : 1).

Following the first option (Route I via *intermediate A*), the 4,6‐di‐*O*‐DTBS group in **8** was regioselectively cleaved without affecting 3′‐*O*‐TBDMS group by application of a low−concentrated solution of HF ⋅ Py to give diol **9** with nearly 90 % yield (Scheme 3).

This transformation was thoroughly optimized to reach excellent regioselectivity: multiple experiments were carried out to demonstrate that the absolute concentration (but not the number of equiv.) of fluoride reagent in reaction solution was decisive for the regioselective cleavage of 4,6‐di‐*O*‐DTBS group without affecting 3‐*O*‐TBDMS protection.

The 4,6‐diol **9** was subsequently acylated with (*R*)‐3‐ (benzyloxy)tetradecanoic acid **FA3** in the presence of 1,3‐diisopropylcarbodiimide (DIC) and DMAP to furnish diacylated **10**. Next, the 3′‐*O*‐TBDMS group was removed by treatment with a solution of Et_3_N ⋅ 3HF (TREAT‐HF) to give **11** and the liberated 3′‐hydroxyl group was acylated with (*R*)‐3‐ (tetradecanoyloxy)tetradecanoic acid **FA6** under standard conditions (DIC, DMAP) to provide tetraacylated disaccharide **12**. Thereafter, 4′,6′‐di‐*O*‐benzylidene acetal was regioselectively reductively opened using TfOH as Lewis acid and Et_3_SiH as reducing reagent in DCM to furnish 6′‐*O*‐benzylated **13** which was subsequently phosphitylated by reaction with bis(benzyloxy)(diisopropylamino)phosphine in the presence of 1*H*‐tetrazole followed by oxidation with *m*−chloroperoxybenzoic acid (*m*−CPBA) to provide **14**.

The 2′‐*N*‐Troc protecting group was removed under reductive conditions (Zn in acetic acid) which liberated 2′‐amino group for subsequent acylation with a branched long‐chain fatty acid **FA5**. The attempts to introduce (*R*)‐3‐ (dodecanoyloxy)tetradecanoyl residue using standard coupling reagents such as DIC or *N*‐(3‐dimethylaminopropyl)‐*N*′‐ethylcarbodiimide (EDC) brought about only sluggish and incomplete reaction and resulted in formation of multiple by‐products. Application of uronium‐type coupling reagent O‐(7‐azabenzotriazol‐1‐yl)‐*N,N,N′,N′*‐tetramethyluronium hexafluoro‐phosphate (HATU) in the presence of DIPEA provided substantial improvement and the hexaacylated product **15** could be isolated in pure form, although with 42 % yield only. Sluggish reaction rate and inefficient transformation were rationalised by amphiphilicity of the tetraacylated free amine intermediate and its tendency to form micelles even in organic solutions which rendered the 2′‐amino group poorly accessible for acylation reagents. Global deprotection was performed via hydrogenolysis on Pd black and the target hexaacylated bisphosphorylated ***β*** ***β*‐DLAM909** was purified by size‐exclusion chromatography on Bio‐Beads S−X1 support using toluene‐DCM‐methanol as eluent.

**Scheme 4:**
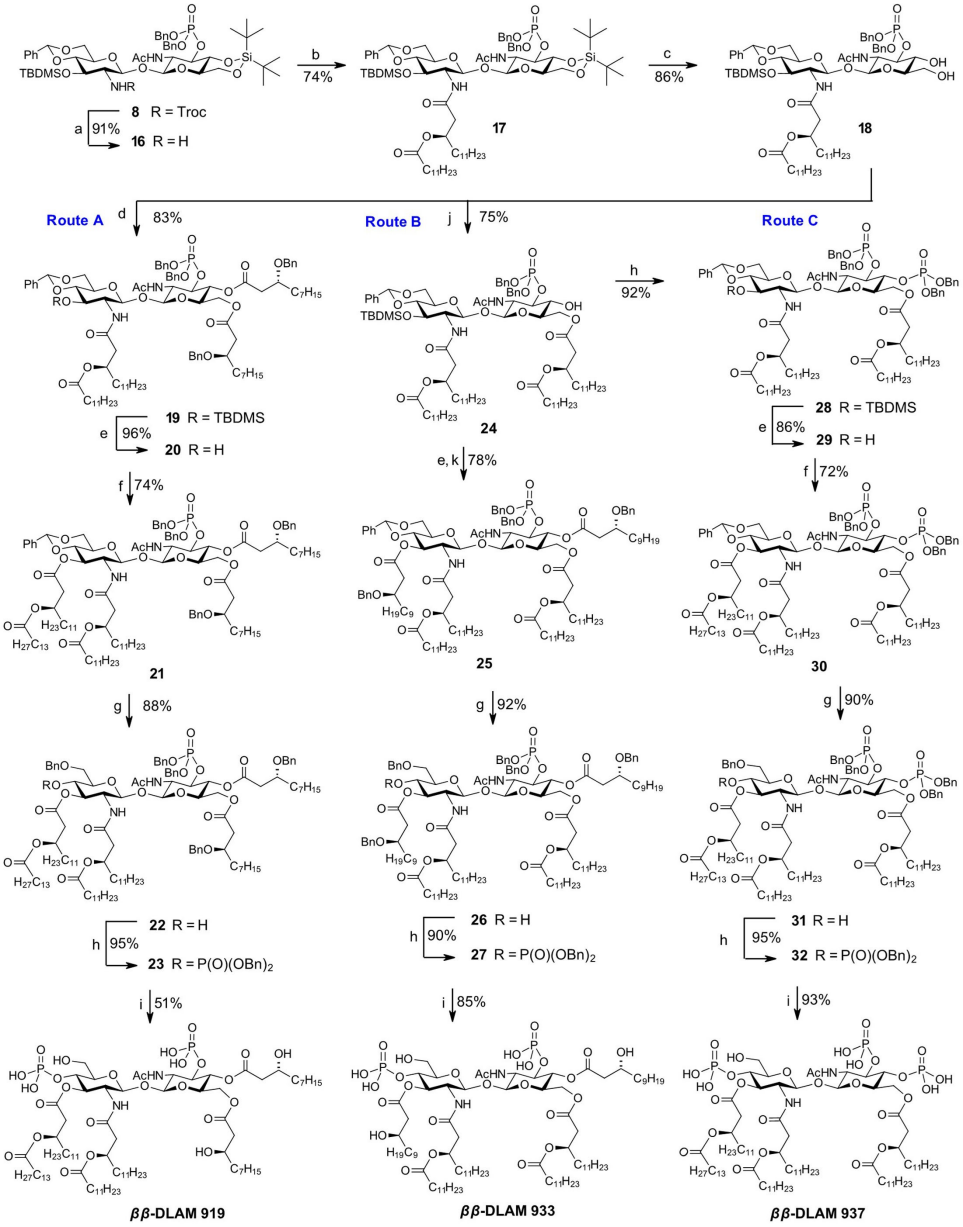
Synthesis of ***β*** ***β*‐DLAM919**, **933** and **937**. Reagents and conditions: a) Zn powder, AcOH, sonication, 0 °C; b) **FA5**, EDC ⋅ HCl, DCM; c) HF ⋅ Py, THF, 0 °C; d) **FA1**, DIC, DMAP, DCM; e) Et_3_N ⋅ 3HF (TREAT‐HF), DCM; f) **FA6**, DIC, DMAP, DCM; g) TfOH, Et_3_SiH, DCM, −78 °C; h) (BnO)_2_PN(*i*Pr)_2_, 1*H*‐tetrazole, DCM, r.t., then *m*−CPBA, −78 °C; i) Pd black, H_2_ (8.5 bar), toluene/MeOH (4 : 1); j) **FA5**, DIC, DMAP, DCM; k) **FA2**, DIC, DMAP, DCM.

Given unsatisfactory yields in the step of reduction of 2′‐*N*‐Troc group and the following *N*‐acylation which were performed at the stage of a fully assembled protected glycolipid (**14→15**), we re‐designed our synthetic route and envisaged the reductive cleavage of the 2′‐*N*‐Troc group and subsequent acylation at the very beginning of the synthesis (*Intermediate B*, Scheme 2). To this end, 2′‐*N*‐Troc group in **8** was removed using Zn in acetic acid at 0 °C and the intermediate amine **16** was carefully purified by column chromatography which allowed for substantial improvements in the next step acylation reaction compared to application of a crude product **16** (Scheme 4). Gradual addition of activated long‐chain alkanoyloxyalkanoic acid to a solution of free amine **16** also proved advantageous and resulted in formation of **17** with 74 % yield. Next, the 4,6‐di‐*O*‐DTBS protecting group in **17** was regioselectively cleaved without affecting 3‐*O*‐TBDMS protection using diluted solution of HF ⋅ Py to furnish diol **18** which was subsequently double‐acylated by (*R*)‐3‐(benzyloxy)decanoic acid **FA1** with DIC/DMAP as coupling reagent to give **19**. The 3′‐*O*‐TBDMS group was removed by treatment with Et_3_N ⋅ 3HF to give **20**, the liberated 3′‐OH group was acylated by reaction with (*R*)‐3‐ (tetradecanoyloxy)tetradecanoic acid **FA6** activated by DIC and catalytic amount of DMAP which gave hexaacylated intermediate **21**. The 4′,6′‐*O*‐benzylidene acetal in **21** was regioselectively reductively opened by reaction with TfOH/Et_3_SiH in DCM at −78 °C and the liberated 4′‐OH group in **22** was phosphorylated using phosphoramidite procedure to give **23**. Global deprotection by hydrogenolysis on Pd black afforded ***β*** ***β*‐DLAM919**, a shorter‐chain analogue of ***β*** ***β*‐DLAM909**.

To create a broader scope of lipid A mimetics for studying structure‐activity relationships in ligands recognition by the TLR4 complex we aimed to explore the biological activity of *ββ*‐DLAMs carrying acylation pattern of *N. meningitidis* lipid A (three long‐chain lipid residues attached at each GlcN ring). To this end, we performed the synthesis of hexaacylated diphosphate ***β*** ***β*‐DLAM933** starting from diol **18**. For regioselective 6‐OH‐acylation, the primary 6‐OH and secondary 4‐OH groups in **18** had to be differentiated using protective group manipulation strategy. To avoid three additional synthetic steps, we initially explored the possibility of a direct regioselective 6‐*O*‐acylation of the 4,6‐diol **18**. We reasoned that the 4‐OH group in **18** is heavily disarmed due to the presence of two electron‐withdrawing groups (2‐acetamide and 3‐*O*‐phosphate) and, therefore, will be resistant to acylation by a bulky branched long‐chain fatty acid under standard acylation conditions. Indeed, upon reaction of **18** with (*R*)‐3‐(dodecanoyloxy)tetradecanoic acid **FA5** in the presence of DIC/DMAP the primary 6‐OH group was acylated exclusively to give **24** with 75 % yield.

The cleavage of 3′‐*O*‐TBDMS protecting group generated a 3,3′‐diol which was double‐acylated with benzyl‐protected β‐hydroxy fatty acid **FA2** to give **25**. Reductive opening of benzylidene acetal in **25** followed by phosphorylation of the liberated 4′‐OH group using phosphoramidite procedure gave rise to a fully protected hexaacylated diphosphate **27**. The cleavage of all benzyl protecting groups in the molecule by hydrogenolysis over Pd black gave rise to ***β*** ***β*‐DLAM933** having *N. meningitidis*‐like acylation pattern.

**Figure 3:**
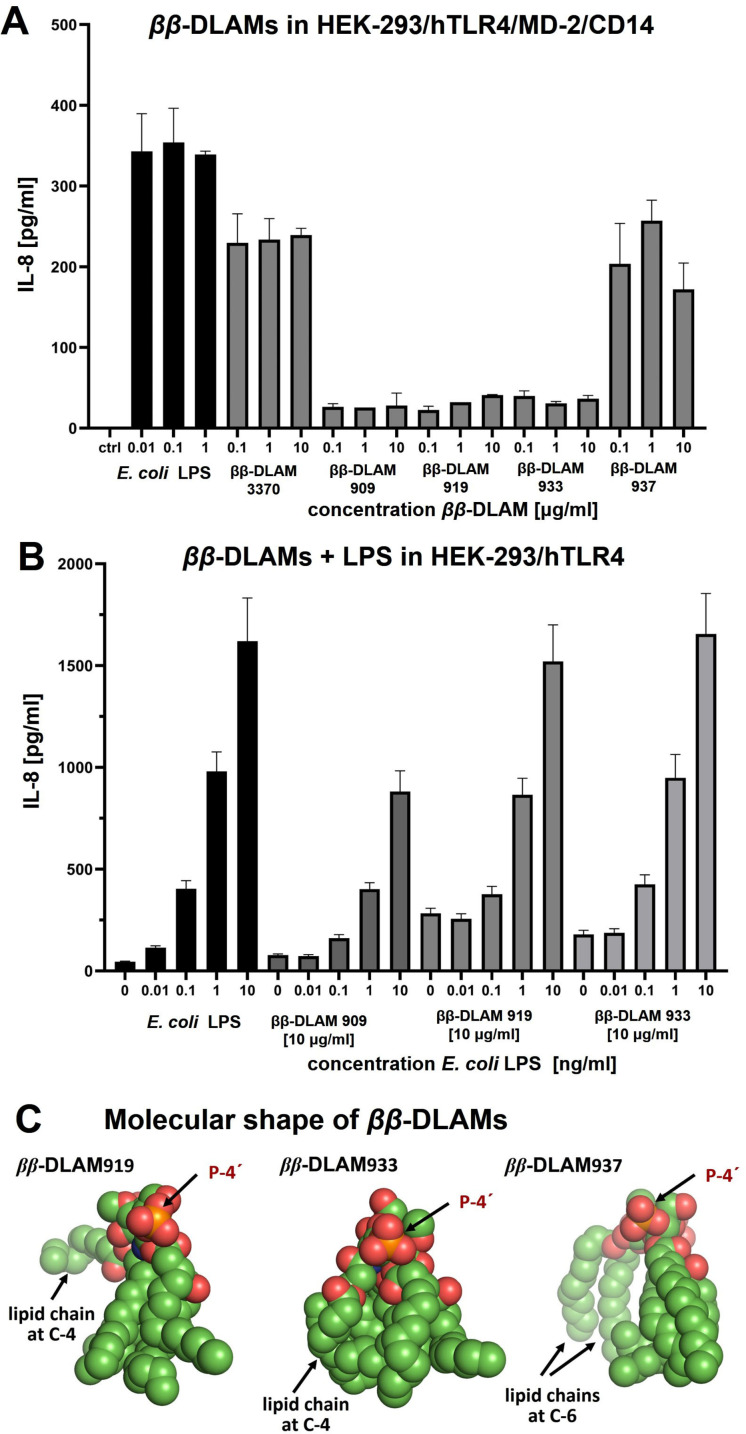
Dose‐dependent activation of TLR4‐mediated signaling induced by *ββ*‐DLAMs in TLR4/MD‐2/CD14‐transfected HEK293 cells comparted to *E. coli* LPS; (B) Inhibitory effect on *E. coli* LPS–induced release of IL‐8 by *ββ*‐DLAMs in TLR4/MD‐2/CD14–transfected HEK293 cells; (C) proposed molecular shape of variably acylated and phosphorylated *ββ*‐DLAMs. The molecules are positioned such that the “distal” GlcN with the phosphate group attached at position 4′ points to the viewer and has the same orientation in all snapshots, whereas “proximal” GlcN residue and lipid chains attached to it point away from the viewer. Images were generated with PyMol.

Immunobiological screening of three *ββ*‐DLAMs ***β*** ***β*‐DLAM909, 919** and **933** in TLR4/MD‐2/CD14‐transfected human embryonic kidney 293 cells (HEK293) revealed very low or no TLR4‐activating potential (Figure 3A). Although the acylation pattern of all three glycolipids was predicted to be highly suitable for the generation of TLR4 agonist lipid A mimetics, the 3‐*O*‐phosphate group at the proximal GlcN ring (presumable dimerization interface) was apparently misplaced and did not support appropriate ionic interactions with the second TLR4* complex. Thus, initially synthesised **ββ‐DLAM909, 919** and **933** failed to induce efficient dimerization of the TLR4/MD‐2/ligand complexes and the TLR4‐mediated signaling. We also examined whether these DLAMs could act as antagonists and inhibit the induction of pro‐inflammatory signaling induced by increasing doses of *E. coli* LPS in TLR4/MD‐2/CD14‐transfected HEK293 cells (Figure 3B). Only ***β*** ***β*‐DLAM909** could inhibit the LPS‐induced release of IL‐8 (LPS concentration 100 ng/mL to 10 μg/mL), however, at higher LPS concentration (10 μg/mL) the inhibitory effect was not significant (50 %).

Based on the analysis of co‐crystal structures of natural TLR4 agonists *E. coli Ra*‐LPS in complex with hTLR4/MD‐2 (PDB code: 3FXI) and *Re*‐LPS in complex with mTLR4/MD‐2 (PDB code: 3VQ2), the distance between 4′‐ and 1‐phosphate groups of lipid A is insignificantly longer compared to the distance between 4′‐ and 3‐phosphate groups in ***β*** ***β*‐DLAM909**/**919/933** which obviously thwarts appropriate ligand‐protein interaction and hampers dimerization. Consequently, to render the *ββ*‐DLAMs biologically active, the phosphate group must be either shifted to position 4 of the proximal GlcN moiety (3→4) or, alternatively, an additional phosphate group at position 4 could be attached. Since the binding pocket of hMD‐2 is crowned with several positively charged Lys and Arg side‐chains which can establish ionic bridges with the lipid A phosphates (this effect was demonstrated both for agonist (PDB code: 3FXI)[^4b^, ^15^] and antagonist co‐crystal structures (PDB code: 2E59 and 2Z65)),^[14]^ we hypothesised that retaining the 3‐*O*‐phosphate group at the proximal GlcN moiety could be beneficial for enhancing affinity to hMD‐2. Thus, we reasoned that attachment of an additional phosphate group in position 4 would help in establishing of a proper secondary dimerization interface with the second TLR4* by virtue of ionic interactions and would support the ligand‐induced receptor complex dimerization, whereas preserving the phosphate group at position 3 would tighten the binding of *ββ*‐DLAM to hMD‐2.

Simultaneously, the acylation pattern of the *ββ*‐DLAM molecule was adjusted: since O‐4 at the proximal GlcN residue was now occupied with a phosphate group, two lipid chains had to be attached at O‐6 in a form of (*R*)‐3‐(alkanoyloxy)alkanoyl chain as in ***β*** ***β*‐DLAM937** (Scheme 4, Route C). Such acylation pattern was deemed advantageous for high‐affinity binding to MD‐2 and further dimerization. According to our model, the shape of hydrophobic clusters formed by the long‐chain lipid tails in ***β*** ***β*‐DLAM919** and **933** could be unfavourable for binding to MD‐2 and further dimerization (Figure 3C), whereas the predicted hydrophobic profile of ***β*** ***β*‐DLAM937** with four tightly packed lipid chains attached at the distal GlcN and one alkanoyloxyalkanoyl residue linked at position 6 of the proximal GlcN should be sufficient for both high‐affinity binding to MD‐2 and presentation of 6‐*O*‐acyloxyacyl chain at the secondary dimerization interface. The latter should support the assembly of a hexameric activated receptor complex [TLR4/MD‐2/*ββ*‐DLAM]_2_.

Along these lines, the 4‐OH group in **24** was phosphorylated according to phosphoramidite procedure to give **28**, the 3‐*O*‐TBDMS group was smoothly removed using solution of TREAT‐HF in DCM followed by *O*‐acylation with (*R*)‐3‐ (tetradecanoyloxy)tetradecanoic acid **FA6** (Scheme 4, Route C) Subsequent regioselective reductive opening of benzylidene acetal to form 6‐*O*‐Bn protected **31**, followed by phosphorylation of the liberated 4′‐hydroxyl group gave rise to a fully protected hexaacylated triphosphate **32**. Global deprotection by hydrogenolysis on Pd black and purification via size‐exclusion chromatography on Bio‐Beads S−X1 provided ***β*** ***β*‐DLAM937**.

**Scheme 5:**
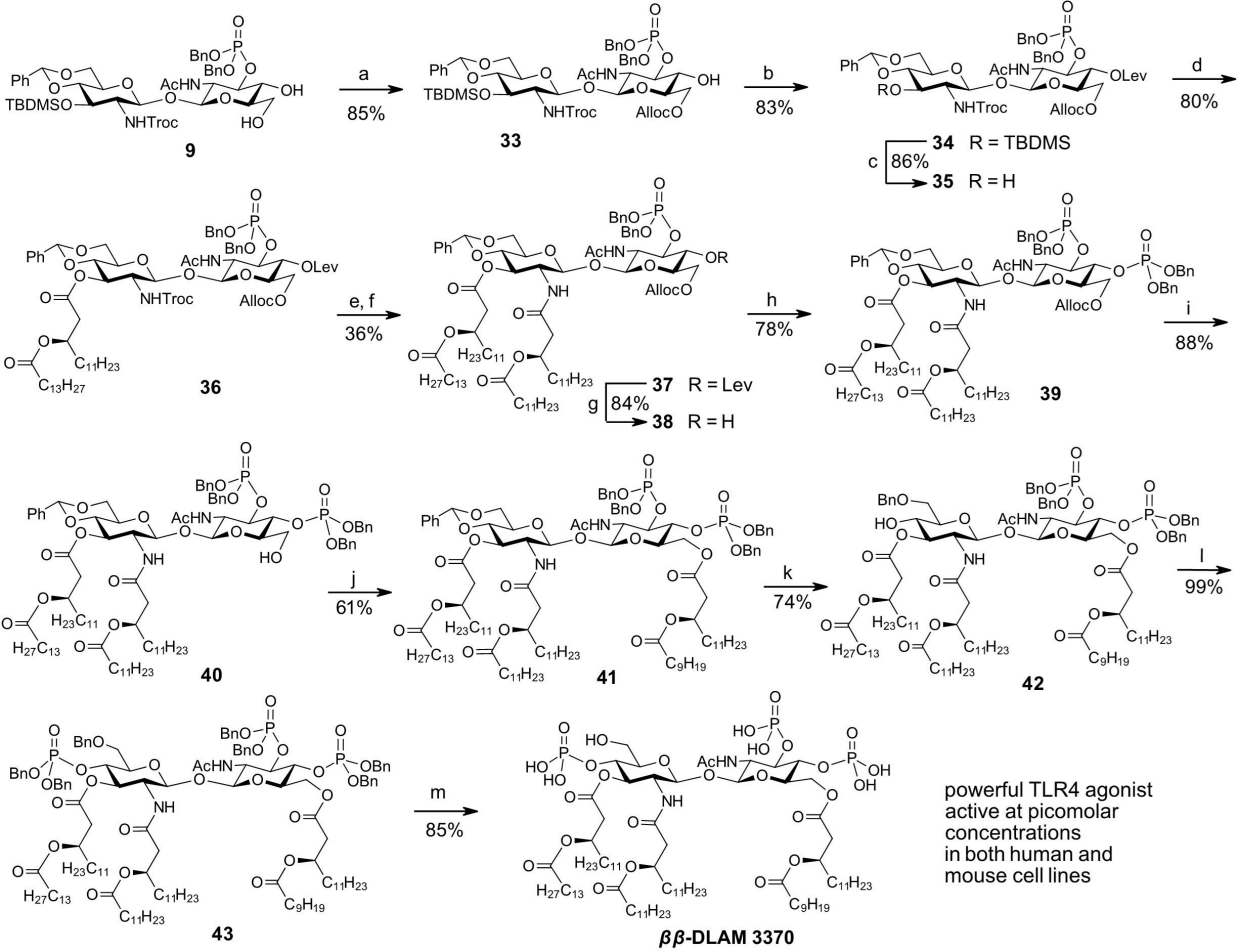
Reagents and conditions: a) AllocCl, *sym*−Collidine, DCM; b) LevOH, EDC ⋅ HCl, DMAP, DCM; c) HF−Py, THF; d) **FA6**, EDC ⋅ HCl, DMAP, DCM; e) Zn−Cu couple, AcOH; f) **FA5**, EDC ⋅ HCl, DCM; g) H_2_NNH_2_ ⋅ H_2_O, AcOH, Py; h) (BnO)_2_PN(*i*Pr)_2_, 1*H*‐tetrazole, DCM, then *tert*‐BuOOH; i) [Pd(PPh)_2_]Cl_2_, Bu_3_SnH, H_2_O, DCM; j) **FA4**, PPh_3_, DIAD, THF; k) TfOH, Et_3_SiH, DCM, −78 °C; l) (BnO)_2_PN(*i*Pr)_2_, 1*H*‐tetrazole, DCM, r.t., then *m*−CPBA, 0 °C; m) Pd black, H_2_ (8.5 bar), toluene/MeOH (2 : 1).

The length and configuration of lipid chains attached at the sugar moiety exposed at the secondary dimerization interface is known to exert profound effect on the tightness of TLR4/MD‐2/ligand complex dimerization and the ensuing downstream signaling.[^22a^, ^30^] For this reason, the synthesis of a short‐chain analogue of ***β*** ***β*‐DLAM937** starting from a key intermediate **9** was undertaken. To establish a more expedient synthetic route and to reduce the number of parallel steps towards a library of differently acylated DLAMs, we redesigned our initial synthetic approach by discriminating the 4‐ and 6‐OH groups in **9** using orthogonal protective group manipulation strategy (Scheme 2, Route III). To differentiate the 4‐ and 6‐OH groups in **9**, we applied our previously developed procedure for regioselective protection of primary 6‐OH group as allyloxycarbonate (Alloc) by a mild base‐catalysed reaction with AllocCl.^[31]^ Accordingly, the 6‐hydroxyl group in diol **9** was reacted with allyloxycarbonyl chloride in the presence of *sym*−collidine which afforded **33** as a single product in 85 % yield. The 4‐hydroxyl group in **33** was acylated with levulinic acid using EDC ⋅ HCl and DMAP which provided fully orthogonally protected disaccharide **34**. To reduce the number of parallel steps, the *distal* GlcN moiety in **34** had to be fully acylated prior to instalment of functional groups at the *proximal* GlcN unit (*Intermediate C*, Scheme 2). Thus, two branched lipid chains of different lengths (corresponding to acylation pattern of *E. coli* lipid A) were initially attached at position 2′‐, 3′‐ of the distal GlcN to produce a key intermediate **37** ready for differentiation by functional groups at the proximal GlcN moiety (Scheme 5).

To this end, desilylation of **34** furnished **35** which was subsequently acylated by reaction with (*R*)‐3‐ (tetradecanoyl‐oxy)tetradecanoic acid **FA6** using EDC ⋅ HCl/DMAP as a coupling reagent to give **36** (Scheme 5). Removal of the *N‐*Troc group in **36** was accomplished under reductive conditions with zinc−copper couple in acetic acid and the resulting amine was acylated with branched long‐chain fatty acid **FA5** using EDC ⋅ HCl as coupling reagent to give **37**. Fully protected tetraacylated intermediate **37** was treated with hydrazine hydrate to remove the levulinoyl ester from position 4 which gave **38** in 84 % yield. Next, the liberated 4‐OH group in **38** was phosphitylated with bis(benzyloxy)(diisopropylamino)phosphine in presence of 1*H*‐tetrazole, followed by oxidation with *tert‐*butyl hydroperoxide. The latter oxidant was preferred to a standard oxidative reagent *m*CPBA to avoid a concomitant epoxidation of the Alloc group. Removal of the primary Alloc protecting group from **39** was accomplished by treatment with bis(triphenylphosphine)palladium chloride and tributyltin hydride in the presence of water^[32]^ which gave **40**. In the next step, the primary 6‐OH group in **40** was acylated with branched long‐chain fatty acid **FA6** under standard conditions (EDC ⋅ HCl/DMAP) which resulted in formation of a target **41** along with elimination by‐product (due to elimination of a secondary acyl chain which gave rise to 6‐*O*‐tetradecanoyl derivative) in nearly equal amounts (1 : 1).

Elimination of a secondary acyl chain in 3‐alkanoyloxy‐alkanoyl moieties during DMAP−catalysed carbodiimide‐mediated coupling reactions has been described previously by us and others,[^8h^, ^24b^] although the formation of elimination by‐products was reported for the acylation of unreactive and/or sterically hindered secondary, but not primary OH groups. Obviously, the electron‐withdrawing effect of the 2‐NHAc and 3‐*O*‐, 4‐*O*‐ phosphate groups deactivated the primary 6‐OH group in **40** which resulted in inefficient acylation. To find a solution for a straightforward 6‐*O*‐acylation we screened numerous protocols and applied different coupling reagents (including 2‐(1*H*‐benzotriazole‐1‐yl)‐1,1,3,3‐tetramethylaminium tetrafluoroborate (TBTU) in presence of DBU,^[33]^ DIC/DMAP, etc.) but all tested conditions led to formation of a hardly separable co‐migrating elimination by‐product which lowered the isolated yield of target **41** to 30 %.

Finally, we took advantage of the Mitsunobu reaction conditions (PPh_3_, DIAD) to acylate the primary 6‐OH group in **40** which resulted in formation of **41** as a single product (Scheme 5). Next, the benzylidene acetal in **41** was regioselectively reductively opened using trifluoromethane‐sulfonic acid and triethylsilane in DCM which afforded 6‐*O*‐Bn protected **42**. The liberated 4‐OH group was phosphitylated using bis(benzyloxy)(diisopropylamino)phosphine in the presence of 1*H*‐tetrazole and subsequently in situ oxidized with *m*−CPBA to furnish **43**. Global deprotection by hydrogenolysis on Pd black followed by purification using size‐exclusion chromatography on Bio‐Beads S−X1 gave ***β*** ***β*‐DLAM3370**, a shorter‐chain analogue of ***β*** ***β*‐DLAM937**, in 85 % yield.

The capacity of ***ββ***‐**DLAM937** and ***ββ***‐**DLAM3370** to induce TLR4‐mediated activation of the NF‐κB regulated signal transduction pathway was again initially assessed in hTLR4/MD‐2/CD14 ‐ transfected HEK293 cells. The release of interleukine‐8 (IL‐8) was analysed at concentrations ranging from 100 ng/mL to 10 μg/mL and compared to responses elicited by *E. coli* LPS (Figure 3A). Both ***ββ***‐**DLAM937** [AZ4] and **3370** potently induced TLR4‐mediated release of IL‐8 and the shorter‐chain ***ββ***‐**DLAM3370** [AZ5] was somewhat more efficient compared to the longer‐chain ***ββ***‐**DLAM937**[AZ6] .

**Figure 4:**
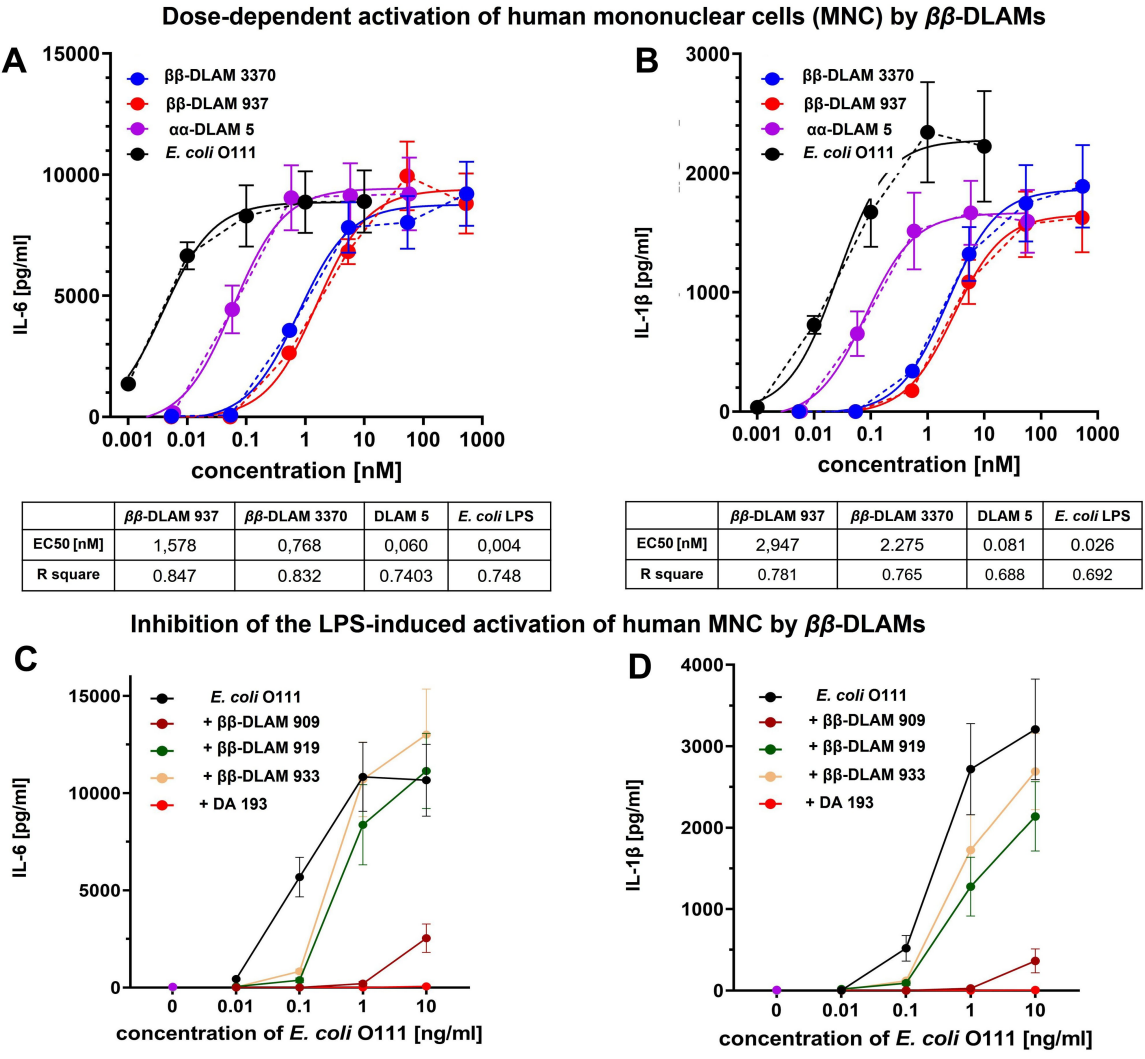
A) Dose‐dependent release of IL‐6 induced by *ββ*‐DLAMs in hMNC comparted to *E. coli* LPS; B) Dose‐dependent production of IL‐1β induced by *ββ*‐DLAMs in MNC comparted to *E. coli* LPS; C) Inhibitory effect of selected *ββ*‐DLAMs on the *E. coli* LPS–induced release of IL‐6 in MNC compared to TLR4 antagonist DA193;^[34]^ D) Inhibition of LPS‐induced expression of IL‐1β by selected *ββ*‐DLAMs in hMNC compared to TLR4 antagonist DA193.^[34]^.

Next, we evaluated the ability of *ββ*‐DLAMs to induce cytokine release in primary human immune cells by challenging human mononuclear cells (hMNC) with increasing concentrations of *ββ*‐DLAMs and compared these responses to *E. coli* LPS and a potent TLR4 agonist lipid A mimetic αα‐DLAM 5^[30]^ (Figure 4A and B). The release of IL‐6 elicited by picomolar concentrations of ***ββ***‐**DLAM3370** was two‐fold higher compared to ***ββ***‐**DLAM937** (EC_50_=0.8 nM and EC_50_=1.6 nM, respectively), although both *ββ*‐DLAMs were significantly less efficient in inducing the expression of IL‐6 than powerful TLR4 agonist αα‐DLAM 5 (EC_50=_0.06 nM) (Figure 4A). Thus, the TLR4‐activating capacity of *ββ*‐DLAMs can be readily adjusted by shortening or lengthening (for 2xCH_2_ atoms) the secondary lipid chain attached at the “proximal” GlcN unit which is supposedly exposed at the secondary dimerization interface in the hexameric [TLR4/MD‐2/*ββ*‐DLAM]_2_ complex.

Importantly, ***ββ***‐**DLAM937** and ***β*** ***β***‐**DLAM3370** induced the expression of IL‐6 in human MNC to a 100‐fold lesser extent compared to *E. coli* LPS (EC_50_=0.004 nM, calculated based on an average MW of 10 kDa) which reinforces the rationale of our crystal‐structure‐based design in an effort to achieve controlled TLR4 activation upon minimizing the LPS‐related toxic effects. Regarding the release of IL‐1β in hMNC, the relative efficacy (or maximum response) was higher for ***ββ***‐**DLAM3370** compared to ***β*** ***β***‐**DLAM937**, although the potency of response was similar (EC_50_=2.3 vs. 2.9 nmol/mL) (Figure 4 B). Both *ββ*‐DLAMs were 30‐fold less efficient in eliciting IL‐1β compared to α,α‐DLAM5^[30]^ (EC_50_=0.08 nmol/mL) and 100‐fold less potent compared to *E. coli* LPS (EC_50_=0.026 nmol/mL). Thus, both compounds under investigation induced the expression of IL‐6/IL‐1β in human primary immune cells in pico‐ to nanomolar concentration range and, as intended, were less potent (and less endotoxic) compared to *E. coli* LPS.

Since ***ββ***‐**DLAM909, 919** and **933** failed to activate TLR4‐transfected HEK293 cells and to induce the release of cytokines in MNC (data not shown), we screened these compounds for potential ability to prevent LPS‐induced TLR4 activation in human MNC. The cell culture was preincubated with the respective *ββ*‐DLAM (10 μg/mL) or with the potent TLR4 antagonist DA193[^28^, ^34^] for 1 h and then challenged with increasing concentrations of *E. coli* LPS (10 to 10.000 pg/mL). In line with the data obtained in HEK293/TLR4 cells (Figure 3B), only ***ββ***‐**DLAM909** showed weak antagonist capacity in human MNC in regard to suppression of the LPS‐induced release of IL‐6/IL‐1β (Figure 4C and D), whereas ***ββ***‐**DLAM919** and ***β*** ***β***‐**DLAM933** were inactive.

**Figure 5:**
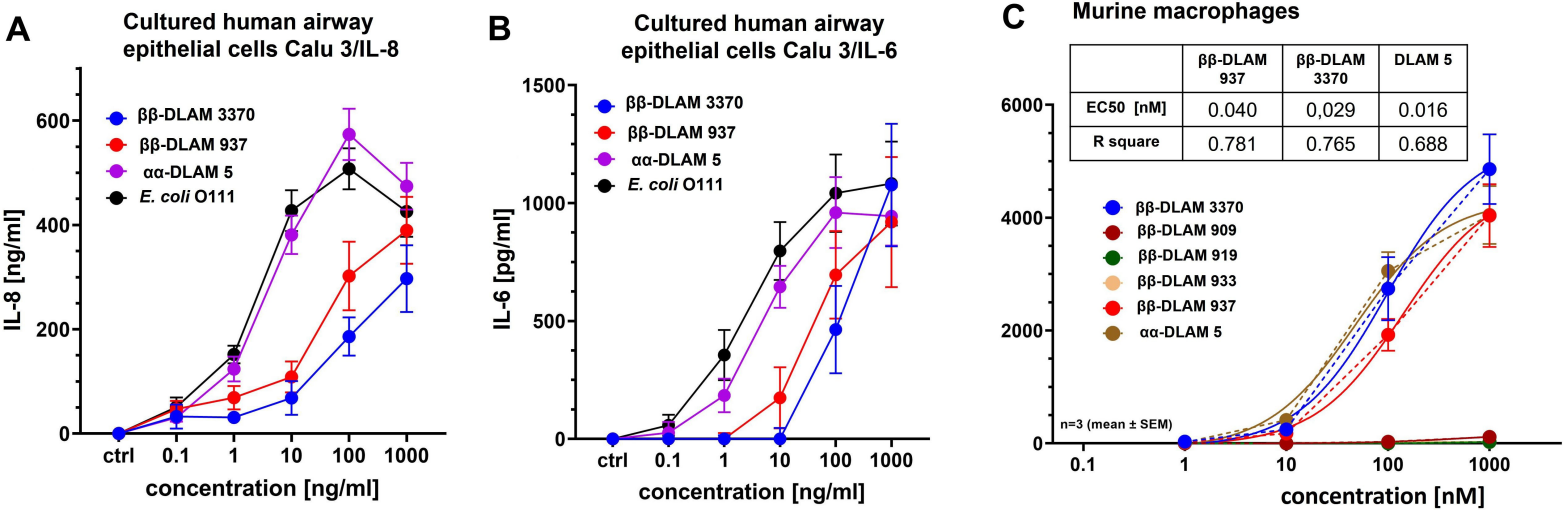
Activation of the pro‐inflammatory signaling by *ββ‐*DLAMs in cultured human airway epithelial cell line Calu‐3 and murine macrophages. A) Dose‐dependent release of IL‐8 in Calu‐3 cells; B) Dose‐dependent production of IL‐6 induced by *ββ*‐DLAMs in Calu‐3 cells; C) *ββ*‐DLAMs‐induced release of TNF‐α in bone marrow‐derived mouse wt macrophages.

We also examined the cytokine‐inducing ability of *ββ*‐DLAMs in the human bronchoepithelial cell line Calu‐3. Generally, the sensitivity of human airway epithelial cells to LPS is diminished due to insufficient expression of MD‐2^[35]^ as well as low expression levels of the membrane‐bound CD‐14.^[36]^ Accordingly, the induction of cytokine release (IL‐6/IL‐8) by *ββ*‐DLAMs in bronchoepithelial cell line required higher (nanomolar) concentrations of synthetic TLR4 ligands (Figure 5A and B). The secretion of IL‐8 in Calu‐3 cells was influenced by the length of secondary lipid chain at the proximal GlcN unit and was correspondingly higher for the longer‐chain ***ββ***‐**DLAM937** at concentrations below 100 ng/mL which related to the low‐level expression of CD‐14. Both *ββ*‐DLAMs were less potent though compared to a “reference molecule”‐a strong TLR4 agonist α,α‐DLAM 5.^[30]^

To address possible species‐specificity in the cellular activation by *ββ*‐DLAMs, we analyzed the expression of TNF‐α in bone marrow‐derived mouse wt macrophage cell line challenged with increasing concentrations of *ββ*‐DLAMs (Figure 5). The first series of *ββ*‐DLAMs **(*β*** ***β***‐**DLAM909, 919** and **933)** which could not activate hTLR4 and failed to induce cytokine release in human immune and epithelial cell lines was similarly inactive in murine macrophages (data not shown). In contrast, ***ββ***‐**DLAM937** and ***β*** ***β***‐**DLAM3370** efficiently induced the expression of TNF‐α in murine macrophages with picomolar affinity (Figure 5C). ***β*** ***β*‐DLAM3370** having shorter secondary lipid chain was more efficient in eliciting TNF‐α in murine macrophages (EC_50_=0.029 nmol/mL) compared to the longer‐chain‐lipidated ***ββ***‐**DLAM937** (EC_50_=0.04 nmol/mL), whereas the response to α,α‐DLAM5 was at least 2‐fold stronger upregulated (EC_50_=0.016 nmol/mL).

**Figure 6:**
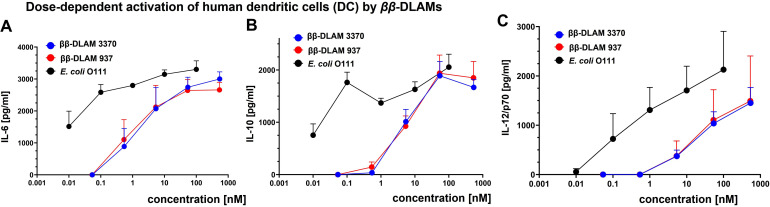
Activation of immature monocytes‐derived hDCs by *ββ*‐DLAMs. A) Induction of IL‐6 by *ββ*‐DLAMs compared to *E. coli* LPS; B) release of IL‐10 induced by *ββ*‐DLAMs compared to *E. coli* LPS; C) Production of IL‐12/p70 induced by *ββ*‐DLAMs in hDCs.

To assess the potential of *ββ*‐DLAMs to activate professional antigen‐presenting cells, dendritic cells (DCs) were challenged with increasing concentrations of DLAMs and the expression of pro‐inflammatory cytokines IL‐6 and IL‐12, as well as the production of major immunosuppressive cytokine IL‐10 was analysed (Figure 6). Accordingly, immature human monocyte‐derived DCs were stimulated with TLR4‐activating ***β*** ***β*‐DLAM937** and ***ββ***‐**DLAM3370** at concentration ranging from 0.01 mM to 1000 mM or with *E. coli* LPS as control.

Both DLAMs induced dose‐dependent release of IL‐6, IL‐10 and IL‐12 in hDCs and the responses were generally lower than those induced by *E. coli* LPS (Figure 6). The production of pro‐inflammatory cytokine IL‐6 was induced by ca. 10 nM concentration of *ββ*‐DLAMs whereas already 0.01 nM LPS was sufficient to reach the same level of induction which highlights the DLAMs as safe, less toxic TLR4 modulators compared to LPS (Figure 6A). *ββ*‐DLAMs also efficiently induced the expression of immunoregulatory cytokine IL‐10, a pleiotropic cytokine with crucial role in limiting the extent of inflammation and maintaining the homeostatic state (Figure 6B). We also demonstrate that both ***β*** ***β*‐DLAM937** and ***ββ***‐**DLAM3370** could induce the release of IL‐12, a critical factor for biasing the immune response towards a T helper 1 (Th1) cytokine profile, and that nano‐molar doses of *ββ*‐DLAMs were sufficient for this activity (Figure 6C).

## Conclusion

We developed a divergent synthetic strategy towards a series of lipid A mimicking biomolecules derived from an artificial β,β‐1,1′‐linked diglucosamine scaffold and prepared five complex glycolipids having variable acylation and phosphorylation patterns (disaccharide lipid A mimetics, *ββ*‐DLAMs). Excellent stereoselectivity in a demanding β,β‐1,1′‐glycosylation which was achieved through application of torsional‐ and electronically‐disarming orthogonal protecting groups allowed for high‐yielding, reproducible and upscalable (up to 1 g of glycosyl donor) glycosylation reaction. Compared to native TLR4 agonist lipid A which entails a readily bendable three‐bond linked diglucosamine backbone [βGlcN(1→6)GlcN], the *ββ*‐DLAM molecules are built on the basis of a conformationally constrained two‐bond linked diglucosamine scaffold [βGlcN(1↔1)βGlcN] which reveals specific tertiary structure with two pyranose rings in a staggered arrangement. The particular 3D‐molecular shape of DLAMs based thereof was decisive for efficient crosslinking of two TLR4/MD‐2/DLAM complexes and resulting induction of the intracellular signaling. As judged by production of cytokines induced by pico‐ to nanomolar concentrations of *ββ*‐DLAMs in human and murine immune cells, these synthetic glycolipids comprise a new class of unprecedentedly efficient TLR4‐dependent modulators of innate immune responses. The induction of somewhat lower levels of pro‐inflammatory cytokines TNF‐α, IL‐6, IL‐8 and IL‐1β by *ββ*‐DLAMs compared to LPS highlights *ββ*‐DLAMs as safe TLR4 agonists with minimized toxicity. The capacity of novel glycolipids to elicit the release of several important cytokines which provide a critical bridge between the innate and adaptive immunity as well as the possibility to modulate cellular responses by modification of the primary chemical structure renders *ββ*‐DLAMs eligible for future development as promising vaccine adjuvants and immunotherapeutics.

## Experimental Section

### General synthetic methods

Reagents and solvents were purchased from commercial suppliers and used without further purification unless otherwise stated. Toluene was dried by distillation first over phosphorus pentoxide, then over calcium hydride and was then stored over activated 4 Å molecular sieves (MS). Solvents were dried by storage over activated MS for at least 24 h prior to use (dichloromethane 4 Å, acetonitrile and DMF 3 Å). Residual moisture was determined by coloumbometric titration on a Mitsubishi CA21 Karl Fischer apparatus and did not exceed 20 ppm. Reactions were monitored by TLC performed on silica gel 60 F254 HPTLC precoated glass plates with a 25 mm concentration zone (Merck). Spots were visualized by dipping into a sulfuric acid‐*p*‐anisaldehyde solution and subsequent charring at 250 °C. Solvents were removed under reduced pressure at ≤40 °C. Preparative HPLC was performed on a YMC Pack SIL‐06 250×20 mm, S‐5 μm, 6 nm column or on a YMC Pack SIL‐06 250×10 mm, S‐5 μm, 6 nm column. Preparative MPLC and column chromatography were performed using silica gel 60 (0.040–0.063 mm). Size exclusion chromatography was performed using Bio‐Beads S−X1 support (BioRad). NMR spectra were recorded on a Bruker Avance III 600 spectrometer (^1^H at 600.22 MHz; ^13^C at 150.93 MHz; ^31^P at 242.97 MHz) using standard Bruker NMR software. Chemical shifts are reported in ppm, where ^1^H NMR spectra recorded from samples in CDCl_3_ were referenced to internal TMS and ^13^C spectra were referenced to the corresponding solvent signal (77.16 ppm for CDCl_3_). NMR spectra recorded from samples in other solvents were referenced to residual solvent signals (for CD_3_OD 3.31 and 49.00 ppm; for CD_2_Cl_2_ 5.32 and 53.84 ppm; for DMSO‐d_6_ 2.50 and 39.52 ppm; for ^1^H and ^13^C NMR, respectively). NMR spectra recorded in CDCl_3_‐MeOD (4 : 1, v/v) were referenced to residual solvent signals of CDCl_3_ (7.26 ppm and 77.16 ppm; ^1^H and ^13^C NMR, respectively). NMR spectra recorded in CDCl_3_: MeOD (1 : 1 to 4 : 1, v/v) were referenced to residual solvent signals of MeOD (3.31 and 49.00 ppm, ^1^H and ^13^C NMR, respectively). ^31^P NMR spectra were referenced according to IUPAC recommendations from a referenced ^1^H NMR spectrum. In all 1,1′‐disaccharides the NMR signals of the “distal” GlcN ring are indicated by primes. High‐resolution mass spectrometry (HRMS) was carried out on acetonitrile or DCM solutions via LC‐TOF MS (Agilent 1200SL HPLC and Agilent 6210 ESI‐TOF, Agilent Technologies). Datasets were analyzed using Agilent Mass Hunter Software. MALDI‐TOF MS was performed in negative‐ion mode using a Bruker Autoflex Speed instrument with 6‐aza‐2‐thiothymine (ATT) as matrix and ammonium sulfate as additive. Optical rotation was measured on a PerkinElmer 243B polarimeter equipped with a Haake water circulation bath and a Haake D1 immersion circulator for temperature control or an Anton Paar MCP 100 polarimeter featuring integrated peltier temperature control. All [α]^D^_20_ values are reported in units of deg dm^−1^ cm^3^ g^−1^, the corresponding concentrations are reported in g/100 mL.

**Ethyl 2‐Azido‐4,6‐*O*‐di‐*tert*‐butylsilylene‐2‐deoxy‐1‐thio‐α‐D‐gluco‐pyranoside (3)**: To a stirred solution of **2** (2.060 g, 8.26 mmol) in dry DMF (10 mL) dry pyridine (2 mL, 24.8 mmol) was added followed by di‐*tert*‐butylsilyl bis(trifluoromethanesulfonate) (4 g, 2.94 mL, 9.09 mmol) which was added in portions over a period of 5 min at −40 °C under atmosphere of Ar. The stirring was continued for 1 h at −40 °C and for 30 min at −20 °C, then the reaction was cooled to −40 °C and quenched by addition of MeOH (5 mL). The mixture was warmed to r.t., diluted with diethyl ether (250 mL) and washed with sat. aq. NaHCO_3_ (100 mL) and water (100 mL). The organic layer was dried over Na_2_SO_4_, filtered over cotton and concentrated. The residue was purified by column chromatography on silica gel (hexane‐EtOAc, 10 : 1, v/v) to give **3** as white solid (3.010 g, 93 %). *R*_f=_0.67 (toluene‐EtOAc, 6 : 1); [α]_D_^20^=+147 (c 1.0, CHCl_3_); ^1^H NMR (600 MHz, CDCl_3_): *δ* 5.30 (d, 1H, ^3^ *J*_1,2_=5.5 Hz, H‐1), 4.18 (ddd, 1H, ^3^ *J*_4,5_=^3^ *J*_5,6a_=10.0 Hz, ^3^ *J*_5,6b_=5.1 Hz, H‐5), 4.08 (dd, 1H, ^2^ *J*_6a,6b_=9.9 Hz, ^3^ *J*_5,6b_=5.0 Hz, H‐6b), 3.88 (dd, 1H, ^2^ *J*_6a,6b_=^3^ *J*_5,6a_=10.1 Hz, H‐6a), 3.82 (dd, 1H, ^3^ *J*_2,3_=10.0 Hz, ^3^ *J*_3,4_=8.5 Hz, H‐3), 3.77 (dd, 1H, ^3^ *J*_2,3_=10.1 Hz, ^3^ *J*_1,2_=5.5 Hz, H‐2), 3.68 (dd, 1H, ^3^ *J*_4,5_=9.4 Hz, ^3^ *J*_3,4_=8.6 Hz, H‐4), 2.67–2.55 (m, 2H, SC*H*_2_CH_3_), 1.30 (dd, 3H, ^3^ *J*=^3^ *J=*7.3 Hz, SCH_2_C*H*_3_), 1.05 (s, 9H, Si(C(C*H*_3_)_3_)_2_), 1.01 (s, 9H, Si(C(C*H*_3_)_3_)_2_); ^13^C NMR (151 MHz, CDCl_3_): *δ* 84.09 (CH, C‐1), 78.10 (CH, C‐4), 73.72 (CH, C‐3), 66.60 (CH, C‐5), 66.43 (CH_2_, C‐6), 63.08 (CH, C‐2), 27.55, 27.12 (CH_3_, Si(C(*C*H_3_)_3_)_2_), 25.25 (CH_2_, S*C*H_2_CH_3_), 22.82, 20.19 (C_q_, Si(*C*(CH_3_)_3_)_2_), 15.02 (CH_3_, SCH_2_ *C*H_3_); HRMS (ESI) *m/z* calcd. for [M+COOH]^−^ C_17_H_32_N_3_O_6_SSi 434.1787, found 434.1793.

**Ethyl 2‐azido‐3‐*O*‐[bis(benzyloxy)phosphoryl]‐4,6‐*O*‐di‐*tert*‐butylsilylene‐2‐deoxy‐1‐thio‐α‐D‐glucopyranoside (4)** To a stirred solution of **3** (505 mg, 1.30 mmol, dried by repeated co‐evaporation with dry toluene) in dry DCM (10 mL) bis(benzyloxy)(diisopropylamino)phosphine (1.119 mg, 3.24 mmol) and a solution of 1*H*‐tetrazol in acetonitrile (0.45 M; 7.54 mL, 3.24 mmol) were added successively under atmosphere of Ar. The stirring was continued for 30 min, the reaction mixture was cooled to −78 °C and a solution of 3−chloroperoxybenzoic acid (*m*−CPBA, 671 mg, 3.89 mmol) in dry DCM (1 mL) was added. The stirring was continued for 1 h at −78° C, then a solution of Et_3_N (3 equiv., 540 μL) in DCM (3 mL) was added at −78 °C and the reaction mixture was stirred for 20 min. The reaction mixture was allowed to warm up to rt, diluted with EtOAc (150 mL), and washed with TEAB buffer (2×50 mL), water (50 mL) and brine (50 mL). The organic layer was dried over Na_2_SO_4_, filtered over cotton and concentrated. The residue was purified via column chromatography on silica gel (hexane/EtOAc, 5 :1, v/v) to give **4** (760 mg, 90 %). *R*_f_=0.36 (toluene/EtOAc, 10 : 1); [α]_D_^20^=+79 (c 1.0, CHCl_3_); ^1^H NMR (600 MHz, CDCl_3_): *δ* 7.35–7.27 (m, 10H, Ph), 5.41 (d, 1H, ^3^ *J*_1,2_=5.8 Hz, H‐1), 5.15–5.06 (m, 4H, OP(O)(OC*H*_2_Ph)_2_), 4.56 (ddd, 1H, ^3^ *J*_2,3_=^3^ *J*_3,4_=^3^ *J*_3,P_=9.6 Hz, H‐3), 4.25 (ddd, 1H, ^3^ *J*_4,5_=^3^ *J*_5,6a_=10.1 Hz, ^3^ *J*_5,6b_=4.9 Hz, H‐5), 4.08 (dd, 1H, ^2^ *J*_6a,6b_=10.2 Hz, ^3^ *J*_5,6b_=4.9 Hz, H‐6b), 3.93–3.85 (m, 3H, H‐2, H‐4, H‐6a,), 2.67–2.58 (m, 2H, SC*H*_2_CH_3_), 1.31 (dd, 3H, ^3^ *J*=^3^ *J=*7.4 Hz, SCH_2_C*H*_3_), 1.01 (s, 9H, Si(C(C*H*_3_)_3_)_2_), 0.98 (s, 9H, Si(C(C*H*_3_)_3_)_2_); ^13^C NMR (151 MHz, CDCl_3_): *δ* 136.15, 136.10, 136.05 (C_q_, Ph), 128.60, 128.44, 127.97, 127.92 (CH, Ph), 84.02 (CH, C‐1), 79.43 (CH, d, ^2^ *J*_C,P_=6.6 Hz, C‐3), 76.65 (CH, d, ^3^ *J*_C,P_=3.3 Hz, C‐4), 69.49 (CH_2_, d, ^2^ *J*_C,P_=5.4 Hz, OP(O)(O*C*H_2_Ph)_2_), 69.33 (CH_2_, d, ^2^ *J*_C,P_=5.2 Hz, OP(O)(O*C*H_2_Ph)_2_), 67.05 (CH, C‐5), 66.32 (CH_2_, C‐6), 63.46 (CH, ^3^ *J*_C,P_=3.4 Hz, C‐2), 27.45, 27.04 (CH_3_, Si(C(*C*H_3_)_3_)_2_), 25.06 (CH_2_, S*C*H_2_CH_3_), 22.76, 20.21 (C_q_, Si(*C*(CH_3_)_3_)_2_), 14.96 (CH_3_, SCH_2_ *C*H_3_); ^31^P NMR (243 MHz, CDCl_3_): *δ* −1.62; HRMS (ESI) *m/z* calcd. for [M+COOH]^−^ C_31_H_45_N_3_O_9_PSSi 694.2389, found 694.2390.

**2‐Azido‐3‐*O*‐[bis(benzyloxy)phosphoryl]‐4,6‐*O*‐di‐*tert*‐butylsilylene‐2‐deoxy‐D‐glucopyranose (5)** To a stirred solution of **4** (1.07 g, 1.64 mmol) in acetone (10 mL), water (0.5 mL) was added and the mixture was cooled to 0 °C. *N*‐Bromosuccinimide (1.38 g, 7.73 mmol) was added in portions under stirring at 0 °C. The stirring was continued for 30 min, then the reaction mixture was quenched by addition of aq. 5 % Na_2_S_2_O_3_ ‐ sat. aq. NaHCO_3_ (1 : 1, 30 mL). The mixture was diluted with EtOAc (150 mL), and the organic layer was washed with water (30 mL) and brine (30 mL), dried over Na_2_SO_4_, filtered and concentrated. The residue was purified by MPLC (toluene–EtOAc (5→25 %)) to give **5** as white foam (918 mg, 92 %, α/β=1 : 1.1). *R*_f_=0.4 (toluene–EtOAc, 2 : 1); [α]_D_^20^=+1.0 (c 0.6, CHCl_3_); ^1^H NMR (600 MHz, CDCl_3_): *δ* 7.36–7.28 (m, 20H, Ph), 5.36 (d, 1H, ^3^ *J*_1α,2α_=3.8 Hz, H‐1α), 5.17–5.08 (m, 8H, OP(O)(OC*H*_2_Ph)_2_), 4.83 (ddd, 1H, ^3^ *J*_2α,3α_=^3^ *J*_3α,4α_=^3^ *J*_3α,P_=9.6 Hz, H‐3α), 4.77 (d, 1H, ^3^ *J*_1β,2β_=7.9 Hz, H‐1β), 4.32 (ddd, 1H, ^3^ *J*_2β,3β_=^3^ *J*_3β,4β_=^3^ *J*_3β,P_=9.5 Hz, H‐3β), 4.16 (dd, 1H, ^2^ *J*_6aβ,6bβ_=10.2 Hz, ^3^ *J*_5β,6bβ_=5.1 Hz, H‐6bβ), 4.13–4.07 (m, 2H, H‐5α, H‐6bα), 3.94–3.84 (m, 4H, H‐4α, H‐4β, H‐6aα, H‐6aβ), 3.46 (dd, 1H, ^3^ *J*_2β,3β_=10.1 Hz, ^3^ *J*_1β,2β_=7.9 Hz, H‐2β), 3.43 (ddd, 1H, ^3^ *J*_4β,5β_=^3^ *J*_5β,6aβ_=9.8 Hz, ^3^ *J*_5β,6bβ_=4.9 Hz, H‐5β), 3.30 (dd, 1H, ^3^ *J*_2α,3α_=10.2 Hz, ^3^ *J*_1α,2α_=3.5 Hz, H‐2α), 0.99 (s, 9H, Si(C(C*H*_3_)_3_)_2_), 0.98 (s, 9H, Si(C(C*H*_3_)_3_)_2_), 0.97 (s, 9H, Si(C(C*H*_3_)_3_)_2_), 0.96 (s, 9H, Si(C(C*H*_3_)_3_)_2_); ^13^C NMR (151 MHz, CDCl_3_): *δ* 136.03–135.84 (m, C_q_, Ph), 128.68, 128.64, 128.61, 128.55, 128.54, 128.49, 127.98, 127.91, 127.88 (CH, Ph), 96.88 (CH, C‐1β), 92.82 (CH, C‐1α), 80.53 (CH, d, ^2^ *J*_C,P_=6.5 Hz, C‐3β), 78.30 (CH, d, ^2^ *J*_C,P_=5.8 Hz, C‐3α), 76.57 (CH, d, ^3^ *J*_C,P_=3.1 Hz, C‐4α), 75.74 (CH, d, ^3^ *J*_C,P_=3.6 Hz, C‐4β), 70.63 (CH, C‐5β), 69.67, 69.63, 69.57, 69.54, 69.43, 69.42, 69.40, 69.38 (CH_2_, OP(O)(O*C*H_2_Ph)_2_), 66.58 (CH_2_, C‐6α), 66.55 (CH, m, C‐5α, C‐2β), 66.10 (CH_2_, C‐6β), 63.33 (CH, ^3^ *J*_C,P_=3.1 Hz, C‐2α), 27.49, 27.44, 27.05, 26.98 (CH_3_, Si(C(*C*H_3_)_3_)_2_), 22.80, 22.74, 20.10, 20.05 (C_q_, Si(*C*(CH_3_)_3_)_2_); ^31^P NMR (243 MHz, CDCl_3_): *δ* −1.69, 1.87; HRMS (ESI) *m/z* calcd. for [M−H]^−^ C_28_H_39_N_3_O_8_PSi 604.2250, found 604.2257.

**4,6‐*O*‐Benzylidene‐3‐*O***‐***tert*** **‐butyldimethylsilyl‐2‐deoxy‐2‐(2,2,2‐trichloroethoxycarbonylamino)‐β‐D‐glucopyranosyl‐(1↔1)‐2‐azido‐3‐*O*‐[bis(benzyloxy)phosphoryl]‐4,6‐*O*‐di‐*tert*‐butylsilylene‐2‐deoxy‐β‐D‐glucopyranoside (7)** To a stirred solution of glycosyl donor **6** ^[28]^ (720 mg, 0.98 mmol) glycosyl acceptor **5** (1200 mg, 1.98 mmol) in dry DCM (20 mL) and powdered 4 Å molecular sieves were added and the suspension was stirred for 1 h at rt. Then the mixture was cooled to 0 °C and TMSOTf (0.07 eq., 12.5 μL, 69 μmol) was added under atmosphere of Ar. The stirring was continued at 0 °C for 1.5 h, then Et_3_N (30 μL, 200 μmol) was added, and the mixture was warmed to r.t. The solids were removed by filtration over a pad of Celite, the filtrate was concentrated and co‐evaporated with toluene (2×50 mL). The residue was purified by MPLC (toluene–EtOAc, 10 : 1→10 : 5), mixed fractions were repurified (hexane–EtOAc, 4 : 1 and toluene–EtOAc, 10 : 1) to afford **7** (660 mg, 59 % calculated based on glycosyl donor; 65 % based on consumed glycosyl acceptor **5‐β**). Unreacted glycosyl acceptor **5** was recovered (595 mg, 1 mmol). *R*_f_=0.45 (toluene–EtOAc, 5 : 1); [α]_D_^20^=−24.0 (c 1.0, CHCl_3_); ^1^H NMR (600 MHz, CDCl_3_): *δ* 7.51–7.44 (m, 2H, Ph), 7.39–7.28 (m, 13H, Ph), 5.52 (s, 1H, OC*H*Ph), 5.15–5.03 (m, 6H, OP(O)(OC*H*_2_Ph)_2_, N*H*′, H‐1′), 4.80 (d, 1H, ^3^ *J*_1,2_=8.1 Hz, H‐1), 4.72–4.65 (m, 2H, C*H*_2ab_, Troc), 4.36 (dd, 1H, ^2^ *J*_6a′,6b′_=10.5 Hz, ^3^ *J*_5′,6b′_=5.0 Hz, H‐6b′), 4.31 (dd, 1H, ^3^ *J*_2,3_=^3^ *J*_3,4_=^3^ *J*_P,3_=9.5 Hz, H‐3), 4.19‐4.12 (m, 2H, H‐6b, H‐3′), 3.88 (dd, 1H, ^3^ *J*_3,4_=^3^ *J*_4,5_=9.3 Hz, H‐4), 3.85 (dd, 1H, ^2^ *J*_6a,6b_=^3^ *J*_5,6a_=10.0 Hz, H‐6a), 3.79 (dd, 1H, ^2^ *J*_6a′,6b′_=^3^ *J*_5′,6a′_=10.2 Hz, H‐6a′), 3.53 (dd, 1H, ^3^ *J*_3′,4′_=^3^ *J*_4′,5′_=9.0 Hz, H‐4′), 3.47 (m, 1H, H‐5′), 3.43–3.38 (m, 2H, H‐2, H‐5), 3.34 (m, H‐2′), 0.98 (s, 9H, Si(C(C*H*_3_)_3_)_2_), 0.96 (s, 9H, Si(C(C*H*_3_)_3_)_2_), 0.82 (s, 9H, Si(CH_3_)_2_C(C*H*_3_)_3_), 0.02 (s, 3H, Si(C*H*_3_)_2_C(CH_3_)_3_), −0.03 (s, 3H, Si(C*H*_3_)_2_C(CH_3_)_3_); ^13^C NMR (151 MHz, CDCl_3_): *δ* 153.92 (C=O, Troc), 137.20 (C_q_, Ph), 136.13–135.96 (C_q_, m, Ph), 129.23, 128.66, 128.61, 128.49, 128.46, 128.30, 127.89, 127.82, 126.41 (CH, Ph), 101.99 (CH, benzylidene acetal), 98.56 (CH, C‐1), 97.32 (CH, C‐1′), 95.22 (C_q_, Troc), 82.01 (CH, C‐4′), 80.00 (CH, d, ^2^ *J*_C,P_=5.7 Hz, C‐3), 75.52 (CH, C‐4), 74.81 (CH_2_, Troc), 71.41 (CH, C‐3′), 70.62 (CH, C‐5), 69.52 (CH_2_, d, ^2^ *J*_C,P_=5.4 Hz, OP(O)(O*C*H_2_Ph)_2_), 69.20 (CH_2_, d, ^2^ *J*_C,P_=5.3 Hz, OP(O)(O*C*H_2_Ph)_2_), 68.70 (CH_2_, C‐6′), 66.35 (CH, C‐5′), 65.93 (CH_2_, C‐6), 64.77 (CH, C‐2), 59.75 (CH, C‐2′), 27.43, 26.99 (CH_3_, Si(C(*C*H_3_)_3_)_2_), 25.85 (CH_3_, Si(CH_3_)_2_C(*C*H_3_)_3_), 22.75, 20.08 (C_q_, Si(*C*(CH_3_)_3_)_2_), 18.27 (C_q_, Si(CH_3_)_2_ *C*(CH_3_)_3_), −4.03, −4.88 (CH_3_, Si(*C*H_3_)_2_C(CH_3_)_3_); ^31^P NMR (243 MHz, CDCl_3_): *δ* −1.37; HRMS (ESI) *m/z* calcd. for [M+H]^+^ C_50_H_71_Cl_3_N_4_O_14_PSi_2_ 1143.3303, found 1143.3334.

**4,6‐*O*‐Benzylidene‐3‐*O***‐***tert*** **‐butyldimethylsilyl‐2‐deoxy‐2‐(2,2,2‐trichloroethoxycarbonylamino)‐β‐D‐glucopyranosyl‐(1↔1)‐2‐acetamido‐3‐*O*‐[bis(benzyloxy)phosphoryl]‐4,6‐*O*‐di‐*tert*‐butylsilylene‐2‐deoxy‐β‐D‐glucopyranoside (8)** To a stirred suspension of tin(II) thiophenolate complex [Sn(SPh)_2_] (147 mg, prepared from PhSH (4.5 equiv., 1.3 mmol, 132 μl) and Et_3_N (4.5 equiv., 1.3 mmol, 180 μL)) in dry toluene (10 mL) a solution of **7** (330 mg, 0.288 mmol) in dry toluene (5 mL) was added and the stirring was continued for 1.5 h at rt. The mixture was diluted with DCM (50 mL) and washed with aq. sodium hydroxide (2 M, 50 mL). The aq. layer was extracted with DCM (2×50 mL), the combined organic layers were washed with water (50 mL) and brine (50 mL), dried over Na_2_SO_4_, filtered over cotton and concentrated. The residue was dried in vacuo and dissolved in dry pyridine (5 mL). Acetic anhydride (20 equiv., 5,8 mmol, 550 μL) and DMAP (2 equiv., 0.58 mmol, 71 mg) were added at 0 °C and the mixture was stirred for 2 h at rt. under atmosphere of Ar. The reaction mixture was diluted with EtOAc (100 mL), washed with aq. HCl (1 M, 100 mL), the aq. layer was washed with EtOAc (50 mL), the combined organic phases were washed with water (100 mL), sat. aq. NaHCO_3_ (100 mL) and brine (100 mL), dried over Na_2_SO_4_, filtered over cotton and concentrated. The residue was purified by column chromatography on silica gel (toluene/EtOAc, 3 : 1→2 : 1, v/v) to give **8** as white solid (286 mg, 85 %). *R*_f_=0.42 (toluene/acetone, 3 : 1); [α]_D_^20^=−24 (c 1.0, CHCl_3_); ^1^H NMR (600 MHz, CDCl_3_): *δ* 7.49–7.44 (m, 2H, Ph), 7.39–7.28 (m, 13H, Ph), 6.11 (d, 1H, ^3^ *J*_NH,2_=9.1 Hz, N*H*), 5.48 (s, 1H, OC*H*Ph), 5.41 (d, 1H, ^3^ *J*_NH′,2′_=7.2 Hz, N*H*′), 5.15‐5.10 (m, 3H, H‐1′, OP(O)(OC*H*_2_Ph)_2_), 4.98 (d, 2H, ^3^ *J*_P,H_=6.7 Hz, OP(O)(OC*H*_2_Ph)_2_), 4.78 (d, 1H, ^3^ *J*_1,2_=8.4 Hz, H‐1), 4.72 (d, 1H, ^2^ *J*=11.9 Hz, C*H*_2b_, Troc), 4.63‐4.54 (m, 2H, C*H*_2a_, Troc, H‐3), 4.36 (dd, 1H, ^3^ *J*_2′,3′_=^3^ *J*_3′,4′_=8.7 Hz, H‐3′), 4.31 (dd, 1H, ^2^ *J*_6a′,6b′_=10.4 Hz, ^3^ *J*_5′,6b′_=4.7 Hz, H‐6b′), 4.15 (dd, 1H, ^2^ *J*_6a,6b_=10.4 Hz, ^3^ *J*_5,6b_=4.9 Hz, H‐6b), 4.02 (ddd, 1H, ^3^ *J*_1,2_=^3^ *J*_2,3_=^3^ *J*_2,NH_=9.3 Hz, H‐2), 3.96 (dd, 1H, ^3^ *J*_3,4_=^3^ *J*_4,5_=9.3 Hz, H‐4), 3.86 (dd, 1H, ^2^ *J*_6a,6b_= ^3^ *J*_5,6a_=10.2 Hz[AZ7] , H‐6a), 3.69 (dd, 1H, ^2^ *J*_6a′,6b′_=^3^ *J*_5′,6a′_=9.9 Hz, H‐6a′), 3.50‐3.40 (m, 3H, H‐5, H‐4′, H‐5′), 3.09 (m, 1H, H‐2′), 1.85 (s, 3H, C*H*_3_, NHAc), 0.98 (s, 9H, Si(C(C*H*_3_)_3_)_2_), 0.95 (s, 9H, Si(C(C*H*_3_)_3_)_2_), 0.81 (s, 9H, Si(CH_3_)_2_C(C*H*_3_)_3_), 0.01 (s, 3H, Si(C*H*_3_)_2_C(CH_3_)_3_), −0.05 (s, 3H, Si(C*H*_3_)_2_C(CH_3_)_3_); ^13^C NMR (151 MHz, CDCl_3_): *δ* 171.12 (C=O, NH*Ac*), 153.88 (C=O, Troc), 137.25 (C_q_, Ph), 135.81 (C_q_, d, ^3^ *J*_C,P_=7.8 Hz, Ph), 135.61 (C_q_, d, ^3^ *J*_C,P_=7.9 Hz, Ph), 129.19, 128.67, 128.64, 128.56, 128.28, 128.12, 127.88, 127.78, 126.40 (CH, Ph), 101.94 (CH, benzylidene acetal), 98.59 (CH, C‐1), 97.02 (CH, C‐1′), 95.48 (C_q_, Troc), 82.33 (CH, C‐4′), 80.18 (CH, d, ^2^ *J*_C,P_=6.6 Hz, C‐3), 75.85 (CH, ^3^ *J*_C,P_=4.6 Hz, C‐4), 74.72 (CH_2_, Troc), 70.65, 70.54 (CH, C‐5, C‐3′), 69.93 (CH_2_, d, ^2^ *J*_C,P_=6.4 Hz, OP(O)(O*C*H_2_Ph)_2_), 69.34 (CH_2_, d, ^2^ *J*_C,P_=6.9 Hz, OP(O)(O*C*H_2_Ph)_2_), 68.75 (CH_2_, C‐6′), 66.21 (CH_2_, C‐6), 66.03 (CH, C‐5′), 60.03 (CH, C‐2′), 54.80 (CH, C‐2), 27.49, 27.03 (CH_3_, Si(C(*C*H_3_)_3_)_2_), 25.85 (CH_3_, Si(CH_3_)_2_C(*C*H_3_)_3_), 23.53 (CH_3_, NH*Ac*), 22.75, 20.04 (C_q_, Si(*C*(CH_3_)_3_)_2_), 18.26 (C_q_, Si(CH_3_)_2_ *C*(CH_3_)_3_), −4.10, −4.94 (CH_3_, Si(*C*H_3_)_2_C(CH_3_)_3_); ^31^P NMR (243 MHz, CDCl_3_): *δ* −1.83; HRMS (ESI) *m/z* calcd. for [M+H]^+^ C_52_H_75_Cl_3_N_2_O_15_PSi_2_ 1159.3504, found 1159.3542.

**4,6‐*O*‐Benzylidene‐3‐*O***‐***tert*** **‐butyldimethylsilyl‐2‐deoxy‐2‐(2,2,2‐trichloroethoxycarbonylamino)‐β‐D‐glucopyranosyl‐(1↔1)‐2‐acetamido‐3‐*O*‐[bis(benzyloxy)phosphoryl]‐2‐deoxy‐β‐D‐glucopyranoside (9)** To a stirred solution of **8** (122 mg, 0.11 mmol) in dry THF (10 mL) in a PTFE flask a solution of HF⋅Py (70 %, 86 μL) was added at 0 °C. The stirring was continued for 1 h, the reaction mixture was diluted with DCM (100 mL), washed with sat. aq. NaHCO_3_ (50 mL), the aqueous layer was extracted with DCM (2×50 mL), the combined organic layers were dried over Na_2_SO_4_, filtered over cotton and concentrated. The residue was purified by column chromatography on silica gel (1. EtOAc; 2. DCM/MeOH 3.5 %) to give **9** (93 mg, 87 %). *R*_f_=0.3 (EtOAc); *R*_f_=0.23 (DCM/MeOH, 95 : 5); [α]_D_^20^=−9.6 (c 1.0, CHCl_3_); ^1^H NMR (600 MHz, CDCl_3_): *δ* 7.48–7.41 (m, 2H, Ph), 7.38–7.28 (m, 13H, Ph), 6.12 (d, 1H, ^3^ *J*_NH,2_=7.8 Hz, N*H*), 5.66 (d, 1H, ^3^ *J*_NH′,2′_=7.4 Hz, N*H*′), 5.45 (s, 1H, OC*H*Ph), 5.22 (d, 1H, ^3^ *J*_1′,2′_=8.6 Hz, H‐1′), 5.09–4.97 (m, 4H OP(O)(OC*H*_2_Ph)_2_), 4.88 (d, 1H, ^3^ *J*_1,2_=8.3 Hz, H‐1), 4.74–4.60 (m, 3H, C*H*_2ab_, Troc, H‐3), 4.33 (br, 1H, 4‐O*H*), 4.30–4.17 (m, 3H, H‐6b′, H‐3′), 3.92 (m, 1H, H‐6b), 3.74–3.64 (m, 3H, H‐2, H‐6a, H‐6a′), 3.56 (m, 1H, H‐4), 3.51–3.37 (m, 3H, H‐5, H‐4′, H‐5′), 3.11 (m, 1H, H‐2′), 2.87 (br, 1H, 6‐O*H*) 1.79 (s, 3H, C*H*_3_, NHAc), 0.81 (s, 9H, Si(CH_3_)_2_C(C*H*_3_)_3_), 0.01 (s, 3H, Si(C*H*_3_)_2_C(CH_3_)_3_), −0.05 (s, 3H, Si(C*H*_3_)_2_C(CH_3_)_3_); ^13^C NMR (151 MHz, MeOD): *δ* 173.84 (C=O, NH*Ac*), 156.92 (C=O, Troc), 139.13 (C_q_, Ph), 137.55 (C_q_, d, ^3^ *J*_C,P_=7.8 Hz, Ph), 137.38 (C_q_, d, ^3^ *J*_C,P_=7.9 Hz, Ph), 130.17, 130.07, 129.75, 129.71, 129.66, 129.36, 129.25, 125.25, 129,19, 129.15, 127.75, 127.69 (CH, Ph), 103.37 (CH, benzylidene acetal), 100.12 (CH, C‐1′), 99.11 (CH, C‐1), 97.20 (C_q_, Troc), 83.46 (CH, C‐4′), 82.93 (CH, d, ^2^ *J*_C,P_=6.6 Hz, C‐3), 78.05 (CH, C‐5), 75.96 (CH_2_, Troc), 74.14 (CH, C‐3′), 71.27 (CH, ^3^ *J*_C,P_=4.6 Hz, C‐4), 71.0 (CH_2_, d, ^2^ *J*_C,P_=6.4 Hz, OP(O)(O*C*H_2_Ph)_2_), 70.96 (CH_2_, d, ^2^ *J*_C,P_=6.9 Hz, OP(O)(O*C*H_2_Ph)_2_), 66.21 (CH_2_, C‐6′), 67.82 (CH, C‐5′), 63.11 (CH_2_, C‐6), 60.14 (CH, C‐2′), 54.49 (CH, C‐2), 26.51 (CH_3_, Si(CH_3_)_2_C(*C*H_3_)_3_ ), 23.31 (CH_3_, NH*Ac*), 19.21 (C_q_, Si(*C*(CH_3_)_3_)_2_), −3.76, −4.42 (CH_3_, Si(*C*H_3_)_2_C(CH_3_)_3_); ^31^P NMR (243 MHz, MeOD): *δ* −2.08; HRMS (ESI) *m/z* calcd. for [M+COOH]^−^ C_45_H_59_Cl_3_N_2_O_17_PSi 1063.2392, found 1063.2391.

**2‐Amino‐4,6‐*O*‐benzylidene‐3‐*O***‐***tert*** **‐butyldimethylsilyl‐2‐deoxy‐β‐D‐glucopyranosyl‐(1↔1)‐2‐acetamido‐3‐*O*‐[bis(benzyloxy)phosphoryl]‐4,6‐*O*‐di‐*tert*‐butylsilylene‐2‐deoxy‐β‐D‐glucopyranoside (16)** To a stirred solution of **8** (225 mg, 0.194 mmol) in acetic acid (ultra‐pure, 7 mL) Zn powder (10 μm, 1.5 g, 120 eq.) was added in 3 equal portions over a period of 1 h at 0° C. The reaction mixture was stirred for another 30 min at 0 °C under sonication, then diluted with DCM (50 mL), and the solids were removed by filtration over a pad of Celite. The filtrate was diluted with DCM (50 mL) and washed with sat. aq. NaHCO_3_ (3×30 mL), and brine (30 mL). The organic layer was dried over Na_2_SO_4_, filtered over cotton and concentrated. The residue was purified by flash chromatography on silica gel (1. toluene/EtOAc, 3 : 2, v/v; 2. toluene/MeOH/water/HCOOH, 40 : 1:0.2:0.2 → 20 : 1:0.2:0.1) to give **16** (175 mg, 91 %). *R*_f_=0.5 (toluene/EtOAc, 1 : 1); [α]_D_^20^=−21 (c 1.0, CHCl_3_); ^1^H NMR (600 MHz, CDCl_3_): *δ* 7.50–7.42 (m, 2H, Ph), 7.39–7.25 (m, 13H, Ph), 5.99 (d, 1H, ^3^ *J*_NH,2_=9.3 Hz, N*H*), 5.47 (s, 1H, OC*H*Ph), 5.14–5.10 (m, 2H, OP(O)(OC*H*_2_Ph)_2_), 5.01–4.95 (m, 2H, OP(O)(OC*H*_2_Ph)_2_), 4.75 (d, 1H, ^3^ *J*_1,2_=8.4 Hz, H‐1), 4.58 (d, 1H, ^3^ *J*_1′,2′_=8.1 Hz, H‐1′), 4.54 (m, 1H, H‐3), 4.29 (dd, 1H, ^2^ *J*_6a′,6b′_=10.4 Hz, ^3^ *J*_5′,6b′_=4.9 Hz, H‐6b′), 4.22 (dd, 1H, ^2^ *J*_6a,6b_=10.5 Hz, ^3^ *J*_5,6b_=5.0 Hz, H‐6b), 4.12 (ddd, 1H, ^3^ *J*_1,2_=^3^ *J*_2,3_=^3^ *J*_2,NH_=9.5 Hz, H‐2), 4.01 (dd, 1H, ^3^ *J*_3,4_=^3^ *J*_4,5_=9.2 Hz, H‐4), 3.94 (dd, 1H, ^2^ *J*_6a,6b_=^3^ *J*_5,6a_=10.3 Hz, H‐6a), 3.71 (dd, 1H, ^2^ *J*_6a′,6b′_=^3^ *J*_5′,6a′_=10.2 Hz, H‐6a′), 3.65 (dd, 1H, ^3^ *J*_2′,3′_=^3^ *J*_3′,4′_=9.0 Hz, H‐3′), 3.51–3.44 (m, 2H, H‐5, H‐4′), 3.40 (ddd, 1H, ^3^ *J*_4′,5′_=^3^ *J*_5′,6a′_=9.6 Hz, ^3^ *J*_5′,6b′_=5.0 Hz, H‐5′), 2.84 (dd, 1H, ^3^ *J*_1′,2′_=^3^ *J*_2′,3′_=8.6 Hz, H‐2′), 1.85 (s, 3H, C*H*_3_, NHAc), 0.99 (s, 9H, Si(C(C*H*_3_)_3_)_2_), 0.96 (s, 9H, Si(C(C*H*_3_)_3_)_2_), 0.84 (s, 9H, Si(CH_3_)_2_C(C*H*_3_)_3_), 0.07 (s, 3H, Si(C*H*_3_)_2_C(CH_3_)_3_), −0.03 (s, 3H, Si(C*H*_3_)_2_C(CH_3_)_3_); ^13^C NMR (151 MHz, CDCl_3_): *δ* 170.96 (C=O, NH*Ac*), 137.28 (C_q_, Ph), 135.85 (C_q_, d, ^3^ *J*_C,P_=7.6 Hz, Ph), 135.62 (C_q_, d, ^3^ *J*_C,P_=7.7 Hz, Ph), 129.20, 128.66, 128.63, 128.53, 128.28, 128.12, 127.73, 126.44 (CH, Ph), 102.17 (CH, benzylidene acetal), 100.77 (CH, C‐1′), 98.35 (CH, C‐1), 81.66 (CH, C‐4′), 80.27 (CH, d, ^2^ *J*_C,P_=6.5 Hz, C‐3), 75.86 (CH, ^3^ *J*_C,P_=4.8 Hz, C‐4), 74.89 (CH, C‐3′), 70.89 (CH, C‐5), 69.90 (CH_2_, d, ^2^ *J*_C,P_=6.3 Hz, OP(O)(O*C*H_2_Ph)_2_), 69.27 (CH_2_, d, ^2^ *J*_C,P_=6.3 Hz, OP(O)(O*C*H_2_Ph)_2_), 68.80 (CH_2_, C‐6′), 66.74 (CH, C‐5′), 66.26 (CH_2_, C‐6), 58.33 (CH, C‐2′), 54.65 (CH, C‐2), 27.50, 27.04 (CH_3_, Si(C(*C*H_3_)_3_)_2_), 26.00 (CH_3_, Si(CH_3_)_2_C(*C*H_3_)_3_), 23.53 (CH_3_, NH*Ac*), 22.76, 20.04 (C_q_, Si(*C*(CH_3_)_3_)_2_) 18.35 (C_q_, Si(CH_3_)_2_ *C*(CH_3_)_3_), −3.85, −4.72 (CH_3_, Si(*C*H_3_)_2_C(CH_3_)_3_); ^31^P NMR (243 MHz, CDCl_3_): *δ* −1.62; HRMS (ESI) *m/z* calcd. for [M+COOH]^−^ C_50_H_74_N_2_O_15_PSi_2_ 1029.4371, found 1029.4387.

**4,6‐*O*‐Benzylidene‐3‐*O***‐***tert*** **‐butyldimethylsilyl‐2‐deoxy‐2‐[(*R*)‐3‐(dodecanoyloxy)tetradecanoylamino]‐β‐D‐glucopyranosyl‐(1↔1)‐2‐acetamido‐3‐*O*‐[bis(benzyloxy)phosphoryl]‐4,6‐*O*‐di‐*tert*‐butylsilylene‐2‐deoxy‐β‐D‐glucopyranoside (17)** To a stirred solution of **16** (400 mg, 0.406 mmol) in dry DCM (4 mL) (*R*)‐3‐(dodecanoyloxy)tetradecanoic acid **FA5** (2.2 eq., 0.89 mmol, 385 mg) and EDC ⋅ HCl (3,1 eq., 240 mg, 1,26 mmol) were added in 0.5 eq. portions over a period of 3 h under atmosphere of Ar. The stirring was continued for additional 1 h, the reaction mixture was diluted with DCM (150 mL), washed with sat. aq. NaHCO_3_ (2×50 mL) and brine (50 mL), filtered over cotton and concentrated. The residue was purified by MPLC (toluene ‐ EtOAc, 3 : 1 → 1 : 1, v/v) to give **17** (420 mg, 74 %). *R*_f_=0.4 (hexane ‐ EtOAc, 1 : 1); [α]_D_^20^=−19 (c 1.0); ^1^H NMR (600 MHz, CDCl_3_): *δ* 7.48–7.42 (m, 2H, Ph), 7.38–7.27 (m, 13H, Ph), 5.98 (d, 1H, ^3^ *J*_NH′,2′_=7.4 Hz, N*H*′), 5.83 (d, 1H, ^3^ *J*_NH,2_=9.2 Hz, N*H*), 5.46 (s, 1H, OC*H*Ph), 5.28 (d, 1H, ^3^ *J*_1′,2′_=8.4 Hz, H‐1′), 5.16‐5.09 (m, 3H, β^Myr^−C*H*, OP(O)(OC*H*_2_Ph)_2_), 5.00–4.95 (m, 2H, OP(O)(OC*H*_2_Ph)_2_), 4.72 (d, 1H, ^3^ *J*_1,2_=8.4 Hz, H‐1), 4.57 (m, 1H, H‐3), 4.52 (dd, 1H, ^3^ *J*_2′,3′_=^3^ *J*_3′,4′_=9.0 Hz, H‐3′), 4.30 (dd, 1H, ^2^ *J*_6a′,6b′_=10.3 Hz, ^3^ *J*_5′,6b′_=5.0 Hz, H‐6b′), 4.18 (dd, 1H, ^2^ *J*_6a,6b_=10.3 Hz, ^3^ *J*_5,6b_=5.0 Hz, H‐6b), 3.98 (m, 1H, H‐2), 3.94 (dd, 1H, ^3^ *J*_3,4_=^3^ *J*_4,5_=9.2 Hz, H‐4), 3.84 (dd, 1H, ^2^ *J*_6a,6b_=^3^ *J*_5,6a_=10.2 Hz, H‐6a), 3.66 (dd, 1H, ^2^ *J*_6a′,6b′_=^3^ *J*_5′,6a′_=10.2 Hz, H‐6a′), 3.50 (ddd, 1H, ^3^ *J*_4′,5′_=^3^ *J*_5′,6a′_=9.7 Hz, ^3^ *J*_5′,6b′_=4.9 Hz, H‐5′), 3.45 (ddd, 1H, ^3^ *J*_4,5_=^3^ *J*_5,6a_=9.8 Hz, ^3^ *J*_5,6b_=5.0 Hz, H‐5), 3.36 (dd, 1H, ^3^ *J*_3′,4′_=^3^ *J*_4′,5′_=9.1 Hz, H‐4′), 3.03 (ddd, 1H, ^3^ *J*_1′,2′_=^3^ *J*_2′,3′_=^3^ *J*_NH′,2′_=8.1 Hz, H‐2′), 2.45 (dd, 1H, ^2^ *J*=15.5 Hz, ^3^ *J*=5.8 Hz, α^Myr^−C*H*_2_), 2.37 (dd, 1H, ^2^ *J*=15.3 Hz, ^3^ *J*=6.2 Hz, α^Myr^−C*H*_2_), 2.32–2.27 (m, 2H, α^Lau^−C*H*_2_), 1.84 (s, 3H, C*H*_3_, NHAc), 1.67–1.58 (m, 4H, β^Lau^−C*H*_2_, γ^Myr^−C*H*_2_), 1.37–1.19 (m, 34H, C*H*_2_), 0.98 (s, 9H, Si(C(C*H*_3_)_3_)_2_), 0.95 (s, 9H, Si(C(C*H*_3_)_3_)_2_), 0.89–0.85 (m, 6H, ω^Myr^−C*H*_3_, ω^Lau^−C*H*_3_), 0.80 (s, 9H, Si(CH_3_)_2_C(C*H*_3_)_3_), −0.08 (s, 3H, Si(C*H*_3_)_2_C(CH_3_)_3_), −0.03 (s, 3H, Si(C*H*_3_)_2_C(CH_3_)_3_); ^13^C NMR (151 MHz, CDCl_3_): *δ* 173.52 (C=O, Lau), 170.95 (C=O, NH*Ac*), 170.11 (C=O, Myr), 137.35 (C_q_, Ph), 135.89 (C_q_, d, ^3^ *J*_C,P_=8.3 Hz, Ph), 135.67 (C_q_, d, ^3^ *J*_C,P_=8.0 Hz, Ph), 129.18, 128.67, 128.64, 128.54, 128.28, 128.09, 127.75, 126.45 (CH, Ph), 102.00 (CH, benzylidene acetal), 98.90 (CH, C‐1), 96.93 (CH, C‐1′), 82.65 (CH, C‐4′), 80.14 (CH, d, ^2^ *J*_C,P_=6.0 Hz, C‐3), 76.02 (CH, ^3^ *J*_C,P_=4.7 Hz, C‐4), 70.83 (β^Myr^−CH), 70.77 (CH, C‐5), 70.38 (CH, C‐3′), 69.83 (CH_2_, d, ^2^ *J*_C,P_=6.6 Hz, OP(O)(O*C*H_2_Ph)_2_), 69.27 (CH_2_, d, ^2^ *J*_C,P_=5.5 Hz, OP(O)(O*C*H_2_Ph)_2_), 68.85 (CH_2_, C‐6′), 66.27 (CH_2_, C‐6), 65.83 (CH, C‐5′), 60.26 (CH, C‐2′), 54.88 (CH, C‐2), 41.54 (α^Myr^−CH_2_), 34.74 (α^Lau^−CH_2_), 33.91, 32.07, 32.06, 29.88, 29.87, 29.82, 29.79, 29.77, 29.73, 29.68, 29.52, 29.49, 29.37 (CH_2,_ Myr, Lau), 27.50, 27.03 (CH_3_, Si(C(*C*H_3_)_3_)_2_), 25.89 (CH_3_, Si(CH_3_)_2_C(*C*H_3_)_3_), 25.55, 25.17 (CH_2,_ Myr, Lau), 23.57 (CH_3_, NH*Ac*), 22.83 (CH_2,_ Myr, Lau), 22.77, 20.06 (C_q_, Si(*C*(CH_3_)_3_)_2_), 18.24 (C_q_, Si(CH_3_)_2_ *C*(CH_3_)_3_), 14.25 (ω^Myr^−CH_3_, ω^Lau^−CH_3_), −4.07, −4.76 (CH_3_, Si(*C*H_3_)_2_C(CH_3_)_3_); ^31^P NMR (243 MHz, CDCl_3_): *δ* −1.50; HRMS (ESI) *m/z* calcd. for [M+COOH]^−^ C_76_H_122_N_2_O_18_PSi_2_ 1437.7974, found 1437.7998.

**4,6‐*O*‐Benzylidene‐3‐*O***‐***tert*** **‐butyldimethylsilyl‐2‐deoxy‐2‐[(*R*)‐3‐(dodecanoyloxy)tetradecanoylamino]‐β‐D‐glucopyranosyl‐(1↔1)‐2‐acetamido‐3‐*O*‐[bis(benzyloxy)phosphoryl]‐2‐deoxy‐β‐D‐glucopyranoside (18)** To a stirred solution of **17** (360 mg, 0.26 mmol) in dry THF (20 mL) in a PTFE flask a solution of HF⋅Py (70 %, 150 μL) was added at 0 °C. The stirring was continued for 1 h, the reaction mixture was diluted with DCM (100 mL), washed with sat. aq. NaHCO_3_ (50 mL), the aqueous layer was extracted with DCM (2×50 mL), the combined organic layers were dried over Na_2_SO_4_, filtered over cotton and concentrated. The residue was purified by column chromatography on silica gel (1. Toluene ‐ MeOH, 2 : 1, v/v, 2. Chloroform ‐ MeOH, 60 : 1→40 : 1, v/v) to give **18** (280 mg, 86 %). *R*_f_=0.31 (DCM ‐ methanol (5 %)); [α]_D_^20^=−10.3 (c 1.0, CHCl_3_); ^1^H NMR (600 MHz, CDCl_3_): *δ* 7.46–7.40 (m, 2H, Ph), 7.37–7.27 (m, 13H, Ph), 6.28 (d, 1H, ^3^ *J*_NH′,2′_=7.8 Hz, N*H*′), 6.22 (d, 1H, ^3^ *J*_NH,2_=8.3 Hz, N*H*), 5.42 (s, 1H, OC*H*Ph), 5.33 (d, 1H, ^3^ *J*_1′,2′_=8.4 Hz, H‐1′), 5.15 (m, 1H, β^Myr^−C*H*), 5.09–4.96 (m, 4H, OP(O)(OC*H*_2_Ph)_2_), 4.86 (d, 1H, ^3^ *J*_1,2_=8.4 Hz, H‐1), 4.76 (m, 1H, H‐3), 4.34 (d, 1H, ^3^ *J*_OH,4_=5.1 Hz, 4‐O*H*), 4.25 (dd, 1H, ^3^ *J*_2′,3′_=^3^ *J*_3′,4′_=9.0 Hz, H‐3′), 4.20 (dd, 1H, ^2^ *J*_6a′,6b′_=10.4 Hz, ^3^ *J*_5′,6b′_=4.7 Hz, H‐6b′), 3.93 (ddd, 1H, ^2^ *J*_6a,6b_=11.8 Hz, ^3^ *J*_OH,6b_=8.9 Hz, ^3^ *J*_5,6b_=2.6 Hz, H‐6b), 3.71–3.60 (m, 3H, H‐2, H‐6a, H‐6a′), 3.55–3.47 (m, 2H, H‐4, H‐5), 3.44 (ddd, 1H, ^3^ *J*_4′,5′_=^3^ *J*_5′,6a′_=9.8 Hz, ^3^ *J*_5′,6b′_=4.9 Hz, H‐5′), 3.37 (dd, 1H, ^3^ *J*_3′,4′_=^3^ *J*_4′,5′_=9.0 Hz, H‐4′), 3.28 (dd, 1H, ^3^ *J*_OH,6b_=8.6 Hz, ^3^ *J*_OH,6a_=5.6 Hz, 6‐O*H*), 3.21 (m, H‐2′), 2.49–2.39 (m, 2H, α^Myr^−C*H*_2_), 2.33–2.29 (m, 2H, α^Lau^−C*H*_2_), 1.78 (s, 3H, C*H*_3_, NHAc), 1.70–1.57 (m, 4H, β^Lau^−C*H*_2_, γ^Myr^−C*H*_2_), 1.38–1.18 (m, 34H, C*H*_2_), 0.90–0.84 (m, 6H, ω^Myr^−C*H*_3_, ω^Lau^−C*H*_3_), 0.80 (s, 9H, Si(CH_3_)_2_C(C*H*_3_)_3_), −0.03 (s, 3H, Si(C*H*_3_)_2_C(CH_3_)_3_), −0.08 (s, 3H, Si(C*H*_3_)_2_C(CH_3_)_3_); ^13^C NMR (151 MHz, CDCl_3_): δ 174.20, 170.90, 170.69 (C=O, Myr, Lau, NHAc), 137.29 (C_q_, Ph), 135.67‐135.57 (C_q_, m, Ph), 129.18, 128.88, 128.81, 128.76, 128.74, 128.26, 128.19, 128.14, 126.47, 126.36 (CH, Ph), 102.04 (CH, benzylidene acetal), 98.21 (CH, C‐1), 97.59 (CH, C‐1′), 82.52 (CH, C‐4′), 81.05 (CH, d, ^2^ *J*_C,P_=6.1 Hz, C‐3), 75.84 (CH, C‐5), 71.28‐71.14 (CH, m, C‐4, C‐3′, β^Myr^−CH), 70.06 (CH_2_, d, ^2^ *J*_C,P_=6.0 Hz, OP(O)(O*C*H_2_Ph)_2_), 68.70 (CH_2_, C‐6′), 66.18 (CH, C‐5′), 62.84 (CH_2_, C‐6), 59.62 (CH, C‐2′), 55.60 (CH, C‐2), 42.14 (α^Myr^−CH_2_), 34.78, 34.41, 32.05, 29.82, 29.78, 29.76, 29.66, 29.65, 29.50, 29.48, 29.46, 29.34 (CH_2,_ Myr, Lau), 25.89 (CH_3_, Si(CH_3_)_2_C(*C*H_3_)_3_), 25.44, 25.12 (CH_2,_ Myr, Lau), 23.43 (CH_3_, NH*Ac*), 22.81 (CH_2,_ Myr, Lau), 18.22 (C_q_, Si(CH_3_)_2_ *C*(CH_3_)_3_), 14.24 (ω^Myr^−CH_3_, ω^Lau^−CH_3_), −4.03, −4.69 (CH_3_, Si(*C*H_3_)_2_C(CH_3_)_3_); ^31^P NMR (243 MHz, CDCl_3_): δ −0.57; HRMS (ESI) *m/z* calcd. for [M−H]^−^ C_67_H_104_ N_2_O_16_PSi 1251.6898, found 1251.6924.

**4,6‐*O*‐Benzylidene‐3‐*O***‐***tert*** **‐butyldimethylsilyl‐2‐deoxy‐2‐[(*R*)‐3‐(dodecanoyloxy)tetradecanoylamino]‐β‐D‐glucopyranosyl‐(1↔1)‐2‐acetamido‐3‐*O*‐[bis(benzyloxy)phosphoryl]‐2‐deoxy‐6‐*O*‐[(*R*)‐3‐(dodecanoyloxy)tetradecanoyl]‐β‐D‐glucopyranoside (24)** To a stirred solution of **18** (97 mg, 0.077 mmol) and (*R*)‐3‐(dodecanoyloxy)tetradecanoic acid **FA5** (36 mg, 0.085 mmol) in dry DCM (3 mL) a solution of DIC (20 mg, 0.16 mmol) in dry DCM (0.1 mL) and DMAP (0.05 eq; 0.002 mmol) were successfully added at 0° C and the stirring was continued for 20 min. The reaction mixture was brought up to r.t. and the stirring was continued for 4 h. The mixture was diluted with EtOAc (50 mL) and washed with sat. aq. NaHCO_3_ (20 mL), water (20 mL) and brine (20 mL). The organic layer was dried over Na_2_SO_4_, filtered and concentrated. The residue was purified by HPLC (1. toluene–EtOAc, 3 : 1→2 : 1, v/v; 2. hexane–EtOAc, 1 : 1→0 : 1, v/v) and size‐exclusion chromatography on Bio‐Beads S−X1 support (toluene–DCM, 3 : 1, v/v) to give **24** (96 mg, 75 %). R_f_=0.32 (EtOAc ‐ toluene, 2 : 1); [α]_D_^20^=−17.6 (c 0.9, CHCl_3_); ^1^H NMR (600 MHz, CDCl_3_): δ 7.47‐7.41 (m, 2H, Ph), 7.38‐7.28 (m, 13H, Ph), 6.49 (d, 1H, ^3^ *J*_NH′,2′_=8.3 Hz, N*H*′), 6.34 (d, 1H, ^3^ *J*_NH,2_=7.1 Hz, N*H*), 5.46 (s, 1H, OC*H*Ph), 5.28 (m, 1H, β^Myr^−C*H*), 5.18 (m, 1H, β^Myr^−C*H*), 5.12‐4.92 (m, 7H, H‐1, H‐3, H‐1′, OP(O)(OC*H*_2_Ph)_2_), 4.59 (d, 1H, ^3^ *J*_OH,4_=4.2 Hz, 4‐O*H*), 4.37 (m, 1H, H‐6b), 4.27–4.17 (m, 2H, H‐6a, H‐6b′) 4.12 (m, 1H, H‐3′), 3.65 (dd, 1H, ^2^ *J*_6a′,6b′_=^3^ *J*_5′,6a′_=10.1 Hz, H‐6a′), 3.63–3.51 (m, 3H, H‐4, H‐5, H‐2′), 3.47‐3.34 (m, 3H, H‐2, H‐4′, H‐5′), 2.62‐2.53 (m, 2H, α^Myr^−C*H*_2_), 2.49 (dd, 1H, ^2^ *J*=15.1 Hz, ^3^ *J*=5.7 Hz, α^Myr^−C*H*_2_), 2.39 (dd, 1H, ^2^ *J*=15.1 Hz, ^3^ *J*=6.9 Hz, α^Myr^−C*H*_2_), 2.32‐2.23 (m, 4H, α^Lau^−C*H*_2_), 1.76 (s, 3H, C*H*_3_, NHAc), 1.72‐1.52 (m, 8H, β^Lau^−C*H*_2_, γ^Myr^−C*H*_2_), 1.38–1.12 (m, 68H, C*H*_2_), 0.92–0.84 (m, 12H, ω^Myr^−C*H*_3_, ω^Lau^−C*H*_3_), 0.80 (s, 9H, Si(CH_3_)_2_C(C*H*_3_)_3_), −0.02 (s, 3H, Si(C*H*_3_)_2_C(CH_3_)_3_), −0.07 (s, 3H, Si(C*H*_3_)_2_C(CH_3_)_3_); ^13^C NMR (151 MHz, CDCl_3_): δ 174.22, 173.42 (C=O, Lau), 171.12 (C=O, NHAc), 170.28, 170.17 (C=O, Myr), 137.38 (C_q_, Ph), 135.72, 135.67 (C_q_, Ph), 129.10, 128.79, 128.74, 128.69, 128.48, 128.20, 128.15, 128.10, 126.41 (CH, Ph), 101.97 (CH, benzylidene acetal), 97.82 (CH, C‐1′), 96.68 (CH, C‐1), 82.43 (CH, C‐4′), 79.70 (CH, d, ^2^ *J*_C,P_=5.3 Hz, C‐3), 73.62 (CH, C‐5), 71.71 (CH, C‐3′), 71.08, 71.00 (β^Myr^−CH) 70.55 (CH, C‐4), 70.05 (CH_2_, d, ^2^ *J*_C,P_=5.7 Hz, OP(O)(O*C*H_2_Ph)_2_), 69.95 (CH_2_, d, ^2^ *J*_C,P_=5.5 Hz, OP(O)(O*C*H_2_Ph)_2_), 68.79 (CH_2_, C‐6′), 66.25 (CH, C‐5′), 63.94 (CH_2_, C‐6), 57.93 (CH, C‐2′), 56.33 (CH, C‐2), 41.45, 40.14 (α^Myr^−CH_2_), 34.88, 34.86, 34.77, 34.14, 32.05, 29.86, 29.83, 29.81, 29.79, 29.77, 29.75, 29.72, 29.69, 29.67, 29.62, 29.51, 29.49, 29.44, 29.39, 29.34 (CH_2,_ Myr, Lau), 25.88 (CH_3_, Si(CH_3_)_2_C(*C*H_3_)_3_), 25.52, 25.35, 25.32, 25.21 (CH_2,_ Myr, Lau), 23.44 (CH_3_, NH*Ac*), 22.81 (CH_2,_ Myr, Lau), 18.22 (C_q_, Si(CH_3_)_2_ *C*(CH_3_)_3_), 14.23 (ω^Myr^−CH_3_, ω^Lau^−CH_3_), −4.06, −4.70 (CH_3_, Si(*C*H_3_)_2_C(CH_3_)_3_); ^31^P NMR (243 MHz, CDCl_3_): δ −0.35; HRMS (ESI) *m/z* calcd. for [M+COOH]^−^ C_94_H_154_N_2_O_21_PSi 1706.0556, found 1706.0580.

**4,6‐*O*‐Benzylidene‐3‐*O***‐***tert*** **‐butyldimethylsilyl‐2‐deoxy‐2‐[(*R*)‐3‐(dodecanoyloxy)tetradecanoylamino]‐β‐D‐glucopyranosyl‐(1↔1)‐2‐acetamido‐3,4‐bis‐*O*‐[bis(benzyloxy)phosphoryl]‐2‐deoxy‐6‐*O*‐[(*R*)‐3‐(dodecanoyloxy)tetradecanoyl]‐β‐D‐glucopyranoside (28)** To a stirred solution of **24** (37 mg, 0,02 mmol) in dry DCM (2 mL) dibenzyl *N,N*‐diisopropylphosphoramidite (62 mg, 60 μL, 0.18 mmol) and a solution of 1*H*‐tetrazole in CH_3_CN (0.45 M, 12.5 mg, 0.18 mmol) were added under atmosphere of Ar. After 30 min the mixture was cooled to −78 °C and a solution of *m*CPBA (38 mg, 0.223 mmol) in dry DCM (2 mL) was added. After stirring for 0.5 h, the reaction was quenched by addition of solution of Et_3_N (10 eq, 30 μL) in DCM (1.5 mL) and the mixture was warmed to r.t., diluted with DCM (50 mL) and washed with sat. aq. NaHCO_3_ (20 mL) and brine (20 mL). The organic layer was dried over Na_2_SO4, filtered and concentrated. The residue was purified by column chromatography on silica gel (toluene ‐ EtOAc, 2 : 1→1 : 1) and by size‐exclusion chromatography on Sephadex LH‐20 (EtOAc) to afford **28** (39 mg, 92 %) as white amorphous solid. R_f_=0.45 (toluene/EtOAc, 1 : 1); [α]_D_^20^=−13.6 (c 1.2, CHCl_3_); ^1^H NMR (600 MHz, CDCl_3_): δ 7.47–7.42 (m, 2H, Ph), 7.37–7.15 (m, 23H, Ph), 6.43 (d, 1H, ^3^ *J*_NH′,2′_=8.5 Hz, N*H*′), 5.89 (d, 1H, ^3^ *J*_NH,2_=8.0 Hz, N*H*), 5.47 (s, 1H, OC*H*Ph), 5.28‐5.19 (m, 2H, β^Myr^−C*H*), 5.06‐4.86 (m, 11H, H‐1, H‐3, H‐1′, OP(O)(OC*H*_2_Ph)_2_), 4.60 (dd, 1H, ^2^ *J*_6a,6b_=12.1 Hz, ^3^ *J*_5,6b_=1.8 Hz, H‐6b), 4.38 (m, 1H, H‐4) 4.29 (dd, 1H, ^2^ *J*_6a′,6b′_=10.4 Hz, ^3^ *J*_5′,6b′_=4.1 Hz, H‐6b′), 4.20 (m, 1H, H‐3′), 4.13 (dd, 1H, ^2^ *J*_6a,6b_=12.2 Hz, ^3^ *J*_5,6a_=6.8 Hz, H‐6a), 3.72‐3.62 (m, 2H, H‐5, H‐6a′) 3.58‐3.45 (m, 2H, H‐2, H‐2′), 3.45‐3.38 (m, 2H, H‐4′, H‐5′) 2.65‐2.53 (m, 2H, α^Myr^−C*H*_2_), 2.50 (dd, 1H, ^2^ *J*=15.4 Hz, ^3^ *J*=6.1 Hz, α^Myr^−C*H*_2_), 2.42 (dd, 1H, ^2^ *J*=15.4 Hz, ^3^ *J*=6.5 Hz, α^Myr^−C*H*_2_), 2.32‐2.24 (m, 4H, α^Lau^−C*H*_2_), 1.81 (s, 3H, C*H*_3_, NHAc), 1.74‐1.54 (m, 8H, β^Lau^−C*H*_2_, γ^Myr^−C*H*_2_), 1.40‐1.10 (m, 68H, C*H*_2_), 0.90‐0.84 (m, 12H, ω^Myr^−C*H*_3_, ω^Lau^−C*H*_3_), 0.81 (s, 9H, Si(CH_3_)_2_C(C*H*_3_)_3_), −0.01 (s, 3H, Si(C*H*_3_)_2_C(CH_3_)_3_), −0.07 (s, 3H, Si(C*H*_3_)_2_C(CH_3_)_3_); ^13^C NMR (151 MHz, CDCl_3_): δ 173.85, 173.36 (C=O, Lau), 171.21 (C=O, NHAc), 170.26, 169.88 (C=O, Myr), 137.41 (C_q_, Ph), 135.92, 135.87, 135.78, 135.74, 135.71, 135.66, 135.64, 135.59 (C_q_, Ph), 129.10, 128.71, 128.68, 128.62, 128.60, 128.57, 128.36, 128.25, 128.21, 128.16, 126.43 (CH, Ph), 101.97 (CH, benzylidene acetal), 98.32 (CH, C‐1′), 97.22 (CH, C‐1), 82.46 (CH, C‐4′), 77.54 (CH, m, C‐3), 74.82 (CH, m, C‐4), 72.49 (CH, C‐5), 71.54 (CH, C‐3′), 70.83, 70.66 (β^Myr^−CH) 69.97, 69.94, 69.92, 69.86, 69.84, 69.82, 69.80 (CH_2_, OP(O)(O*C*H_2_Ph)_2_), 68.86 (CH_2_, C‐6′), 66.12 (CH, C‐5′), 63.04 (CH_2_, C‐6), 58.28 (CH, C‐2′), 56.52 (CH, C‐2), 41.22, 39.53 (α^Myr^−CH_2_), 34.89, 34.79, 34.69, 34.21, 32.07, 29.89, 29.83, 29.80, 29.79, 29.73, 29.72, 29.68, 29.54, 29.50, 29.42, 29.39 (CH_2,_ Myr, Lau), 25.91 (CH_3_, Si(CH_3_)_2_C(*C*H_3_)_3_), 25.46, 25.41, 25.29, 25.22 (CH_2,_ Myr, Lau), 23.60 (CH_3_, NH*Ac*), 22.83 (CH_2,_ Myr, Lau), 18.24 (C_q_, Si(CH_3_)_2_ *C*(CH_3_)_3_), 14.24 (ω^Myr^−CH_3_, ω^Lau^−CH_3_), −4.06, −4.63 (CH_3_, Si(*C*H_3_)_2_C(CH_3_)_3_); ^31^P NMR (243 MHz, CDCl_3_): δ −1.38, −1.67; HRMS (ESI) *m/z* calcd. for [M+COOH]^−^ C_108_H_167_N_2_O_24_P_2_Si 1966.1159, found 1966.1174.

**4,6‐*O*‐Benzylidene‐2‐deoxy‐2‐[(*R*)‐3‐(dodecanoyloxy)tetradecanoyl‐amino]‐β‐D‐glucopyranosyl‐(1↔1)‐2‐acetamido‐3,4‐bis‐*O*‐[bis(benzyloxy)phosphoryl]‐2‐deoxy‐6‐*O*‐[(*R*)‐3‐(dodecanoyloxy)tetradecanoyl]‐β‐D‐glucopyranoside (29)** To a stirred solution of **28** (35 mg, 0.018 mmol) in dry THF (2 mL) in a PTFE flask a solution of Et_3_N ⋅ 3HF (TREAT‐HF, 500 μL) was added under atmosphere of Ar. The reaction mixture was stirred for 24 h at r.t., diluted with EtOAc (50 mL), and washed with sat. aq. NaHCO_3_ (2×20 mL) and brine (20 mL). The organic layer was dried over Na_2_SO_4_, filtered and concentrated. The residue was purified by column chromatography on silica gel (toluene ‐ EtOAc, 1 : 1→0 : 1, v/v) and by size exclusion chromatography on Sephadex LH‐20 (EtOAc) to give **29** (37 mg, 86 %). R_f_=0.38 (EtOAc–toluene, 2 : 1); [α]_D_^20^=−10 (c 1,0, CHCl_3_); ^1^H NMR (600 MHz, CDCl_3_): δ 7.50–7.46 (m, 2H, Ph), 7.36–7.16 (m, 23H, Ph), 6.46 (d, 1H, ^3^ *J*_NH′,2′_=6.6 Hz, N*H*′), 5.99 (d, 1H, ^3^ *J*_NH,2_=8.0 Hz, N*H*), 5.53 (s, 1H, OC*H*Ph), 5.25‐5.17 (m, 2H, β^Myr^−C*H*), 5.03–4.89 (m, 10H, H‐1, H‐3, OP(O)(OC*H*_2_Ph)_2_), 4.88 (d, 1H, ^3^ *J*_1′,2′_=8.5 Hz, H‐1′), 4.55 (dd, 1H, ^2^ *J*_6a,6b_=12.2 Hz, ^3^ *J*_5,6b_=2.0 Hz, H‐6b), 4.40 (m, 1H, H‐4), 4.30 (dd, 1H, ^2^ *J*_6a′,6b′_=10.4 Hz, ^3^ *J*_5′,6b′_=5.0 Hz, H‐6b′), 4.23 (dd, 1H, ^2^ *J*_6a,6b_=12.0 Hz, ^3^ *J*_5,6a_=6.7 Hz, H‐6a), 4.19 (d, 1H, ^3^ *J*_OH,3′_=2.8 Hz, 3′‐O*H*), 4.12 (ddd, 1H, ^3^ *J*_2′,3′_=^3^ *J*_3′,4′_=9.4 Hz, ^3^ *J*_OH,3′_=2.9 Hz, H‐3′), 3.75‐3.68 (m, 2H, H‐5, H‐6a′) 3.65 (m, 1H, H‐2), 3.55 (dd, 1H, ^3^ *J*_3′,4′_=^3^ *J*_4′,5′_=9.2 Hz, H‐4′), 3.52 (m, 1H, H‐2′), 3.44 (ddd, 1H, ^3^ *J*_5′,6a′_=^3^ *J*_4′,5′_=9.8 Hz, ^3^ *J*_5′,6b′_=5.0 Hz, H‐5′), 2.60 (dd, 1H, ^2^ *J*=15.6 Hz, ^3^ *J*=7.5 Hz, α^Myr^−C*H*_2_), 2.55 (dd, 1H, ^2^ *J*=15.5 Hz, ^3^ *J*=5.1 Hz, α^Myr^−C*H*_2_), 2.51‐2.42 (m, 2H, α^Myr^−C*H*_2_), 2.31‐2.25 (m, 4H, α^Lau^−C*H*_2_), 1.83 (s, 3H, C*H*_3_, NHAc), 1.66‐1.54 (m, 8H, β^Lau^−C*H*_2_, γ^Myr^−C*H*_2_), 1.38‐1.16 (m, 68H, C*H*_2_), 0.91‐0.82 (m, 12H, ω^Myr^−C*H*_3_, ω^Lau^−C*H*_3_); ^13^C NMR (151 MHz, CDCl_3_): δ 174.26, 173.72 (C=O, Lau), 171.94 (C=O, Myr), 171.34 (C=O, NHAc), 170.06 (C=O, Myr), 137.25 (C_q_, Ph), 135.79, 135.74, 135.67, 135.63, 135.57, 135.53, 135.49 (C_q_, Ph), 129.21, 128.79, 128.73, 128.67, 128.65, 128.31, 128.27, 128.22, 128.17, 126.51 (CH, Ph), 102.03 (CH, benzylidene acetal), 98.29 (CH, C‐1′), 97.48 (CH, C‐1), 81.29 (CH, C‐4′), 77.50 (CH, m, C‐3), 74.58 (CH, m, C‐4), 72.74 (CH, br, C‐5), 71.58, 71.56 (CH, C‐3′, β^Myr^−CH), 70.54 (β^Myr^−CH) 70.03, 70.00, 69.95, 69.91 (CH_2_, OP(O)(O*C*H_2_Ph)_2_), 68.67 (CH_2_, C‐6′), 66.63 (CH, C‐5′), 62.98 (CH_2_, C‐6), 58.42 (CH, C‐2′), 56.12 (CH, C‐2), 42.44, 39.62 (α^Myr^−CH_2_), 34.88, 34.75, 34.69, 34.60, 32.06, 29.84, 29.81, 29.79, 29.77, 29.72, 29.70, 29.62, 29.60, 29.51, 29.49, 29.47, 29.42, 29.39, 29.37, 25.46, 25.39, 25.36, 25.15 (CH_2,_ Myr, Lau), 23.58 (CH_3_, NH*Ac*), 22.83 (CH_2,_ Myr, Lau), 14.24 (ω^Myr^−CH_3_, ω^Lau^−CH_3_); ^31^P NMR (243 MHz, CDCl_3_): δ −1.31, −1.64; HRMS (ESI) *m/z* calcd. for [M+COOH]^−^ C_102_H_153_N_2_O_24_P_2_ 1852.0294, found 1852.0311.

**4,6‐*O*‐Benzylidene‐2‐deoxy‐2‐[(*R*)‐3‐(dodecanoyloxy)tetradecanoylamino]‐3‐*O*‐[(*R*)‐3‐(tetradecanoyloxy)tetradecanoyl]‐β‐D‐gluco‐pyranosyl‐(1↔1)‐2‐acetamido‐3,4‐bis‐*O*‐[bis(benzyloxy)phosphoryl]‐2‐deoxy‐6‐*O*‐[(*R*)‐3‐(dodecanoyloxy)tetradecanoyl]‐β‐D‐glucopyranoside (30)** To a stirred solution of **29** (33 mg, 0,018 mmol) in dry DCM (1 mL) (*R*)‐3‐(tetradecanoyloxy)tetradecanoic acid **FA6** (12.5 mg, 0.027 mmol), DIC (3.5 mg, 0.027 mmol) and DMAP (0,2 mg, 0.002 mmol) were successively added at 0 °C. The stirring was continued for 30 min at 0° C, and for 12 h at r.t. The reaction mixture was diluted with DCM (50 mL) and washed with citric acid (0.25 M, 20 mL), sat. aq. NaHCO_3_ (2×20 mL) and brine (20 mL). The organic layer was dried over Na_2_SO_4_, filtered and concentrated. The residue was purified by HPLC (toluene ‐ EtOAc, 2 : 1→1 : 1, v/v) to give **30** as white solid (30 mg, 72 %). R_f_=0.43 (toluene ‐ EtOAc, 1 : 1); [α]_D_^20^=−8.4 (c 0.8, CHCl_3_); ^1^H NMR (600 MHz, CDCl_3_): δ 7.42–7.38 (m, 2H, Ph), 7.33–7.15 (m, 23H, Ph), 6.54 (d, 1H, ^3^ *J*_NH′,2′_=9.2 Hz, N*H*′), 6.05 (d, 1H, ^3^ *J*_NH,2_=7.7 Hz, N*H*), 5.47 (s, 1H, OC*H*Ph), 5.36 (dd, 1H, ^3^ *J*_2′,3′_=^3^ *J*_3′,4′_=9.9 Hz, H‐3′), 5.21 (m, 1H, β^Myr^−C*H*), 5.18–5.06 (m, 4H, H‐1, H‐3, β^Myr^−C*H*), 5.03–4.88 (m, 9H, H‐1′, OP(O)(OC*H*_2_Ph)_2_), 4.55 (dd, 1H, ^2^ *J*_6a,6b_=11.8 Hz, ^3^ *J*_5,6b_=1.4 Hz, H‐6b), 4.38‐4.30 (m, 2H, H‐4, H‐6b′), 4.15 (dd, 1H, ^2^ *J*_6a,6b_=12.2 Hz, ^3^ *J*_5,6a_=7.4 Hz, H‐6a), 3.92 (m, 1H, H‐2′), 3.75–3.69 (m, 2H, H‐5, H‐6a′) 3.64 (dd, 1H, ^3^ *J*_3′,4′_=^3^ *J*_4′,5′_=9.4 Hz, H‐4′), 3.48 (ddd, 1H, ^3^ *J*_5′,6a′_=^3^ *J*_4′,5′_=9.7 Hz, ^3^ *J*_5′,6b′_=5.1 Hz, H‐5′), 3.43 (m, 1H, H‐2), 2.64‐2.53 (m, 3H, α^Myr^−C*H*_2_), 2.53‐2.45 (m, 2H, α^Myr^−C*H*_2_), 2.35‐2.24 (m, 5H, α^Lau^−C*H*_2_, α^Myr^−C*H*_2_), 2.15‐2.09 (m, 2H, α^Myr^−C*H*_2_), 1.82 (s, 3H, C*H*_3_, NHAc), 1.64‐1.46 (m, 12H, β^Lau^−C*H*_2_, γ^Myr^−C*H*_2_), 1.37‐1.07 (m, 106H, C*H*_2_), 0.91‐0.84 (m, 18H, ω^Myr^−C*H*_3_, ω^Lau^−C*H*_3_); ^13^C NMR (151 MHz, CDCl_3_): δ 174.01, 173.55, 173.24 (C=O, Lau, Myr), 171.46 (C=O, NHAc), 170.29, 169.97, 169.72 (C=O, Myr), 137.09 (C_q_, Ph), 135.90, 135.85, 135.75, 135.72, 135.67, 135.60, 135.56 (C_q_, Ph), 129.15, 128.73, 128.70, 128.64, 128.62, 128.60, 128.27, 128.17, 126.28 (CH, Ph), 101.58 (CH, benzylidene acetal), 98.50 (CH, C‐1′), 96.52 (CH, C‐1), 78.97 (CH, C‐4′), 77.5 (CH, C‐3), 74.97 (CH, m, C‐4), 72.45 (CH, br, C‐5), 71.43 (CH, C‐3′), 70.94, 70.70, 70.08 (β^Myr^−CH) 70.01, 69.98, 69.94, 69.90, 69.86, 69.82 (CH_2_, OP(O)(O*C*H_2_Ph)_2_), 68.70 (CH_2_, C‐6′), 66.57 (CH, C‐5′), 63.38 (CH_2_, C‐6), 56.96 (CH, C‐2), 54.47 (CH, C‐2′), 41.53, 39.78, 39.21 (α^Myr^−CH_2_), 34.86, 34.69, 34.65, 34.47, 34.22, 33.97, 32.08, 29.92, 29.86, 29.82, 29.77, 29.71, 29.64, 29.60, 29.58, 29.54, 29.52, 29.44, 29.41, 29.31, 25.41, 25.38, 25.22, 25.17, 25.13 (CH_2,_ Myr, Lau), 23.62 (CH_3_, NH*Ac*), 22.84 (CH_2,_ Myr, Lau), 14.25 (ω^Myr^−CH_3_, ω^Lau^−CH_3_); ^31^P NMR (243 MHz, CDCl_3_): δ −1.65, −1.73; HRMS (ESI) *m/z* calcd. for [M+COOH]^−^ C_130_H_205_N_2_O_27_P_2_ 2288.4210, found 2288.4198.

**6‐*O*‐Benzyl‐2‐deoxy‐2‐[(*R*)‐3‐(dodecanoyloxy)tetradecanoylamino]‐3‐*O*‐[(*R*)‐3‐(tetradecanoyloxy)tetradecanoyl]‐β‐D‐glucopyranosyl‐(1↔1)‐2‐acetamido‐3,4‐bis‐*O*‐[bis(benzyloxy)phosphoryl]‐2‐deoxy‐6‐*O*‐[(*R*)‐3‐(dodecanoyloxy)tetradecanoyl]‐β‐D‐glucopyranoside (31)** A solution of benzylidene acetal **30** (17 mg, 0.007 mmol) in dry DCM (2 mL) was stirred with powdered activated molecular sieves 4 Å under atmosphere of Ar for 2 h at r.t. The mixture was cooled to −78 °C and Et_3_SiH (6 eq, 5.16 mg 0.044 mmol in 0.5 mL DCM) followed by TfOH (10 eq, 11 mg, 0.074 mmol in 0.5 mL DCM) were added under atmosphere of Ar. The reaction was stirred at −78 °C for 2 h, then Et_3_N (0.1 mmol) and MeOH (0.2 mL) were added, and the mixture was stirred for 10 min. The mixture was let to warm up to r.t., diluted with DCM (50 mL), filtered over a pad of Celite and washed successively with sat. aq. NaHCO_3_ (20 mL), water (20 mL) and brine (20 mL). The organic layer was dried over Na_2_SO_4_, filtered and concentrated. The residue was purified by column chromatography on silica gel (toluene‐EtOAc, 3 : 1→1 : 1) to give **31** as white solid (14 mg, 90 %). R_f_=0.22 (toluene ‐ EtOAce, 1 : 1); [α]_D_^20^=−7.0 (c 0.7, CHCl_3_); ^1^H NMR (600 MHz, CDCl_3_): δ 7.37–7.15 (m, 25H, Ph), 6.33 (d, 1H, ^3^ *J*_NH′,2′_=8.8 Hz, N*H*′), 5.95 (d, 1H, ^3^ *J*_NH,2_=8.1 Hz, N*H*), 5.20 (m, 1H, β^Myr^−C*H*), 5.16‐5.08 (m, 3H, H‐1, β^Myr^−C*H*), 5.07‐4.87 (m, 10H, H‐3, H‐3′, OP(O)(OC*H*_2_Ph)_2_), 4.79 (d, 1H, ^3^ *J*_1′,2′_=8.5 Hz, H‐1′), 4.59 (d, 1H, ^2^ *J*_A,B_=12.1 Hz, OC*H_2_* Ph), 4.56 (d, 1H, ^2^ *J*_A,B_=12.3 Hz, OC*H_2_* Ph), 4.52 (dd, 1H, ^2^ *J*_6a,6b_=12.1 Hz, ^3^ *J*_5,6b_=2.0 Hz, H‐6b), 4.34 (m, 1H, H‐4), 4.15 (dd, 1H, ^2^ *J*_6a,6b_=12.1 Hz, ^3^ *J*_5,6a_=7.3 Hz, H‐6a), 3.87 (m, 1H, H‐2′), 3.78 (dd, 1H, ^2^ *J*_6a′,6b′_=10.6 Hz, ^3^ *J*_5′,6b′_=3.2 Hz, H‐6b′), 3.73 (dd, 1H, ^2^ *J*_6a′,6b′_=10.8 Hz, ^3^ *J*_5′,6a′_=4.9 Hz, H‐6a′), 3.68 (ddd, 1H, ^3^ *J*_4,5_=9.6 Hz, ^3^ *J*_5,6a_=7.3 Hz, ^3^ *J*_5,6b_=2.1 Hz, H‐5), 3.63 (ddd, 1H, ^3^ *J*_3′,4′_=^3^ *J*_4′,5′_=9.3 Hz, ^3^ *J*_OH′,4′_=3.4 Hz, H‐4′), 3.51 (m, H‐5′), 3.47 (m, 1H, H‐2), 3.34 (d, 1H, ^3^ *J*_OH′,4′_=3.5 Hz, O*H*′), 2.62–2.52 (m, 3H, α^Myr^−C*H*_2_), 2.51–2.45 (m, 2H, α^Myr^−C*H*_2_), 2.33–2.23 (m, 7H, α^Lau^−C*H*_2_, α^Myr^−C*H*_2_), 1.76 (s, 3H, C*H*_3_, NHAc), 1.64–1.48 (m, 12H, β^Lau^−C*H*_2_, γ^Myr^−C*H*_2_), 1.37–1.15 (m, 106H, C*H*_2_), 0.91–0.83 (m, 18H, ω^Myr^−C*H*_3_, ω^Lau^−C*H*_3_); ^13^C NMR (151 MHz, CDCl_3_): *δ* 174.38, 173.87, 173.45 (C=O, Lau, Myr), 171.36 (C=O, NHAc, Myr), 170.10, 169.99 (C=O, Myr), 138.12 (C_q_, Ph), 135.91, 135.79, 135.74, 135.64, 135.59 (C_q_, Ph), 128.70, 128.61, 128.58, 128.55, 128.26, 128.18, 128.16, 127.90, 127.79 (CH, Ph), 98.12 (CH, C‐1′), 96.54 (CH, C‐1), 77.56 (CH, m, C‐3), 76.22 (CH, C‐3′), 75.17 (CH, C‐5′), 74.97 (CH, m, C‐4), 73.87 (CH_2_, O*C*H_2_Ph), 72.42 (CH, br, C‐5), 71.20, 70.99, 70.65 (β^Myr^−CH) 69.99, 69.95, 69.89, 69.83, 69.80, 69.76 (CH_2_, C‐6′, OP(O)(O*C*H_2_Ph)_2_), 69.91 (CH, C‐4′), 63.38 (CH_2_, C‐6), 56.70 (CH, C‐2), 53.40 (CH, C‐2′), 41.56, 40.25, 39.64 (α^Myr^−CH_2_), 34.89, 34.86, 34.68, 34.64, 34.58, 34.19, 32.08, 29.91, 29.88, 29.86, 29.83, 29.81, 29.77, 29.72, 29.68, 29.59, 29.54, 29.52, 29.45, 29.44, 29.41, 29.31, 25.43, 25.37, 25.29, 25.21, 25.10 (CH_2,_ Myr, Lau), 23.54 (CH_3_, NH*Ac*), 22.84 (CH_2,_ Myr, Lau), 14.25 (ω^Myr^−CH_3_, ω^Lau^−CH_3_); ^31^P NMR (243 MHz, CDCl_3_): *δ* −1.64, −1.73; HRMS (ESI) *m/z* calcd. for [M+COOH]^−^ C_130_H_207_N_2_O_27_P_2_ 2290.4367, found 2290.4360.

**6‐*O*‐Benzyl‐4‐*O*‐[bis(benzyloxy)phosphoryl]‐2‐deoxy‐2‐[(*R*)‐3‐(dodecanoyloxy)tetradecanoylamino]‐3‐*O*‐[(*R*)‐3‐(tetradecanoyloxy)tetradecanoyl]‐β‐D‐glucopyranosyl‐(1↔1)‐2‐acetamido‐3,4‐bis‐*O*‐[bis(benzyloxy)phosphoryl]‐2‐deoxy‐6‐O‐[(*R*)‐3‐(dodecanoyloxy)tetradecanoyl]‐β‐D‐glucopyranoside (32)** To a stirred solution of **31** (13 mg, 0.006 mmol) in dry DCM (3 mL) bis(benzyloxy)(diisopropylamino)‐phosphine (0.15 mmol, 50 μL) and a solution of 1*H*‐tetrazol in acetonitrile (0.45 M; 0.15 mmol, 65 μL) were added successively under atmosphere of Ar. The reaction mixture was stirred for 1 h, then cooled at −78 °C and a solution of *m*CPBA (0.42 mmol, 72 mg) was added. The reaction mixture was stirred for 1 h at −78 °C, then quenched by addition of Et_3_N (50 μL) in MeOH (0.2 mL) and warmed to r.t. The mixture was diluted with DCM (50 mL) and washed with sat. aq. NaHCO_3_ (20 mL), water (20 mL) and brine (20 mL). The organic layer was dried over Na_2_SO_4_, filtered and concentrated. The residue was purified by size‐exclusion chromatography on Sephadex LH‐20 (EtOAc) to give **32** (15 mg, 95 %). *R*_f_=0.59 (hexane ‐ acetone, 3 : 1); ^1^H NMR (600 MHz, CDCl_3_): *δ* 7.33–7.15 (m, 35H, Ph), 6.37 (d, 1H, ^3^ *J*_NH′,2′_=8.5 Hz, N*H*′), 5.98 (d, 1H, ^3^ *J*_NH,2_=7.9 Hz, N*H*), 5.49 (dd, 1H, ^3^ *J*=10.2 Hz, ^3^ *J*=9.0 Hz, H‐3′), 5.21 (m, 1H, β^Myr^−C*H*), 5.18–5.13 (m, 2H, β^Myr^−C*H*), 5.07 (d, 1H, ^3^ *J*_1′,2′_=8.1 Hz, H‐1′), 5.03–4.83 (m, 14H, H‐1, H‐3, OP(O)(OC*H*_2_Ph)_2_), 4.52–4.35 (m, 5H, H‐4, H‐4′, H‐6b, OC*H_2_* Ph), 4.17 (dd, 1H, ^2^ *J*_6a,6b_=12.2 Hz, ^3^ *J*_5,6a_=6.5 Hz, H‐6a), 3.77 (dd, 1H, ^2^ *J*_6a′,6b′_=11.0 Hz, ^3^ *J*_5′,6b′_=0.5 Hz, H‐6b′), 3.67‐3.56 (m, 5H, H‐2, H‐2′, H‐5, H‐5′, H‐6a′), 2.61 (dd, 1H, ^2^ *J*=15.7 Hz, ^3^ *J*=7.6 Hz, α^Myr^−C*H*_2_), 2.56 (dd, 1H, ^2^ *J*=15.7 Hz, ^3^ *J*=5.3 Hz, α^Myr^−C*H*_2_), 2.47‐2.38 (m, 3H, α^Myr^−C*H*_2_), 2.35–2.23 (m, 5H, α^Myr^−C*H*_2_/α^Lau^−C*H*_2_), 2.21–2.16 (m, 2H, α^Myr^−C*H*_2_/α^Lau^−C*H*_2_), 1.74 (s, 3H, C*H*_3_, NHAc), 1.63–1.43 (m, 12H, β^Lau^−C*H*_2_, γ^Myr^−C*H*_2_), 1.36‐1.12 (m, 106H, C*H*_2_), 0.90‐0.85 (m, 18H, ω^Myr^−C*H*_3_, ω^Lau^−C*H*_3_); ^13^C NMR (151 MHz, CDCl_3_): δ 173.66, 173.54, 173.33 (C=O, Lau, Myr), 171.35 (C=O, NHAc), 170.41, 170.06, 170.02 (C=O, Myr), 138.19 (C_q_, Ph), 135.96, 135.92, 135.79, 135.75, 135.65, 135.61 (C_q_, Ph), 128.68, 128.61, 128.57, 128.54, 128.27, 128.19, 128.15, 128.09, 127.80 (CH, Ph), 97.61 (CH, C‐1′), 97.35 (CH, C‐1), 77.72 (CH, m, C‐3), 74.78 (CH, m, C‐4), 74.18 (CH, C‐5′), 74.14 (CH, C‐4′), 73.60 (CH_2_, O*C*H_2_Ph), 72.56 (CH, C‐3′), 72.51 (CH, C‐5), 70.69, 70.48, 70.08 (β^Myr^−CH) 69.96, 69.93, 69.90, 69.80, 69.77, 69.73, 69.68, 69.64 (CH_2_, OP(O)(O*C*H_2_Ph)_2_), 68.72 (CH, C‐6′), 63.06 (CH_2_, C‐6), 56.20 (CH, C‐2), 55.10 (CH, C‐2′), 41.39, 39.95 (α^Myr^−CH_2_), 34.80, 34.65, 34.60, 34.49, 34.35, 32.09, 29.94, 29.88, 29.85, 29.83, 29.81, 29.79, 29.77, 29.75, 29.72, 29.66, 29.64, 29.57, 29.55, 29.48, 29.40, 29.39, 25.34, 25.25, 25.20 (CH_2,_ Myr, Lau), 23.47 (CH_3_, NH*Ac*), 22.84 (CH_2,_ Myr, Lau), 14.25 (ω^Myr^−CH_3_, ω^Lau^−CH_3_); ^31^P NMR (243 MHz, CDCl_3_): *δ* −1.51, −1.79, −1.98; HRMS (ESI) *m/z* calcd. for [M+COOH]^−^ C_144_H_220_N_2_O_30_P_3_ 2550.4969, found 2550.4931.

**2‐Deoxy‐2‐[(*R*)‐3‐(dodecanoyloxy)tetradecanoylamino]‐4‐*O*‐phosphoryl‐3‐*O*‐[(*R*)‐3‐(tetradecanoyloxy)tetradecanoyl]‐β‐D‐glucopyranosyl‐(1↔1)‐2‐acetamido‐2‐deoxy‐6‐*O*‐[(*R*)‐3‐(dodecanoyloxy)tetradecanoyl]‐3,4‐bis‐*O*‐phosphoryl‐β‐D‐glucopyranoside (*ββ*‐DLAM937)** A pressure reactor was charged with Pd‐black (8 mg) and a solution of **32** (4 mg, 1.6 μmol) in toluene ‐ MeOH (2 : 1, 1 mL) was added. The vessel was closed, purged with argon and then filled with hydrogen (8.5 bar) under stirring. The mixture was stirred for 72 h at r.t., then diluted with toluene–MeOH (2 : 1, 10 mL), filtered through a syringe filter (regenerated cellulose; 0.45 μm) and concentrated. The residue was purified by size exclusion chromatography on Bio‐Beads S−X1 support (toluene ‐ DCM ‐ MeOH, 5 : 3 : 1) which afforded ***β*** ***β*‐DLAM937** (2.8 mg, 1.5 μmol, 93 %). ^1^H NMR (600 MHz, MeOD−CDCl_3_, 3 : 2): δ 5.31 (dd, 1H, ^3^ *J*=^3^ *J*=9.8 Hz, H‐3′), 5.25 (m, 1H, β^Myr^−C*H*), 5.22–5.14 (m, 2H, β^Myr^−C*H*), 4.93–4.87 (m, 2H, H‐1, H‐1′), 4.45‐4.36 (m, 2H, H‐3, H‐6b), 4.26 (dd, 1H, ^2^ *J*_6a,6b_=12.1 Hz, ^3^ *J*_5,6a_=6.2 Hz, H‐6a), 4.20–4.12 (m, 2H, H‐4, H‐4′), 3.84‐3.79 (m, 2H, H‐6a′, H‐6b′), 3.71–3.59 (m, 3H, H‐2, H‐5, H‐2′), 3.46 (m, 1H, H‐5′), 2.72–2.54 (m, 4H, α^Myr^−C*H*_2_), 2.43 (dd, 1H, ^2^ *J*=14.9 Hz, ^3^ *J*=7.4 Hz, α^Myr^−C*H*_2_), 2.37–2.25 (m, 7H, α^Myr^−C*H*_2_, α^Lau^−C*H*_2_), 1.96 (s, 3H, C*H*_3_, NHAc), 1.67–1.50 (m, 12H, β^Lau^−C*H*_2_, γ^Myr^−C*H*_2_), 1.38–1.15 (m, 106H, C*H*_2_), 0.91‐0.82 (m, 18H, ω^Myr^−C*H*_3_, ω^Lau^−C*H*_3_); ^13^C NMR (151 MHz, MeOD / CDCl_3_ 3 : 2): δ 174.2–174.0 (C=O, m, Lau, Myr), 173.5 (C=O, NHAc), 170.6, 171.2, 171.7 (C=O, Myr), 98.1 (CH, C‐1, C‐1′), 78.0 (CH, C‐3), 76.1 (CH, C‐5′), 74.3 (CH, C‐4,), 73.5 (CH, C‐5, C‐3′), 72.6 (CH, C‐4′), 71.2, 71.1, 70.6 (β^Myr^−CH) 63.9 (CH_2_, C‐6), 61.3 (CH, C‐6′), 55.6 (CH, C‐2), 54.8 (CH, C‐2′), 41.3, 39.4 (α^Myr^−CH_2_), 35.2–34.3 (CH_2,_ m, Myr, Lau), 32.8–29.6 (CH_2,_ m, Myr, Lau), 25.8–25.1 (CH_2,_ m, Myr, Lau), 23.0 (CH_2,_ m, Myr, Lau), 22.8 (CH_3_, NH*Ac*), 14.2 (ω^Myr^−CH_3_, ω^Lau^−CH_3_); ^31^P NMR (243 MHz, MeOD/CDCl_3_ 3 : 2): δ 0.67, 0.38, −0.22; MALDI‐TOF MS: *m/z* calcd. for [M−H]^−^ C_94_H_176_N_2_O_28_P_3_ 1874.16, found 1874.18.

**4,6‐*O*‐Benzylidene‐3‐*O***‐***tert*** **‐butyldimethylsilyl‐2‐deoxy‐2‐(2,2,2‐trichloroethoxycarbonylamino)‐β‐D‐glucopyranosyl‐(1↔1)‐2‐acetamido‐6‐*O*‐allyloxycarbonyl‐3‐*O*‐[bis(benzyloxy)phosphoryl]‐2‐deoxy‐β‐D‐glucopyranoside (33)** To a stirred solution of **9** (221 mg, 216 μmol) in dry DCM (5 mL) *sym*−collidine (540 μL, 4 mmol) and allyl chloroformate (345 μL, 3.25 mmol) were added successively and the mixture was stirred for 12 h at r.t. under atmosphere of Ar. Then methanol (0.5 mL) was added, and the mixture was stirred for 1 h. The solvents were removed, and the residue was purified by MPLC (DCM–MeOH, 100:1→100 : 4) to give **33** (204 mg, 85 %). R_f_=0.22 (DCM ‐ methanol (2.5 %)); [α]_D_^20^=−17 (c 1.0, CHCl_3_); ^1^H NMR (600 MHz, CDCl_3_): δ 7.49–7.43 (m, 2H, Ph), 7.39–7.29 (m, 13H, Ph), 5.95 (m, 1H, CH_2_=C*H*‐, Alloc), 5.86 (d, 1H, ^3^ *J*_NH,2_=7.9 Hz, N*H*), 5.47 (s, 1H, OC*H*Ph), 5.38 (dd, 1H, ^3^ *J*=17.2 Hz, ^2^ *J*=1.4 Hz, −CH=C*H*_2 trans_, Alloc), 5.35 (d, 1H, ^3^ *J*_NH′,2′_=8.5 Hz, N*H*′), 5.29 (dd, 1H, ^3^ *J*=10.4 Hz, ^2^ *J*=1.0 Hz, −CH=C*H*_2 cis_, Alloc), 5.06 (d, 1H, ^3^ *J*_P,H_=8.2 Hz, OP(O)(OC*H*_2_Ph)_2_), 5.04‐4.98 (m, 3H, OP(O)(OC*H*_2_Ph)_2_, H‐1′), 4.94 (d, 1H, ^3^ *J*_1′,2′_=8.3 Hz, H‐1′), 4.84 (ddd, 1H, ^3^ *J*_2,3_=^3^ *J*_3,4_=^3^ *J*_P,3_=8.6 Hz, H‐3), 4.79 (d, 1H, ^2^ *J*=12.1 Hz, C*H*_2b_, Troc), 4.69–4.59 (m, 2H, OC(O)OC*H*_2_, Alloc, C*H*_2a_, Troc), 4.46 (m, 1H, H‐6b), 4.42–4.35 (m, 2H, H‐6a, O*H*), 4.24 (dd, 1H, ^2^ *J*_6a′,6b′_=10.5 Hz, ^3^ *J*_5′,6b′_=4.8 Hz, H‐6b′), 4.07 (dd, 1H, ^3^ *J*_2′,3′_=^3^ *J*_3′,4′_=8.9 Hz, H‐3′), 3.67 (dd, 1H, ^2^ *J*_6a′,6b′_=10.1 Hz, H‐6a′), 3.64–3.54 (m, 2H, H‐5, H‐4), 3.50‐3.37 (m, 3H, H‐2, H‐4′, H‐5′), 3.34 (ddd, 1H, ^3^ *J*_1′,2′_=^3^ *J*_2′,3′_=^3^ *J*_2′,NH′_=8.8 Hz, H‐2′), 1.77 (s, 3H, C*H*_3_, NHAc), 0.81 (s, 9H, Si(CH_3_)_2_C(C*H*_3_)_3_), 0.02 (s, 3H, Si(C*H*_3_)_2_C(CH_3_)_3_), −0.04 (s, 3H, Si(C*H*_3_)_2_C(CH_3_)_3_); ^13^C NMR (151 MHz, CDCl_3_): *δ* 170.86 (C=O, NH*Ac*), 155.26 (C=O, O*C*(O)OCH_2_, Alloc), 154.30 (C=O, Troc), 137.23 (C_q_, Ph), 135.65–135.47 (C_q_, m, Ph), 131.61 (CH, CH_2_=*C*H−, Alloc), 129.21, 128.95, 128.89, 128.83, 128.80, 128.29, 128.27, 128.23, 126.40 (CH, Ph), 119.37 (CH_2_, −CH=*C*H_2_, Alloc), 101.97 (CH, benzylidene acetal), 97.91 (CH, C‐1′), 96.95 (CH, C‐1), 95.63 (C_q_, Troc), 82.19 (CH, C‐4′), 79.97 (CH, m, C‐3), 74.79 (CH_2_, Troc), 73.75 (CH, C‐5), 71.75 (CH, C‐3′), 70.30‐70.18 (CH_2_, m, OP(O)(O*C*H_2_Ph)_2_), 70.05 (CH, C‐4), 68.92 (CH_2_, OC(O)O*C*H_2_, Alloc), 68.69 (CH_2_, C‐6′), 66.88 (CH_2_, C‐6), 66.31 (CH, C‐5′), 59.48 (CH, C‐2′), 56.08 (CH, C‐2), 25.85 (CH_3_, Si(CH_3_)_2_C(*C*H_3_)_3_), 23.46 (CH_3_, NH*Ac*), 18.27 (C_q_, Si(CH_3_)_2_ *C*(CH_3_)_3_), −4.07, −4.84 (CH_3_, Si(*C*H_3_)_2_C(CH_3_)_3_); ^31^P NMR (243 MHz, CDCl_3_): *δ* 0.02; HRMS (ESI) *m/z* calcd. for [M+COOH]^−^ C_49_H_63_Cl_3_N_2_O_19_PSi 1147.2603, found 1147.2591.

**4,6‐*O*‐Benzylidene‐3‐*O***‐***tert*** **‐butyldimethylsilyl‐2‐deoxy‐2‐(2,2,2‐trichloroethoxycarbonylamino)‐β‐D‐glucopyranosyl‐(1↔1)‐2‐acetamido‐6‐*O*‐allyloxycarbonyl‐3‐*O*‐[bis(benzyloxy)phosphoryl]‐2‐deoxy‐4‐*O*‐levulinoyl‐β‐D‐glucopyranoside (34)** To a stirred solution of **33** (244 mg, 221 μmol) in dry DCM (5 mL) levulinic acid (50 mg, 431 μmol), *N*‐(3‐dimethylaminopropyl)‐*N*′‐ethylcarbodiimide hydrochloride (EDC) (51 mg, 265 μmol), and DMAP (4.5 mg, 36 μmol) were successively added, and the mixture was stirred for 3 h at r.t. under atmosphere of Ar. Two more equal portions of acylation reagents [levulinic acid (50 mg, 431 μmol), EDC (51 mg, 265 μmol), and DMAP (4.5 mg, 36 μmol)] were added every 3 h under atmosphere of Ar and the stirring was continued for 6 h after the last addition. The mixture was diluted with EtOAc (200 mL) and washed with sat. aq. NaHCO_3_ (2×30 mL), water (30 mL) and brine (30 mL). The organic layer was dried over Na_2_SO_4_, filtered and concentrated. The residue was purified by column chromatography on silica gel (hexane ‐ EtOAc, 1 : 4→1 : 6) to afford **34** (220 mg, 183 μmol, 83 %). *R*_f_=0.28 (hexane ‐ EtOAc, 1 : 4); [α]_D_^20^=−22 (c 1.0, CHCl_3_); ^1^H NMR (600 MHz, CDCl_3_): δ 7.49–7.44 (m, 2H, Ph), 7.39–7.27 (m, 13H, Ph), 5.94 (m, 1H, CH_2_=C*H*−, Alloc), 5.82 (d, 1H, ^3^ *J*_NH,2_=7.9 Hz, N*H*), 5.49 (s, 1H, OC*H*Ph), 5.40–5.34 (m, 2H, N*H*′, −CH=C*H*_2 trans_, Alloc), 5.28 (m, 1H, −CH=C*H*_2 cis_, Alloc), 5.04 (dd, 1H, ^3^ *J*_3,4_=^3^ *J*_4,5_=9.6 Hz, H‐4), 5.00–4.89 (m, 6H, H‐1, H‐1′, OP(O)(OC*H*_2_Ph)_2_), 4.86 (m, 1H, H‐3), 4.76 (d, 1H, ^2^ *J*=11.9 Hz, C*H*_2b_, Troc), 4.68‐4.60 (m, 3H, C*H*_2a_, Troc, OC(O)OC*H*_2_, Alloc), 4.30 (dd, 1H, ^2^ *J*_6a′,6b′_=10.4 Hz, ^3^ *J*_5′,6b′_=4.7 Hz, H‐6b′), 4.29–4.19 (m, 2H, H‐6a, H‐6b), 4.13 (dd, 1H, ^3^ *J*_2′,3′_=^3^ *J*_3′,4′_=8.7 Hz, H‐3′), 3.75–3.66 (m, 3H, H‐5, H‐2, H‐6a′), 3.49‐3.38 (m, 2H, H‐4′, H‐5′), 3.30 (ddd, 1H, ^3^ *J*_1′,2′_=^3^ *J*_2′,3′_=^3^ *J*_2′,NH′_=8.7 Hz, H‐2′), 2.62‐2.54 (m, 1H, OC(O)CH_2_C*H*_*2*,_ Lev), 2.45–2.36 (m, 2H, OC(O)CH_2_C*H*_*2*,_ Lev, OC(O)C*H*_2,_ Lev), 2.27–2.20 (m, 1H, OC(O)C*H*_2,_ Lev), 2.07 (s, 3H, C*H*_3_, Lev), 1.84 (s, 3H, C*H*_3_, NHAc), 0.81 (s, 9H, Si(CH_3_)_2_C(C*H*_3_)_3_), 0.02 (s, 3H, Si(C*H*_3_)_2_C(CH_3_)_3_), −0.04 (s, 3H, Si(C*H*_3_)_2_C(CH_3_)_3_); ^13^C NMR (151 MHz, CDCl_3_): *δ* 206.31 (C=O, CH_2_ *C*(O)CH_3,_ Lev), 171.79 (C=O, O*C*(O)CH_2,_ Lev), 171.04 (C=O, NH*Ac*), 154.81 (C=O, Troc), 154.20 (C=O, O*C*(O)OCH_2_, Alloc), 137.27 (C_q_, Ph), 135.60 (C_q_, d, ^2^ *J*_*C,P*_=7.5 Hz, Ph), 131.65 (CH, CH_2_=*C*H−, Alloc), 129.20, 128.86, 128.80, 128.33, 128.29, 128.18, 126.40 (CH, Ph), 119.24 (CH_2_, −CH=*C*H_2_, Alloc), 101.96 (CH, benzylidene acetal), 98.09, 97.59 (CH, C‐1, C‐1′), 95.59 (C_q_, Troc), 82.21 (CH, C‐4′), 77.1 (CH, C‐3), 74.83 (CH_2_, Troc), 72.01 (CH, C‐5), 71.50 (CH, C‐3′), 69.98–69.81 (CH_2_, m, OP(O)(O*C*H_2_Ph)_2_), 69.44 (CH, d, ^3^ *J*_*C,P*_=3.2 Hz, C‐4), 68.95 (CH_2_, OC(O)O*C*H_2_, Alloc), 68.73 (CH_2_, C‐6′), 66.31 (CH, C‐5′), 66.15 (CH_2_, C‐6), 59.57 (CH, C‐2′), 55.95 (CH, C‐2), 37.83 (CH_2_, OC(O)CH_2_ *C*H_2,_ Lev), 29.75 (CH_3_, Lev), 27.88 (CH_2_, OC(O)*C*H_2,_ Lev), 25.86 (CH_3_, Si(CH_3_)_2_C(*C*H_3_)_3_), 23.56 (CH_3_, NH*Ac*), 18.28 (C_q_, Si(CH_3_)_2_ *C*(CH_3_)_3_), −4.08, −4.84 (CH_3_, Si(*C*H_3_)_2_C(CH_3_)_3_); ^31^P NMR (243 MHz, CDCl_3_): *δ* −1.39; HRMS (ESI) *m/z* calcd. for [M+COOH]^−^ C_54_H_69_Cl_3_N_2_O_21_PSi 1245.2971, found 1245.3004.

**4,6‐*O*‐Benzylidene‐2‐deoxy‐2‐(2,2,2‐trichloroethoxycarbonylamino)‐β‐D‐glucopyranosyl‐(1↔1)‐2‐acetamido‐6‐*O*‐allyloxycarbonyl‐3‐*O*‐[bis(benzyloxy)phosphoryl]‐2‐deoxy‐4‐*O*‐levulinoyl‐β‐D‐glucopyranoside (35)** To a stirred solution of **34** (136 mg, 113 μmol) in dry THF (2 mL) in a PTFE tube a solution of HF−Py (70 %; 500 μL, 20 mmol HF) was added under atmosphere of Ar and the stirring was continued for 6 h at r.t. The reaction mixture was diluted with EtOAc (100 mL) and washed with sat. aq. NaHCO_3_ (2×30 mL), water (30 mL) and brine (30 mL). The organic layer was dried over Na_2_SO_4_, filtered and concentrated. The residue was purified by column chromatography on silica gel ( EtOAc–toluene, 5 : 1→1:0) to give **35** (106 mg, 86 %). *R*_f_=0.38 (EtOAc ); [α]_D_^20^=−21 (c 1.0, CHCl_3_); ^1^H NMR (600 MHz, CDCl_3_): *δ* 7.51–7.46 (m, 2H, Ph), 7.38–7.27 (m, 13H, Ph), 6.01 (d, 1H, ^3^ *J*_NH,2_=8.3 Hz, N*H*), 5.93 (m, 1H, CH_2_=C*H*−, Alloc), 5.67 (br, N*H*′), 5.53 (s, 1H, OC*H*Ph), 5.37 (dd, 1H, ^3^ *J*=17.3 Hz, ^2^ *J*=1.4 Hz, −CH=C*H*_2 trans_, Alloc), 5.27 (dd, 1H, ^3^ *J*=10.4 Hz, ^2^ *J*=1.1 Hz, −CH=C*H*_2 cis_, Alloc), 5.05 (dd, 1H, ^3^ *J*_3,4_=^3^ *J*_4,5_=9.5 Hz, H‐4), 5.02 (d, 1H, ^3^ *J*_1,2_=8.2 Hz, H‐1), 5.00–4.84 (m, 6H, OP(O)(OC*H*_2_Ph)_2_, H‐1′, H‐3), 4.79 (d, 1H, ^2^ *J*=11.7 Hz, C*H*_2b_, Troc), 4.70 (d, 1H, ^2^ *J*=12.0 Hz, C*H*_2a_, Troc), 4.67–4.60 (m, 2H, OC(O)OC*H*_2_, Alloc), 4.32 (dd, 1H, ^2^ *J*_6a′,6b′_=10.4 Hz, ^3^ *J*_5′,6b′_=5.0 Hz, H‐6b′), 4.30–4.20 (m, 2H, H‐6a, H‐6b), 4.13 (m, 1H, H‐3′), 3.78‐3.69 (m, 3H, H‐5, H‐2, H‐6a′), 3.56 (dd, 1H, ^3^ *J*_3′,4′_=^3^ *J*_4′,5′_=9.2 Hz, H‐4′), 3.49‐3.38 (m, 2H, H‐2′, H‐5′), 2.62–2.55 (m, 1H, OC(O)CH_2_C*H*_*2*,_ Lev), 2.45‐2.35 (m, 2H, OC(O)CH_2_C*H*_*2*,_ Lev, OC(O)C*H*_2,_ Lev), 2.26–2.19 (m, 1H, OC(O)C*H*_2,_ Lev), 2.07 (s, 3H, C*H*_3_, Lev), 1.84 (s, 3H, C*H*_3_, NHAc), 1.72 (br, O*H*′); ^13^C NMR (151 MHz, CDCl_3_): δ 206.31 (C=O, CH_2_ *C*(O)CH_3,_ Lev), 171.80 (C=O, O*C*(O)CH_2,_ Lev), 171.21 (C=O, NH*Ac*), 154.79 (C=O, O*C*(O)OCH_2_, Alloc), 137.09 (C_q_, Ph), 135.54 (C_q_, d, ^2^ *J*_*C,P*_=8.1 Hz, Ph), 131.59 (CH, CH_2_=*C*H‐, Alloc), 129.40, 129.40, 128.90, 128.81, 128.46, 128.33, 128.18, 126.45 (CH, Ph), 119.33 (CH_2_, −CH=*C*H_2_, Alloc), 102.02 (CH, benzylidene acetal), 98.32 (CH, C‐1′), 97.73 (CH, C‐1), 95.65 (C_q_, Troc), 81.26 (CH, C‐4′), 76.86 (CH, m, C‐3), 74.78 (CH_2_, Troc), 72.09 (CH, C‐5), 71.28 (CH, C‐3′), 70.02‐69.87 (CH_2_, m, OP(O)(O*C*H_2_Ph)_2_), 69.42 (CH, d, ^3^ *J*_*C,P*_=3.4 Hz, C‐4), 68.98 (CH_2_, OC(O)O*C*H_2_, Alloc), 68.59 (CH_2_, C‐6′), 66.54 (CH, C‐5′), 66.10 (CH_2_, C‐6), 58.75 (CH, C‐2′), 55.84 (CH, C‐2), 37.81 (CH_2_, OC(O)CH_2_ *C*H_2,_ Lev), 29.74 (CH_3_, Lev), 27.86 (CH_2_, OC(O)*C*H_2,_ Lev), 23.54 (CH_3_, NH*Ac*); ^31^P NMR (243 MHz, CDCl_3_): *δ* −1.43; HRMS (ESI) *m/z* calcd. for [M+COOH]^−^ C_48_H_55_Cl_3_N_2_O_21_P 1131.2106, found 1131.2101.

**4,6‐*O*‐Benzylidene‐2‐deoxy‐2‐(2,2,2‐trichloroethoxycarbonylamino)‐3‐*O*‐[(*R*)‐3‐(tetradecanoyloxy)tetradecanoyl]‐β‐D‐glucopyranosyl‐(1↔1)‐2‐acetamido‐6‐*O*‐allyloxycarbonyl‐3‐*O*‐[bis(benzyloxy)phosphoryl]‐2‐deoxy‐4‐*O*‐levulinoyl‐β‐D‐glucopyranoside (36)** To a stirred solution of **35** (712 mg, 654 μmol) in dry DCM (4 mL) *N*‐(3‐dimethylaminopropyl)‐*N*′‐ethylcarbodiimide (EDC) (251 mg, 1.31 mmol), (*R*)‐3‐(tetradecanoyloxy)tetradecanoic acid **FA6** (446 mg, 981 μmol) and a solution of DMAP (4 mg/mL; 8.0 mg, 65 μmol) were added under atmosphere of Ar and the stirring was continued for 2 h. The reaction mixture was diluted with EtOAc (300 mL) and washed with sat. aq. NaHCO_3_ (2×50 mL), water (50 mL) and brine (50 mL). The organic layer was dried over Na_2_SO_4_, filtered and concentrated. The residue was purified by MPLC (hexane ‐ acetone, 2 : 1→3 : 2) to give **36** (801 mg, 80 %). *R*_f_=0.69 (hexane ‐ acetone, 1 : 1); [α]_D_^20^=−18 (c 1.0, CHCl_3_); ^1^H NMR (600 MHz, CDCl_3_): δ 7.44–7.39 (m, 2H, Ph), 7.37–7.28 (m, 13H, Ph), 5.96 (m, 1H, CH_2_=C*H*−, Alloc), 5.79 (d, 1H, ^3^ *J*_NH,2_=7.9 Hz, N*H* or N*H*′), 5.52 (d, 1H, ^3^ *J*_NH′,2′_=8.8 Hz, N*H* or N*H*′), 5.48 (s, 1H, OC*H*Ph), 5.42 (dd, 1H, ^3^ *J*_2′,3′_=^3^ *J*_3′,4′=_10.1 Hz, H‐3′), 5.39 (dd, 1H, ^3^ *J*=17.3 Hz, ^2^ *J*=1.3 Hz, −CH=C*H*_2 trans_, Alloc), 5.28 (dd, 1H, ^3^ *J*=10.4 Hz, ^2^ *J*=1.2 Hz, −CH=C*H*_2 cis_, Alloc), 5.17 (m, 1H, β^Myr^−C*H*), 5.07 (d, 1H, ^3^ *J*_1,2_=8.3 Hz, H‐1), 5.03 (dd, 1H, ^3^ *J*_3,4_=^3^ *J*_4,5=_9.5 Hz, H‐4), 5.00–4.90 (m, 6H, H‐1′, H‐3, OP(O)(OC*H*_2_Ph)_2_), 4.76 (d, 1H, ^2^ *J*=11.9 Hz, C*H*_2a_, Troc), 4.71 (d, 1H, ^2^ *J*=12.2 Hz, C*H*_2b_, Troc), 4.70‐4.62 (m, 2H, OC(O)OC*H*_2_, Alloc), 4.33 (dd, 1H, ^2^ *J*_6a′,6b′_=10.5 Hz, ^3^ *J*_5′,6b′=_5.0 Hz, H‐6b′), 4.29 (dd, 1H„ ^2^ *J*_6a,6b_=11.9 Hz, ^3^ *J*_5,6b_ =6.1 Hz, H‐6b), 4.20 (dd, 1H, ^2^ *J*_6a,6b_=12.2 Hz, ^3^ *J*_5,6a_=2.0 Hz, H‐6a), 3.76‐3.70 (m, 2H, H‐5, H‐6′a), 3.65 (dd, 1H, ^3^ *J*_3′,4′_=^3^ *J*_4′,5′=_9.5 Hz, H‐4′), 3.63‐3.56 (m, 2H, H‐2, H‐2′), 3.50 (ddd, 1H, ^3^ *J*_4′,5′_=^3^ *J*_5′,6′a=_9.7 Hz, ^3^ *J*_5′,6′b=_5.0 Hz, H‐5′), 2.63–2.55 (m, 2H, α^Myr^−C*H*_2_, OC(O)CH_2_C*H*_*2*,_ Lev), 2.51 (dd, 1H, ^2^ *J*=15.3 Hz, ^3^ *J*=5.5 Hz, α^Myr^−C*H*_2_), 2.45‐2.36 (m, 2H, OC(O)C*H*_2,_ Lev, OC(O)CH_2_C*H*_*2*,_ Lev), 2.22 (m, 1H, OC(O)C*H*_2,_ Lev), 2.16 (t, 2H, ^3^ *J*=7.5 Hz, α^Myr^−C*H*_2_), 2.08 (s, 3H, C*H*_3_, Lev), 1.84 (s, 3H, C*H*_3_, NHAc), 1.57–1.46 (m, 4H, β^Myr^−C*H*_2,_ γ^Myr^−C*H*_2_), 1.32–1.10 (m, 38H, C*H*_2_), 0.89‐0.86 (m, 6H, ω^Myr^−C*H*_3_); ^13^C NMR (151 MHz, CDCl_3_): *δ* 206.52 (C=O, CH_2_ *C*(O)CH_3,_ Lev), 173.48 (C=O, Myr), 171.77 (C=O, O*C*(O)CH_2,_ Lev), 171.30 (C=O, NH*Ac*), 169.76 (C=O, Myr), 154.77 (C=O, O*C*(O)OCH_2_, Alloc), 154.49 (C=O, Troc), 136.89 (C_q_, Ph), 135.46 (C_q_, Ph), 131.6 (CH, CH_2_=*C*H−, Alloc), 129.28, 129.14, 128.90, 128.88, 128.80, 128.35, 128.19, 128.15, 128.01, 126.25 (CH, Ph), 119.32 (CH_2_, −CH=*C*H_2_, Alloc), 101.57 (CH, benzylidene acetal), 98.75 (CH, C‐1′), 97.60 (CH, C‐1), 78.75 (CH, C‐4′), 76.88 (CH, C‐3), 74.53 (CH_2_, Troc), 71.93 (CH, C‐5), 70.91 (CH, C‐3′), 70.08 (β^Myr^−CH), 69.93 (CH_2_, OP(O)(O*C*H_2_Ph)_2_), 69.32 (CH, C‐4), 68.98 (CH_2_, OC(O)O*C*H_2_, Alloc), 68.56 (CH_2_, C‐6′), 66.55 (CH, C‐5′), 65.98 (CH_2_, C‐6), 56.62 (CH, C‐2 or C‐2′), 56.00 (CH, C‐2 or C‐2′), 39.28 (α^Myr^−CH_2_), 37.75 (CH_2_, OC(O)CH_2_ *C*H_2,_ Lev), 34.47 (α^Myr^−CH_2_), 33.91, 32.05, 29.84, 29.79, 29.78, 29.68, 29.67, 29.50, 29.45, 29.25, 22.83 (CH_2_, Myr), 27.81 (CH_2_, OC(O)*C*H_2,_ Lev), 25.18, 25.11 (β^Myr^−CH_2,_ γ^Myr^−CH_2_), 23.46 (CH_3_, NH*Ac*), 14.28 (ω^Myr^−CH_3_); ^31^P NMR (243 MHz, CDCl_3_): δ −1.46; HRMS (ESI) *m/z* calcd. for [M+H]^+^ C_75_H_107_Cl_3_N_2_O_22_P 1523.6113, found 1523.6111.

**4,6‐*O*‐Benzylidene‐2‐deoxy‐2‐[(*R*)‐3‐(dodecanoyloxy)tetradecanoylamino]‐3‐*O*‐[(*R*)‐3‐(tetradecanoyloxy)tetradecanoyl]‐β‐D‐glucopyranosyl‐(1↔1)‐2‐acetamido‐6‐*O*‐allyloxycarbonyl‐3‐*O*‐[bis(benzyloxy)phosphoryl]‐2‐deoxy‐4‐*O*‐levulinoyl‐β‐D‐glucopyranoside (37)** To a stirred solution of **36** (268 mg, 176 μmol) in acetic acid (20 mL) a freshly prepared zinc−copper couple (2.8 g) was added, and the mixture was vigorously stirred for 30 min. The mixture was diluted with EtOAc (100 mL), the solids were removed by filtration over a pad of Celite, the filtrate was washed with sat. aq. NaHCO_3_ (4×50 mL), water (50 mL) and brine (50 mL). The organic layer was dried over Na_2_SO_4_, filtered and concentrated. The residue was dried by repeated co‐evaporation with dry toluene (4×50 mL) and spitted into equal portions, 88 μmol each. Each portion was N‐acylated according to the following procedure. To a stirred solution of free amine (88 μmol) in dry DCM (1 mL) a solution of (*R)*‐3‐(dodecanoyloxy)tetradecanoic acid **FA5** in DCM (0.25 M; 49 mg, 116 μmol) was added followed by addition of EDC (*N*‐(3‐dimethylaminopropyl)‐*N*′‐ethylcarbodiimide) (33 mg, 173 μmol) in 5 equal portions over a period of 2 h. The stirring was continued for 12 h, the reaction was stopped by addition of DMAP (2 mg, 16 μmol) and MeOH (200 μL, 5 mmol). The mixture was stirred for 30 min, diluted with EtOAc (100 mL) and washed with sat. aq. NaHCO_3_ (2×20 mL), water (20 mL) and brine (20 mL). The organic layer was dried over Na_2_SO_4_, filtered and concentrated. The combined residues (176 μmol) were purified by MPLC (hexane ‐ acetone, 3 : 1→1 : 1) to give **37** (111 mg, 36 %). R_f_=0.51 (hexane ‐ acetone, 3 : 2); [α]_D_^20^=−14 (c 0.7, CH_2_Cl_2_); ^1^H NMR (600 MHz, CD_2_Cl_2_): δ 7.44‐7.40 (m, 2H, Ph), 7.39–7.30 (m, 13H, Ph), 6.02 (d, 1H, ^3^ *J*_NH′,2′_=8.6 Hz, N*H*′), 5.97 (m, 1H, CH_2_=C*H*−, Alloc), 5.89 (d, 1H, ^3^ *J*_NH,2_=8.7 Hz, N*H*), 5.49 (s, 1H, OC*H*Ph), 5.39 (dd, 1H, ^3^ *J*=17.2 Hz, ^2^ *J*=1.4 Hz, −CH=C*H*_2 trans_, Alloc), 5.33 (dd, 1H, ^3^ *J*_2′,3′_=^3^ *J*_3′,4′_=9.8 Hz, H‐3′), 5.29 (dd, 1H, ^3^ *J*=10.5 Hz, ^2^ *J*=1.3 Hz, −CH=C*H*_2 cis_, Alloc), 5.16–5.09 (m, 2H, β^Myr^−C*H*), 5.04–4.87 (m, 8H, H‐1, H‐4, 2xOP(O)(OC*H*_2_Ph)_2_, H‐1′, H‐3), 4.70–4.61 (m, 2H, OC(O)OC*H*_2_, Alloc), 4.34‐4.26 (m, 2H, H‐6b, H‐6b′), 4.20 (dd, 1H, ^2^ *J*_6a,6b_=12.0 Hz, ^3^ *J*_5,6a_=2.4 Hz, H‐6a), 3.83 (ddd, 1H, ^3^ *J*_2′,3′_=9.8 Hz, ^3^ *J*_1′,2′_=^3^ *J*_2′,NH′_=9.0 Hz, H‐2′), 3.77‐3.72 (m, 2H, H‐5, H‐6a′), 3.64 (dd, 1H, ^3^ *J*_3′,4′_=^3^ *J*_4′,5′_=9.6 Hz, H‐4′), 3.61 (ddd, 1H, ^3^ *J*_2,3_=9.8 Hz, ^3^ *J*_1,2_=^3^ *J*_2,NH_=8.7 Hz, H‐2), 3.51 (ddd, 1H, ^3^ *J*_4′,5′_=^3^ *J*_5′,6a′_=9.7 Hz, ^3^ *J*_5′,6b′_=5.1 Hz, H‐5′), 2.63‐2.56 (m, 2H, α^Myr^−C*H*_2_, OC(O)C*H*_2,_ Lev), 2.51 (dd, 1H, ^2^ *J*=15.5 Hz, ^3^ *J*=5.3 Hz, α^Myr^−C*H*_2_), 2.48‐2.38 (m, 3H, α^Myr^−C*H*_2_, OC(O)C*H*_2,_ Lev, OC(O)CH_2_C*H*_*2*,_ Lev), 2.36‐2.22 (m, 4H, α^Lau^−C*H*_2_, α^Myr^−C*H*_2_ OC(O)C*H*_2,_ Lev), 2.17–2.08 (m, 2H, α^Myr^−C*H*_2_), 2.06 (s, 3H, C*H*_3_, Lev), 1.82 (s, 3H, C*H*_3_, NHAc), 1.63–1.47 (m, 8H, β^Myr^−C*H*_2,_ β^Lau^−C*H*_2_, γ^Myr^−C*H*_2_), 1.34–1.13 (m, 72H, C*H*_2_), 0.91–0.85 (m, 12H, ω^Myr^−C*H*_3,_ ω^Lau^−C*H*_3_); ^13^C NMR (151 MHz, CD_2_Cl_2_): δ 206.67 (C=O, CH_2_ *C*(O)CH_3,_ Lev), 173.74 (C=O, Lau), 173.41 (C=O, Myr), 172.09 (C=O, O*C*(O)CH_2,_ Lev), 171.10 (C=O, NH*Ac*), 170.48 (C=O, Myr), 170.25 (C=O, Myr), 155.16 (C=O, O*C*(O)OCH_2_, Alloc), 137.62 (C_q_, Ph), 136.17 (C_q_, Ph), 132.19 (CH, CH_2_=*C*H−, Alloc), 129.46, 129.06, 129.05, 129.03, 128.57, 128.53, 128.42, 126.61 (CH, Ph), 119.09 (CH_2_, −CH=*C*H_2_, Alloc), 101.90 (CH, benzylidene acetal), 99.24 (CH, C‐1′), 97.99 (CH, C‐1), 79.18 (CH, C‐4′), 77.13 (CH, C‐3), 72.36 (CH, C‐5), 71.73 (CH, C‐3′), 71.05 (β^Myr^−CH), 70.29 (β^Myr^−CH), 70.04 (CH_2_, OP(O)(O*C*H_2_Ph)_2_), 69.64 (CH, C‐4), 69.20 (CH_2_, OC(O)O*C*H_2_, Alloc), 68.90 (CH_2_, C‐6′), 66.95 (CH, C‐5′), 66.30 (CH_2_, C‐6), 56.32 (CH, C‐2), 54.95 (CH, C‐2′), 41.73 (α^Myr^−CH_2_), 39.56 (α^Myr^−CH_2_), 38.01 (CH_2_, OC(O)CH_2_ *C*H_2,_ Lev), 34.89, 34.69, 34.53, 34.37, 32.37 (*C*H_2,_ Myr, Lau), 30.18, 30.14, 30.13, 30.10, 30.02, 29.99, 29.86, 29.83, 29.81, 29.78, 29.68, 29.56 (*C*H_2,_ Myr, Lau), 28.21 (CH_2_, OC(O)*C*H_2,_ Lev), 25.64, 25.48, 25.41 (CH_2_, Myr, Lau), 23.62 (CH_3_, NH*Ac*), 23.12 (CH_2_, Myr, Lau), 14.30 (ω^Myr^−CH_3_, ω^Lau^−CH_3_); ^31^P NMR (243 MHz, CD_2_Cl_2_): *δ* −1.45; HRMS (ESI) *m/z* calcd. for [M+COOH]^−^ C_99_H_154_N_2_O_25_P 1802.0584, found 1802.0597.

**4,6‐*O*‐Benzylidene‐2‐deoxy‐2‐[(*R*)‐3‐(dodecanoyloxy)tetradecanoyl‐amino]‐3‐*O*‐[(*R*)‐3‐(tetradecanoyloxy)tetradecanoyl]‐β‐D‐glucopyranosyl‐(1↔1)‐2‐acetamido‐6‐*O*‐allyloxycarbonyl‐3‐*O*‐[bis(benzyloxy)phosphoryl]‐2‐deoxy‐β‐D‐glucopyranoside (38)** To a stirred solution of **37** (77 mg, 45 μmol) a solution of hydrazine hydrate (55 %, 4.3 μL, 77 μmol) and acetic acid (0.4 mL, 7.1 mmol) in pyridine (1.7 mL, 21.4 mmol) were added at r.t. under atmosphere of Ar and the stirring was continued for 20 min. The mixture was diluted with EtOAc (100 mL) and washed with aq. HCl (20 mL, pH 5.5), sat. aq. NaHCO_3_ (2×20 mL), water (20 mL) and brine (20 mL). The organic layer was dried over Na_2_SO_4_, filtered and concentrated. The residue was purified by MPLC (DCM ‐ MeOH (0→2 %) to give **38** (62 mg, 84 %). *R*_f_=0.58 (EtOAc); [α]_D_^20^=−15 (c 1.0, CHCl_3_); ^1^H NMR (600 MHz, CD_2_Cl_2_): δ 7.43–7.30 (m, 13H, Ph), 6.19 (d, 1H, ^3^ *J*_NH,2_=8.3 Hz, N*H*), 6.16 (d, 1H, ^3^ *J*_NH′,2′_=9.1 Hz, N*H*′), 5.97 (m, 1H, CH_2_=C*H*−, Alloc), 5.48 (s, 1H, OC*H*Ph), 5.39 (m, 1H, −CH=C*H*_2 trans_, Alloc), 5.30–5.24 (m, 2H, −CH=C*H*_2 cis_, Alloc, H‐3′), 5.15–5.08 (m, 2H, β^Myr^−C*H*), 5.07–5.03 (m, 2H, OP(O)(OC*H*_2_Ph)_2_), 5.03–5.00 (m, 3H, OP(O)(OC*H*_2_Ph)_2_, H‐1), 4.89 (m, 1H, H‐3), 4.86 (d, 1H, ^3^ *J*_1′,2′_=8.2 Hz, H‐1′), 4.71–4.62 (m, 2H, OC(O)OC*H*_2_, Alloc), 4.57 (d, 1H, ^3^ *J*_*OH*,4_=4.3 Hz, O*H*) 4.44 (dd, 1H, ^2^ *J*_6a,6b_=11.9 Hz, ^3^ *J*_5,6b_=2.1 Hz, H‐6b), 4.39 (dd, 1H, ^2^ *J*_6a,6b_=11.8 Hz, ^3^ *J*_5,6a_=6.0 Hz, H‐6a), 4.26 (dd, 1H, ^2^ *J*_6a′,6b′_=10.4 Hz, ^3^ *J*_5′,6b′_=5.1 Hz, H‐6b′), 3.90 (ddd, 1H, ^3^ *J*_2′,3′_=9.9 Hz, ^3^ *J*_1′,2′_=^3^ *J*_2′,NH′_=9.1 Hz, H‐2′), 3.72 (dd, 1H, ^2^ *J*_6a′,6b′_=^3^ *J*_5′,6a′_=10.2 Hz, H‐6a′), 3.67 (dd, 1H, ^3^ *J*_3′,4′_=^3^ *J*_4′,5′_=9.4 Hz, H‐4′), 3.64 (m, H‐5), 3.57 (m, H‐4), 3.48 (ddd, 1H, ^3^ *J*_4′,5′_=^3^ *J*_5′,6a′_=9.7 Hz, ^3^ *J*_5′,6b′_=5.1 Hz, H‐5′), 3.42 (ddd, 1H, ^3^ *J*_2,3_=9.5 Hz, ^3^ *J*_1,2_=^3^ *J*_2,NH_=8.4 Hz, H‐2), 2.59 (dd, 1H, ^2^ *J*=15.5 Hz, ^3^ *J*=7.5 Hz, α^Myr^−C*H*_2_), 2.51 (dd, 1H, ^2^ *J*=15.5 Hz, ^3^ *J*=5.3 Hz, α^Myr^−C*H*_2_), 2.47 (dd, 1H, ^2^ *J*=14.8 Hz, ^3^ *J*=6.2 Hz, α^Myr^−C*H*_2_), 2.33 (dd, 1H, ^2^ *J*=14.9 Hz, ^3^ *J*=6.3 Hz, α^Myr^−C*H*_2_), 2.22 (t, 2H, ^3^ *J*=7.4 Hz, α^Lau^−C*H*_2_), 2.16‐2.07 (m, 2H, α^Myr^−C*H*_2_), 1.78 (s, 3H, C*H*_3_, NHAc), 1.62–1.45 (m, 8H, β^Myr^−C*H*_2,_ γ^Myr^−C*H*_2_, β^Lau^−C*H*_2_), 1.36‐1.10 (m, 72H, C*H*_2_), 0.90‐0.85 (m, 12H, ω^Myr^−C*H*_3_, ω^Lau^−C*H*_3_); ^13^C NMR (151 MHz, CD_2_Cl_2_): δ 173.78 (C=O, Lau), 173.39 (C=O, Myr), 171.07 (C=O, NH*Ac*), 170.51 (C=O, Myr), 170.31 (C=O, Myr), 155.50 (C=O, O*C*(O)OCH_2_, Alloc), 137.49 (C_q_, Ph), 135.97 (C_q_, Ph), 132.05 (CH, CH_2_=*C*H‐, Alloc), 129.43, 129.10, 128.96, 126.52 (CH, Ph), 119.19 (CH_2_, −CH=*C*H_2_, Alloc), 101.81 (CH, benzylidene acetal), 98.88 (CH, C‐1′), 97.21 (CH, C‐1), 80.16 (CH, C‐3), 79.01 (CH, C‐4′), 74.09 (CH, C‐5), 71.68 (CH, C‐3′), 71.06 (CH, β^Myr^−CH), 70.37 (CH_2_, OP(O)(O*C*H_2_Ph)_2_), 70.30 (CH, C‐4), 70.16 (CH, β^Myr^−CH), 69.13 (CH_2_, OC(O)O*C*H_2_, Alloc), 68.76 (CH_2_, C‐6′), 67.03 (CH_2_, C‐6), 66.91 (CH, C‐5′), 56.24 (CH, C‐2), 54.53 (CH, C‐2′), 41.59 (CH_2_, α^Myr^−CH_2_), 39.41 (CH_2_, α^Myr^−CH_2_), 34.48, 34.60, 34.38, 34.27, 32.32, 30.13, 30.08, 30.05, 29.99, 29.96, 29.94, 29.80, 29.76, 29.73, 29.59, 29.48, 25.58, 25.42, 25.34 (CH_2_, Myr, Lau), 23.51 (CH_3_, NH*Ac*), 23.08 (CH_2_, Myr, Lau), 14.28 (CH_3_, ω^Myr^−CH_3_, ω^Lau^−CH_3_); ^31^P NMR (243 MHz, CD_2_Cl_2_): *δ* 0.00; HRMS (ESI) *m/z* calcd. for [M+COOH]^−^ C_94_H_148_N_2_O_23_P 1704.0216, found 1704.0235.

**4,6‐*O*‐Benzylidene‐2‐deoxy‐2‐[(*R*)‐3‐(dodecanoyloxy)tetradecanoylamino]‐3‐*O*‐[(*R*)‐3‐(tetradecanoyloxy)tetradecanoyl]‐β‐D‐glucopyranosyl‐(1↔1)‐2‐acetamido‐6‐*O*‐allyloxycarbonyl‐3,4‐di‐*O*‐[bis(benzyloxy)phosphoryl]‐2‐deoxy‐β‐D‐glucopyranoside (39)** To a stirred solution of **38** (19 mg, 11.4 μmol) in dry DCM (1 mL) bis(benzyloxy)(diisopropylamino)phosphine (11.5 μL, 34 μmol) and a solution of 1*H*‐tetrazol in acetonitrile (0.45 M; 76 μL, 34 μmol) were successively added under atmosphere of Ar and the mixture was stirred for 20 min. Then a solution of *tert*‐butyl hydroperoxide in nonane (5.5 M; 3 μL, 16.4 μmol) was added and the mixture was stirred for 1.5 h. The mixture was diluted with EtOAc (100 mL) and washed with sat. aq. NaHCO_3_ (2×20 mL), aq. Na_2_S_2_O_3_ (5 %, 30 mL), water (20 mL) and brine (20 mL). The organic layer was dried over Na_2_SO_4_, filtered and concentrated. The residue was purified by MPLC (hexane ‐ acetone, 2 : 1) which afforded **39** (17 mg, 78 %). R_f_=0.27 (hexane ‐ acetone, 2 : 1); [α]_D_^20^=−15 (c 1.0, CH_2_Cl_2_); ^1^H NMR (600 MHz, CD_2_Cl_2_): δ 7.43–7.39 (m, 2H, Ph), 7.36–7.19 (m, 23H, Ph), 6.14 (d, 1H, ^3^ *J*_NH′,2′_=8.8 Hz, N*H*′), 6.09 (d, 1H, ^3^ *J*_NH,2_=8.5 Hz, N*H*), 5.96 (m, 1H, CH_2_=C*H*‐, Alloc), 5.48 (s, 1H, OC*H*Ph), 5.41–5.33 (m, 2H, −CH=C*H*_2 trans_, Alloc, H‐3′), 5.28 (m, 1H, −CH=C*H*_2 cis_, Alloc), 5.16–5.09 (m, 2H, β^Myr^−C*H*), 5.03–4.83 (m, 11H, OP(O)(OC*H*_2_Ph)_2_, H‐1, H‐1′, H‐3), 4.69–4.61 (m, 2H, OC(O)OC*H*_2_, Alloc), 4.54 (dd, 1H, ^2^ *J*_6a,6b_=12.2 Hz, ^3^ *J*_5,6b_=2.1 Hz, H‐6b), 4.45‐4.36 (m, 2H, H‐4, H‐6a), 4.31 (dd, 1H, ^2^ *J*_6a′,6b′_=10.5 Hz, ^3^ *J*_5′,6b′_=5.0 Hz, H‐6b′), 3.79 (ddd, 1H, ^3^ *J*_2′,3′_=10.1 Hz, ^3^ *J*_1′,2′_=^3^ *J*_2′,NH′_=8.6 Hz, H‐2′), 3.76‐3.64 (m, 4H, H‐2, H‐6a′, H‐5, H‐4′), 3.51 (ddd, 1H, ^3^ *J*_4′,5′_=^3^ *J*_5′,6′a_=9.7 Hz, ^3^ *J*_5′,6′b_=5.0 Hz, H‐5′), 2.59 (dd, 1H, ^2^ *J*=15.5 Hz, ^3^ *J*=7.6 Hz, α^Myr^−C*H*_2_), 2.51 (dd, 1H, ^2^ *J*=15.3 Hz, ^3^ *J*=5.4 Hz, α^Myr^−C*H*_2_), 2.46 (dd, 1H, ^2^ *J*=14.8 Hz, ^3^ *J*=6.5 Hz, α^Myr^−C*H*_2_), 2.35‐2.24 (m, 3H, ^2^ *J*=14.9 Hz, ^3^ *J*=6.3 Hz, α^Myr^−C*H*_2_, α^Lau^−C*H*_2_), 2.17‐2.07 (m, 2H, α^Myr^−C*H*_2_), 1.80 (s, 3H, C*H*_3_, NHAc), 1.62–1.45 (m, 8H, β^Myr^−C*H*_2,_ γ^Myr^−C*H*_2_, β^Lau^−C*H*_2_), 1.34‐1.10 (m, 72H, C*H*_2_), 0.90–0.84 (m, 12H, ω^Myr^−C*H*_3_, ω^Lau^−C*H*_3_); ^13^C NMR (151 MHz, CD_2_Cl_2_): δ 173.73 (C=O, Lau), 173.40 (C=O, Myr), 171.25 (C=O, NH*Ac*), 170.49 (C=O, Myr), 170.20 (C=O, Myr), 155.07 (C=O, O*C*(O)OCH_2_, Alloc), 137.62 (C_q_, Ph), 136.28, 136.07 (C_q_, Ph), 132.07 (CH, CH_2_=*C*H‐, Alloc), 129.41, 128.92, 128.90, 128.85, 128.82, 128.76, 128.52, 128.43, 128.42, 128.31, 126.53 (CH, Ph), 119.10 (CH_2_, −CH=*C*H_2_, Alloc), 101.80 (CH, benzylidene acetal), 98.89 (CH, C‐1′), 97.90 (CH, C‐1), 79.05 (CH, C‐4′), 77.88 (CH, C‐4), 74.28 (CH, C‐3), 72.87 (CH, C‐5), 71.52 (CH, C‐3′), 70.96 (CH, β^Myr^−CH), 70.28, 70.17, 70.04, 69.93 (CH_2_, OP(O)(O*C*H_2_Ph)_2_), 70.20 (CH, β^Myr^−CH), 69.11 (CH_2_, OC(O)O*C*H_2_, Alloc), 68.80 (CH_2_, C‐6′), 66.78 (CH, C‐5′), 66.33 (CH_2_, C‐6), 55.89 (CH, C‐2), 54.90 (CH, C‐2′), 41.59 (CH_2_, α^Myr^−CH_2_), 39.48 (CH_2_, α^Myr^−CH_2_), 34.81, 34.61, 34.39, 34.30, 32.32, 30.15, 30.09, 30.06, 29.99, 29.97, 29.94, 28.82, 29.79, 29.77, 29.74, 29.62, 29.49, 25.56, 25.43, 25.35 (CH_2_, Myr, Lau), 23.59 (CH_3_, NH*Ac*), 23.09 (CH_2_, Myr, Lau), 14.28 (CH_3_, ω^Myr^−CH_3_, ω^Lau^−CH_3_); ^31^P NMR (243 MHz, CD_2_Cl_2_): *δ* −1.34, −1.74; HRMS (ESI) *m/z* calcd. for [M+COOH]^−^ C_108_H_161_N_2_O_26_P_2_ 1964.0818, found 1964.0809.

**4,6‐*O*‐Benzylidene‐2‐deoxy‐2‐[(*R*)‐3‐(dodecanoyloxy)tetradecanoylamino]‐3‐*O*‐[(*R*)‐3‐(tetradecanoyloxy)tetradecanoyl]‐β‐D‐glucopyranosyl‐(1↔1)‐2‐acetamido‐3,4‐di‐*O*‐[bis(benzyloxy)phosphoryl]‐2‐deoxy‐β‐D‐glucopyranoside (40)** To a stirred solution of **39** (22 mg, 11.5 μmol) in dry DCM (1 mL) bis(triphenylphosphine)palladium(II) dichloride (1.2 mg, 1.7 μmol), water (1.2 μL, 69 μmol) and tributyltin hydride (SnHBu_3_, TBTH) (BHT‐stabilized; 4.0 μL, 15.0 μmol) were added and the mixture was stirred for 1.5 h. The solvents were removed, and the residue was purified by column chromatography on silica gel (1. DCM ‐ MeOH (2 %); 2. hexane ‐ acetone, 3 : 1→2 : 1) to give **40** (18.5 mg, 88 %). R_f_=0.41 (DCM ‐ MeOH, 97 : 3); [α]_D_^20^=−13.3 (c 1.0, CH_2_Cl_2_); ^1^H NMR (600 MHz, CD_2_Cl_2_): δ 7.44‐7.40 (m, 2H, Ph), 7.36‐7.18 (m, 23H, Ph), 6.14 (d, 1H, ^3^ *J*_NH′,2′_=8.3 Hz, N*H*′), 5.96 (d, 1H, ^3^ *J*_NH,2_=8.6 Hz, N*H*), 5.52.–5.47 (m, 2H, H‐3′, OC*H*Ph), 5.22 (d, 1H, ^3^ *J*_1′,2′_=8.3 Hz, H‐1′), 5.18‐5.10 (m, 2H, β^Myr^−C*H*), 5.05‐4.84 (m, 9H, OP(O)(OC*H*_2_Ph)_2_, H‐1), 4.77 (m, 1H, H‐3), 4.44 (m, H‐4), 4.31 (dd, 1H, ^2^ *J*_6a′,6b′_=10.3 Hz, ^3^ *J*_5′,6b′_=4.9 Hz, H‐6b′), 3.94 (m, 1H, O*H*), 3.83‐3.71 (m, 4H, H‐2, H‐6a, H‐6b, H‐6a′), 3.65 (dd, 1H, ^3^ *J*_3′,4′_=^3^ *J*_4′,5′_=9.5 Hz, H‐4′), 3.62‐3.53 (m, 2H, H‐2′, H‐5′), 3.42 (ddd, 1H, ^3^ *J*_4,5_=9.6 Hz, ^3^ *J*_5,6a_=^3^ *J*_5,6b_=2.8 Hz, H‐5), 2.58 (dd, 1H, ^2^ *J*=15.3 Hz, ^3^ *J*=7.4 Hz, α^Myr^−C*H*_2_), 2.52 (dd, 1H, ^2^ *J*=15.3 Hz, ^3^ *J*=5.2 Hz, α^Myr^−C*H*_2_), 2.47 (dd, 1H, ^2^ *J*=15.2 Hz, ^3^ *J*=7.0 Hz, α^Myr^−C*H*_2_), 2.38 (dd, 1H, ^2^ *J*=15.2 Hz, ^3^ *J*=5.5 Hz, α^Myr^−C*H*_2_), 2.36‐2.26 (m, 2H, α^Lau^−C*H*_2_), 2.20‐2.11 (m, 2H, α^Lau^−C*H*_2_) 1.84 (s, 3H, C*H*_3_, NHAc), 1.63‐1.48 (m, H, β^Myr^−C*H*_2_, γ^Myr^−C*H*_2_, β^Lau^−C*H*_2_), 1.34‐1.19 (m, 72H, C*H*_2_, Myr, Lau), 0.90‐0.85 (m, 12H, ω^Myr^−C*H*_3_, ω^Lau^−C*H*_3_); ^13^C NMR (151 MHz, CD_2_Cl_2_): δ 174.08 (C=O, Lau), 173.51 (C=O, Myr), 171.12 (C=O, NHAc or Myr), 171.05 (C=O, NHAc or Myr), 170.02 (C=O, Myr), 137.66 (C_q_, Ph), 136.36 (C_q_, d, ^2^ *J*_C,P_=7.5 Hz, Ph), 136.18‐136.05 (C_q_, m, Ph), 135.90 (C_q_, d, ^2^ *J*_C,P_=5.8 Hz, Ph), 129.45, 129.09, 128.98, 128.93, 128.89, 128.82, 128.56, 128.49, 128.47, 128.42, 128.29, 126.63 (CH, Ph), 101.93 (CH, benzylidene acetal), 98.79 (CH, C‐1), 98.57 (CH, C‐1′), 79.36 (CH, C‐4′), 78.20‐78.05 (CH, m, C‐3), 75.45 (CH, C‐5), 74.04‐73.88 (CH, m, C‐4), 71.38 (CH, C‐3′), 71.11 (CH, β^Myr^−CH), 70.54 (CH_2_, d, ^2^ *J*_C,P_=5.5 Hz, OP(O)(O*C*H_2_Ph)_2_), 70.43 (CH, β^Myr^−CH), 70.40 (CH_2_, OP(O)(O*C*H_2_Ph)_2_), 70.11 (CH_2_, d, ^2^ *J*_C,P_=5.5 Hz, OP(O)(O*C*H_2_Ph)_2_), 69.88 (CH_2_, d, ^2^ *J*_C,P_=5.5 Hz, OP(O)(O*C*H_2_Ph)_2_), 68.94 (CH_2_, C‐6′), 66.79 (CH, C‐5′), 61.01 (CH_2_, C‐6), 56.04 (CH, C‐2′), 55.91 (CH, C‐2), 41.90 (CH_2_, α^Myr^−CH_2_), 39.74 (CH_2_, α^Myr^−CH_2_), 34.89, 34.74, 34.69, 34.45, 32.37, 30.17, 30.15, 30.13, 30.02, 30.00, 29.86, 29.80, 29.78, 29.68, 29.57, 25.59, 25.50, 25.47, 25.44 (CH_2_, Myr, Lau), 23.68 (CH_3_, NH*Ac*), 23.12 (CH_2_, Myr, Lau), 14.30 (CH_3_, ω^Myr^−CH_3_, ω^Lau^−CH_3_); ^31^P NMR (243 MHz, CD_2_Cl_2_): δ −0.23, −0.78; HRMS (ESI) *m/z* calcd. for [M+COOH]^−^ C_104_H_157_N_2_O_24_P_2_ 1880.0607, found 1880.0604.

**4,6‐*O*‐Benzylidene‐2‐deoxy‐2‐[(*R*)‐3‐(dodecanoyloxy)tetradecanoylamino]‐3‐*O*‐[(*R*)‐3‐(tetradecanoyloxy)tetradecanoyl]‐β‐D‐glucopy‐ranosyl‐(1↔1)‐2‐acetamido‐3,4‐di‐*O*‐[bis(benzyloxy)phosphoryl]‐6‐*O*‐[(*R*)‐3‐(decanoyloxy)tetradecanoyl]‐2‐deoxy‐β‐D‐glucopyranoside (41)** To a stirred solution of triphenylphosphine (50 mg, 187 μmol) in dry THF (300 μL) diisopropyl azodicarboxylate (38 μL, 187 μmol) was added at 0 °C under atmosphere of Ar and the stirring was continued for 15 min. Then a solution of **40** (27 mg, 15 μmol) and (*R*)‐3‐(decanoyloxy)tetradecanoic acid **FA4** (45 mg, 112 μmol) in THF (300 μL) was added and the mixture was stirred for 20 h at r.t. The mixture was cooled to 0° C and triphenylphosphine (20 mg, 75 μmol), methanol (100 μL) and a solution of diisopropyl azodicarboxylate (15 μL, 75 μmol) in THF (40 μL) were added successively. The reaction mixture was stirred for 3 h, the solvent was removed, and the residue was purified by MPLC (1. hexane–EtOAc (34 %), column 2: hexane–acetone, 0→32 %) which afforded **41** (20.3 mg, 61 %). R_f_=0.51 (hexane ‐ acetone, 3 : 2); [α]_D_^20^=−10 (c 1.0, CH_2_Cl_2_); ^1^H NMR (600 MHz, CD_2_Cl_2_): δ 7.43‐7.37 (m, 2H, Ph), 7.36‐7.17 (m, 23H, Ph), 6.39 (d, 1H, ^3^ *J*_NH′,2′_=9.0 Hz, N*H*′), 5.90 (d, 1H, ^3^ *J*_NH,2_=8.3 Hz, N*H*), 5.49 (s, 1H, OC*H*Ph), 5.35 (dd, 1H, ^3^ *J*_2′,3′_=^3^ *J*_3′,4′_=9.9 Hz, H‐3′), 5.27–5.21 (m, 1H, β^Myr^−C*H*), 5.16–5.09 (m, 2H, β^Myr^−C*H*), 5.07‐5.87 (m, 11H, H‐1, OP(O)(OC*H*_2_Ph)_2_, H‐1′, H‐3), 4.56 (dd, 1H, ^2^ *J*_6a,6b_=12.0 Hz, ^3^ *J*_5,6b_=1.7 Hz, H‐6b), 4.39–4.29 (m, 2H, H‐4, H‐6b′), 4.14 (dd, 1H, ^2^ *J*_6a,6b_=12.1 Hz, ^3^ *J*_5,6a_=7.3 Hz, H‐6a), 3.88 (dd, 1H, ^3^ *J*_1′,2′_=^3^ *J*_2′,NH′_=^3^ *J*_2′,3′_=9.3 Hz, H‐2′), 3.77‐3.71 (m, 2H, H‐5, H‐6a′), 3.67 (dd, 1H, ^3^ *J*_3′,4′_=^3^ *J*_4′,5′_=9.5 Hz, H‐4′), 3.59 (ddd, 1H, ^3^ *J*_2,3_=9.6 Hz, ^3^ *J*_1,2_=^3^ *J*_2,NH_=8.8 Hz, H‐2), 3.50 (ddd, 1H, ^3^ *J*_4′,5′_=^3^ *J*_5′,6a′_=9.7 Hz, ^3^ *J*_5′,6b′_=5.0 Hz, H‐5′), 2.64–2.54 (m, 3H, α^Myr^−C*H*_2_), 2.53–2.43 (m, 2H, α^Myr^−C*H*_2_), 2.35–2.23 (m, 5H, α^Myr^−C*H*_2_, α^Lau^−C*H*_2_, α^Cap^−C*H*_2_), 2.18‐2.07 (m, 2H, α^Myr^−C*H*_2_), 1.81 (s, 3H, C*H*_3_, NHAc), 1.66‐1.44 (m, 12H, β^Myr^−C*H*_2_, γ^Myr^−C*H*_2_, β^Lau^−C*H*_2_, β^Cap^−C*H*_2_), 1.40‐1.10 (m, 102H, C*H*_2_, Myr, Lau, Cap), 0.92‐0.82 (m, 18H, ω^Myr^−C*H*_3_, ω^Lau^−C*H*_3_, ω^Cap^−C*H*_3_); ^13^C NMR (151 MHz, CD_2_Cl_2_): δ 174.14 (C=O, Cap), 173.58 (C=O, Lau), 173.31 (C=O, Myr), 171.20 (C=O, NHAc), 170.30, 170.25, 170.01 (C=O, Myr), 137.67 (C_q_, Ph), 136.23 (C_q_, Ph), 129.39, 128.99, 128.96, 128.90, 128.88, 128.81, 128.52, 128.46, 128.38, 126.59 (CH, Ph), 101.87 (CH, benzylidene acetal), 99.04 (CH, C‐1′), 97.52 (CH, C‐1), 79.29 (CH, C‐4′), 77.97 (CH, m, C‐3), 75.03 (CH, m, C‐4), 72.89 (CH, C‐5), 71.82 (CH, C‐3′), 71.03 (CH, β^Myr^−CH), 70.93 (CH, β^Myr^−CH), 70.25 (CH, β^Myr^−CH), 70.21–69.99 (m, CH_2_, OP(O)(O*C*H_2_Ph)_2_), 68.95 (CH_2_, C‐6′), 66.88 (CH, C‐5′), 63.67 (CH_2_, C‐6), 56.56 (CH, C‐2), 54.67 (CH, C‐2′), 41.68, 40.04, 39.49 (CH_2_, α^Myr^−CH_2_), 35.11, 34.92, 34.68, 34.51, 34.31, 32.38, 30.22, 30.17, 30.16, 30.14, 30.11, 30.05, 30.00, 29.94, 29.93, 29.89, 29.87, 29.85, 29.82, 29.72, 29.69, 29.58, 25.69, 25.53, 25.48, 25.42 (CH_2_, Myr, Lau, Cap), 23.68 (CH_3_, NH*Ac*), 23.13 (CH_2_, Myr, Lau, Cap), 14.30 (CH_3_, ω^Myr^−CH_3_, ω^Lau^−CH_3,_ ω^Cap^−CH_3_); ^31^P NMR (243 MHz, CD_2_Cl_2_): δ −1.46, −1.70; HRMS (ESI) *m/z* calcd. for [M+COOH]^−^ C_128_H_201_N_2_O_27_P_2_ 2260.3897, found 2260.3818.

**6‐*O*‐Benzyl‐2‐deoxy‐2‐[(*R*)‐3‐(dodecanoyloxy)tetradecanoylamino]‐3‐*O*‐[(*R*)‐3‐(tetradecanoyloxy)tetradecanoyl]‐β‐D‐glucopyranosyl‐(1↔1)‐2‐acetamido‐3,4‐di‐*O*‐[bis(benzyloxy)phosphoryl]‐6‐*O*‐[(*R*)‐3‐(decanoyloxy)tetradecanoyl]‐2‐deoxy‐β‐D‐glucopyranoside (42)** A solution of benzylidene acetal **41** (13 mg, 6.0 μmol) in dry DCM (4 mL) was stirred with powdered activated molecular sieves 4 Å under atmosphere of Ar for 2 h at r.t. The mixture was cooled to −78 °C and a solution of Et_3_SiH in DCM (1 M; 54 μL, 54 μmol) followed by a solution of TfOH in DCM (1 M, 108 μL, 108 μmol) were added successively under atmosphere of Ar. The stiring was continued for 2.5 h at −78 °C, then triethylamine (38 μL, 270 μmol) and methanol (146 μL, 3.6 mmol) were added successively and the mixture was stirred for 15 min. The mixture was warmed to r.t., the solids were removed by filtration over a pad of Celite, and the mixture was concentrated. The residue was purified by MPLC (toluene–EtOAc (20→100 %) followed by HPLC (YMC NP 10x250 mm; DCM ‐ MeOH (5 %)) which afforded **42** (10 mg, 74 %). R_f_=0.33 (EtOAc‐ toluene, 3 : 2); [α]_D_^20^=−8.0 (c 0.2, CH_2_Cl_2_); ^1^H NMR (600 MHz, CD_2_Cl_2_): δ 7.39–7.17 (m, 25H, Ph), 6.24 (d, 1H, ^3^ *J*_NH′,2′_=8.9 Hz, N*H*′), 5.88 (d, 1H, ^3^ *J*_NH,2_=7.9 Hz, N*H*), 5.21 (m, 1H, β^Myr^−C*H*), 5.14–4.86 (m, 13H, β^Myr^−C*H*, H‐1, OP(O)(OC*H*_2_Ph)_2_, H‐3′, H‐3), 4.79 (d, 1H, ^3^ *J*_1′,2′_=8.5 Hz, H‐1′), 4.59 (d, 1H, ^2^ *J*_A,B_=11.8 Hz, OC*H_2_* Ph), 4.55 (d, 1H, ^2^ *J*_A,B_=11.9 Hz, OC*H_2_* Ph), 4.53 (dd, 1H, ^2^ *J*_6a,6b_=12.2 Hz, ^3^ *J*_5,6b_=1.8 Hz, H‐6b), 4.34 (ddd, 1H, ^3^ *J*_3,4_=^3^ *J*_4,5_=^3^ *J*_P,4_=9.4 Hz, H‐4), 4.14 (dd, 1H, ^2^ *J*_6a,6b_=12.2 Hz, ^3^ *J*_5,6a_=7.3 Hz, H‐6a), 3.85 (ddd, 1H, ^3^ *J*_1′,2′_=^3^ *J*_2′,3′_=9.7 Hz, ^3^ *J*_2′,NH′_=9.2 Hz, H‐2′), 3.80‐3.69 (m, 3H, H‐6a′, H‐6b′, H‐5), 3.63 (ddd, 1H, ^3^ *J*_3′,4′_=^3^ *J*_4′,5′_=9.2 Hz, ^3^ *J*_4′,OH′_=3.5 Hz,H‐4′), 3.58‐3.49 (m, 2H, H‐2, H‐5′), 3.25 (d, 1H, ^3^ *J*_4′,OH′_=3.4 Hz, O*H*′), 2.62‐2.52 (m, 3H, α^Myr^−C*H*_2_), 2.51‐2.44 (m, 2H, α^Myr^−C*H*_2_), 2.33‐2.22 (m, 7H, α^Myr^−C*H*_2_, α^Lau^−C*H*_2_, α^Cap^−C*H*_2_), 1.76 (s, 3H, C*H*_3_, NHAc), 1.66‐1.47 (m, 12H, β^Myr^−C*H*_2,_ γ^Myr^−C*H*_2_, β^Lau^−C*H*_2_, β^Cap^−C*H*_2,_), 1.39‐1.12 (m, 102H, C*H*_2_, Myr, Lau, Cap), 0.91‐0.84 (m, 18H, ω^Myr^−C*H*_3_, ω^Lau^−C*H*_3_, ω^Cap^−C*H*_3_); ^13^C NMR (151 MHz, CD_2_Cl_2_): δ 174.63 (C=O, Myr or Lau), 174.05 (C=O, Cap), 173.52 (C=O, Myr or Lau), 171.55 (C=O, NHAc), 171.27 (C=O, Myr), 170.32 (C=O, Myr), 170.10 (C=O, Myr), 138.71 (C_q_, Ph), 136.51‐136.04 (C_q_, m, Ph), 128.97, 128.95, 128.89, 128.87, 128.81, 128.78, 128.50, 128.44, 128.38, 128.07 (CH, Ph), 98.67 (CH, C‐1′), 97.30 (CH, C‐1), 78.00 (CH, m, C‐3), 76.55 (CH, C‐3′), 75.61 (CH, C‐5′), 75.07 (CH, m, C‐4), 74.06 (CH_2_, O*C*H_2_Ph), 72.79 (CH, C‐5), 71.46 (CH, β^Myr^−CH), 71.13 (CH, β^Myr^−CH), 70.91 (CH, β^Myr^−CH), 70.26‐69.91 (m, CH_2_, OP(O)(O*C*H_2_Ph)_2_, C‐6′), 70.02 (CH, C‐4′), 63.72 (CH_2_, C‐6), 56.63 (CH, C‐2), 53.5 (CH, C‐2′), 41.72, 40.54, 39.97 (CH_2_, α^Myr^−CH_2_), 35.16, 35.10, 34.91, 34.87, 34.44, 32.39, 32.37, 30.21, 30.15, 30.12, 30.10, 30.07, 30.04, 30.00, 29.97, 29.88, 29.85, 29.83, 29.74, 29.70, 29.68, 29.58, 25.74, 25.67, 25.58, 25.52, 25.38 (CH_2_, Myr, Lau, Cap), 23.64 (CH_3_, NH*Ac*), 23.13 (CH_2_, Myr, Lau, Cap), 14.30 (CH_3_, ω^Myr^−CH_3_, ω^Lau^−CH_3,_ ω^Cap^−CH_3_); ^31^P NMR (243 MHz, CD_2_Cl_2_): δ −1.38, −1.69; HRMS (ESI) *m/z* calcd. for [M+COOH]^−^ C_128_H_203_N_2_O_27_P_2_ 2262.4054, found 2262.3982.

**6‐*O*‐Benzyl‐4‐*O*‐[bis(benzyloxy)phosphoryl]‐2‐deoxy‐2‐[(*R*)‐3‐(dodecanoyloxy)tetradecanoylamino]‐3‐*O*‐[(*R*)‐3‐(tetradecanoyloxy)tetradecanoyl]‐β‐D‐glucopyranosyl‐(1↔1)‐2‐acetamido‐3,4‐di‐*O*‐[bis(benzyloxy)phosphoryl]‐6‐*O*‐[(*R*)‐3‐(decanoyloxy)tetradecanoyl]‐2‐deoxy‐β‐D‐glucopyranoside (43)** To a stirred solution of **42** (6 mg, 2.5 μmol) in dry DCM (1 mL) bis(benzyloxy)(diisopropylamino)phosphine (5.0 μL, 15.2 μmol) and a solution of 1*H*‐tetrazol in acetonitrile (0.45 M; 23 μL, 10 μmol) were added successively under atmosphere of Ar and the reaction mixture was stirred for 30 min. The mixture was cooled to 0 °C and a solution of *m*−CPBA in DCM (44 mg/mL, 100 μL, 2.2 mg, 13 μmol) was added. The mixture was stirred for 2 h at 0° C, then acetone (100 μL) and triethylamine (7 μL, 50 μmol) were added successively and the mixture was stirred at 0 °C for 10 min, diluted with toluene (5 mL) and warmed up to rt. The solvents were removed, and the residue was purified by MPLC (DCM – MeOH (0→5 %)) and by size‐exclusion chromatography on Bio‐Beads S−X1 support (toluene–DCM, 2.5 : 1) which afforded **43** (6.3 mg, 99 %). R_f_=0.44 (EtOAc ‐ toluene, 2 : 1); [α]_D_^20^=−0.7 (c 1.0, CH_2_Cl_2_); ^1^H NMR (600 MHz, CD_2_Cl_2_): δ 7.36‐7.17 (m, 33H, Ph), 6.29 (d, 1H, ^3^ *J*_NH′,2′_=8.3 Hz, N*H*′), 5.86 (d, 1H, ^3^ *J*_NH,2_=8.2 Hz, N*H*), 5.40 (dd, 1H, ^3^ *J*=10.2 Hz, ^3^ *J*=9.1 Hz, H‐3′), 5.22 (m, 1H, β^Myr^−C*H*), 5.17‐5.11 (m, 2H, β^Myr^−C*H*), 5.05‐4.86 (m, 15H, H‐1′, H‐1, OP(O)(OC*H*_2_Ph)_2_, H‐3), 4.54‐4.42 (m, 4H, H‐6b, OC*H*_2_Ph, H‐4′), 4.37 (ddd, 1H, ^3^ *J*_3,4_=^3^ *J*_4,5_=^3^ *J*_P,4_=9.8 Hz, H‐4), 4.16 (dd, 1H, ^3^ *J*_6a,6b_=12.3 Hz, ^3^ *J*_5,6a_=6.6 Hz, H‐6a), 3.78 (m, 1H, H‐6b′), 3.72‐3.62 (m, 4H, H‐5, H‐2′, H‐5′, H‐6a′), 3.59 (m, 1H, H‐2), 2.64‐2.54 (m, 2H, α^Myr^−C*H*_2_), 2.49‐2.37 (m, 3H, α^Myr^−C*H*_2_), 2.34‐2.22 (m, 5H, α^Myr^−C*H*_2_, α^Cap^−C*H*_2_, α^Myr/Lau^−C*H*_2_), 2.22‐2.15 (m, 2H, α^Myr/Lau^−C*H*_2_), 1.74 (s, 3H, C*H*_3_, NHAc), 1.64‐1.44 (m, 12H, β^Myr^−C*H*_2,_ γ^Myr^−C*H*_2_, β^Lau^−C*H*_2_, β^Cap^−C*H*_2,_), 1.39‐1.18 (m, 102H, C*H*_2_, Myr, Lau, Cap), 0.92‐0.83 (m, 18H, ω^Myr^−C*H*_3_, ω^Lau^−C*H*_3_, ω^Cap^−C*H*_3_); ^13^C NMR (151 MHz, CD_2_Cl_2_): δ 173.88 (C=O, Cap), 173.58, 173.47 (C=O, Myr, Lau), 171.27 (C=O, NHAc), 170.43‐170.34 (C=O, m, Myr), 138.76 (C_q_, Ph), 136.53‐136.10 (C_q_, m, Ph), 128.95, 128.88, 128.87, 128.84, 128.77, 128.74, 128.51, 128.44, 128.38, 128.35, 128.11, 127.98 (CH, Ph), 98.16 (CH, C‐1′), 97.71 (CH, C‐1), 78.03 (CH, m, C‐3), 74.99 (CH, m, C‐4), 74.66 (CH, d, ^3^ *J*_C,P_=5.7 Hz, C‐5′), 74.21 (CH, d, ^2^ *J*_C,P_=5.8 Hz, C‐4′), 73.88 (CH_2_, O*C*H_2_Ph), 73.09 (CH, C‐3′), 72.80 (CH, m, C‐5), 70.85 (CH, β^Myr^−CH), 70.78 (CH, β^Myr^−CH), 70.21 (CH, β^Myr^−CH), 70.20‐69.86 (CH_2_, m, OP(O)(O*C*H_2_Ph)_2_), 68.96 (CH, C‐6′), 63.48 (CH_2_, C‐6), 56.49 (CH, C‐2), 54.89 (CH, C‐2′), 41.57, 39.77, 39.53 (CH_2_, α^Myr^−CH_2_), 35.07, 34.91, 34.83, 34.79, 34.71, 34.64, 32.29, 32.37, 30.24, 30.20, 30.17, 30.12, 30.09, 30.03, 30.00, 29.99, 29.95, 29.92, 29.84, 29.79, 29.75, 29.68, 29.65, 25.66, 25.63, 25.56, 25.53, 25.48 (CH_2_, Myr, Lau, Cap), 23.62 (CH_3_, NH*Ac*), 23.13 (CH_2_, Myr, Lau, Cap), 14.30 (CH_3_, ω^Myr^−CH_3_, ω^Lau^−CH_3,_ ω^Cap^−CH_3_); ^31^P NMR (243 MHz, CD_2_Cl_2_): δ −1.32, −1.72, −2.01; HRMS (ESI) *m/z* calcd. for [M+COOH]^−^ C_142_H_216_N_2_O_30_P_3_ 2522.4656, found 2522.4606.

**2‐Deoxy‐2‐[(*R*)‐3‐(dodecanoyloxy)tetradecanoylamino]‐4‐*O*‐phosphoryl‐3‐*O*‐[(*R*)‐3‐(tetradecanoyloxy)tetradecanoyl]‐β‐D‐glucopyranosyl‐(1↔1)‐2‐acetamido‐2‐deoxy‐6‐*O*‐[(*R*)‐3‐(decanoyloxy)tetradecanoyl]‐3,4‐bis‐*O*‐phosphoryl‐β‐D‐glucopyranoside (*ββ*‐DLAM3370)** To a stirred solution of **43** (12 mg, 4.8 μmol) in dry toluene ‐ MeOH (2 : 1, 18 mL) in a pressure reactor Pd black (45 mg) was added. The vessel was closed, purged with Ar, and then filled with hydrogen (8.5 bar). The mixture was stirred for 48 h at r.t., filtered through a syringe filter (regenerated cellulose; 0.45 μm), and the solvent was removed. The residue was purified by size exclusion chromatography on Bio‐Beads S−X1 Support; toluene ‐ DCM – MeOH, 5 : 3 : 1) which afforded ***β*** ***β*‐DLAM3370** (9 mg, 98 %). ^1^H NMR (600 MHz, MeOD−CDCl_3_ 3 : 2): δ 5.31 (dd, 1H, ^3^ *J*_2′,3′_=^3^ *J*_3′,4′_=9.6 Hz, H‐3′), 5.28‐5.22 (m, 1H, β^Myr^−C*H*), 5.22‐5.13 (m, 2H, β^Myr^−C*H*), 4.93‐4.89 (m, 2H, H‐1, H‐1’), 4.47‐4.37 (m, 2H, H‐3, H‐6b), 4.25 (dd, 1H, ^2^ *J*_6a,6b_=12.1 Hz, ^3^ *J*_5,6a_=6.0 Hz, H‐6a), 4.20‐4.13 (m, 2H, H‐4, H‐4’), 3.88‐3.75 (m, 2H, H‐6a′, H‐6b′), 3.70‐3.61 (m, 3H, H‐2, H‐5, H‐2’), 3.47 (ddd, 1H, ^3^ *J*_4′,5′_=9.7 Hz, ^3^ *J*_5′,6a′_=4.7 Hz, ^3^ *J*_5′,6b′_=2.0 Hz, H‐5′), 2.72–2.53 (m, 4H, α^Myr^−C*H*_2_), 2.44 (dd, 1H, ^2^ *J*=14.9 Hz, ^3^ *J*=7.4 Hz, α^Myr^−C*H*_2_), 2.37–2.24 (m, 7H, α^Myr^−C*H*_2_, α^Cap^−C*H*_2_, α^Lau^−C*H*_2_), 1.96 (s, 3H, C*H*_3_, NHAc), 1.67‐1.49 (m, 12H, β^Myr^−C*H*_2,_ γ^Myr^−C*H*_2_, β^Lau^−C*H*_2_, β^Cap^−C*H*_2,_), 1.36‐1.19 (m, 102H, C*H*_2_, Myr, Lau, Cap) 0.90‐0.84 (m, 18H, ω^Myr^−C*H*_3_, ω^Lau^−C*H*_3_, ω^Cap^−C*H*_3_); ^13^C NMR (151 MHz, MeOD / CDCl_3_ 3 : 2): δ 174.78, 174.74, 174.61 (C=O, Myr, Lau, Cap), 173.80 (C=O, NHAc), 171.96, 171.54, 170.97 (C=O, Myr), 98.51 (CH, m, C‐1, C‐1′), 78.33 (CH, m, C‐3), 76.33 (CH, d, ^3^ *J*_C,P_=4.3 Hz, C‐5′), 74.74 (CH, m, C‐4), 73.78‐73.68 (CH, m, C‐5, C‐3′), 73.09 (CH, d, ^2^ *J*_C,P_=5.2 Hz, C‐4′), 71.46, 71.37, 70.91 (CH, β^Myr^−CH), 64.07 (CH_2_, C‐6), 61.59 (CH, C‐6′), 55.87 (CH, C‐2), 55.05 (CH, C‐2′), 41.68, 39.68, 39.49 (CH_2_, α^Myr^−CH_2_), 35.28, 35.20, 35.13, 34.98, 34.91, 34.87 (CH_2_, Myr, Lau, Cap), 32.69, 32.67, 32.64 (CH_2_, Myr, Lau, Cap), 30.52‐29.90 (CH_2_, m, Myr, Lau, Cap), 25.97, 25.93, 25.87, 25.83, 25.79 (CH_2_, m, Myr, Lau, Cap), 23.39, 23.29, (CH_2_, m, Myr, Lau, Cap), 23.15 (CH_3_, NH*Ac*), 14.38 (CH_3_, ω^Myr^−CH_3_, ω^Lau^−CH_3,_ ω^Cap^−CH_3_); ^31^P NMR (243 MHz, MeOD / CDCl_3_ 3 : 2): δ 0.63, 0.28, −0.26; MALDI‐TOF MS: *m/z* calcd. for [M−H]^−^ C_92_H_172_N_2_O_28_P_3_ 1846.13, found 1846.11.

### Immunobiology

#### Sample preparation

Synthetic compounds (0.1 mg) were dissolved in DMSO (100 μL, TLR4 grade, Sigma) under vortex to provide 1 mg/ml solutions using glass vials. A 50 μL aliquot was placed in a 2–3 mL glass vial and reconstituted with 450 μL DMEM or RPMI cell medium supplemented with 10 % FCS (fetal calf serum) to provide 500 μL of a 100 μg/mL stock solution. The latter was used for further dilutions with cell medium (as indicated below for each particular experiment) to provide variably concentrated aqueous solutions of DLAMs. Glass vials were used for all dilutions. The stability and integrity of DLAMs in the aqueous buffers/cell medium (100 μg/mL) was confirmed using MALDI‐TOF spectroscopy.

#### Stimulation of transient human TLR4/MD‐2/CD14‐transfected HEK293 cells with ββ‐DLAMs

HEK293 cells were transfected for 24 h with plasmids coding for hTLR4 (kind gift of P. Nelson, USA), hMD2 (kind gift of K. Miyake, Tokyo, Japan), and hCD14 (kind gift of D. Golenbock, Worcester, USA), using Lipofectamine 2000 (Invitrogen) according to the manufacture's instruction. Solutions of *ββ*‐DLAMs were prepared as described above using DMEM cell medium. Transiently transfected HEK293 cells were stimulated with increasing concentrations of *ββ*‐DLAMs or *E. coli* O111:B4 LPS for 20 h. For inhibition experiments, cells were preincubated with *ββ*‐DLAMs (10 μg/mL) for 1 h before stimulation with *E. coli* O111:B4 LPS for 20 h. Recombinant human TNF‐α (kind gift of D. Männel, Germany) served as transfection‐independent control. Production of IL‐8 was measured by human IL‐8 CytoSet ELISA (Invitrogen) according to the manufacturer's instruction. Data were combined from n=4 independent experiments; error bars indicate standard error of the mean.

#### Activation of human mononuclear cells (MNC) by nanomolar concentrations of ββ‐DLAMs

Human peripheral blood mononuclear cells (MNC) from healthy human volunteers (prepared from heparinized blood by gradient centrifugation using Biocoll, Merck) were incubated at a concentration of 1×10^6^/mL in 96‐well tissue culture plates at a volume of 150 μL using RPMI‐1640 medium supplemented with 100 U/mL penicillin, 100 μg/mL streptomycin (both PAA Laboratories GmbH), and 10 % of heat‐inactivated FCS (Merck Millipore, Biochrom AG). Solutions of *ββ*‐DLAMs were prepared as described above using RPMI cell medium. Cells were stimulated with increasing concentrations of compounds or *E. coli* O111:B4 LPS. After a culture period of 20 h at 37 °C, culture supernatants were harvested and the IL‐6 and IL‐1β content was determined using an ELISA according to the manufacturers’ protocol (Invitrogen). For inhibition experiments, cells were preincubated with *ββ*‐DLAMs (10 μg/mL) for 1 hr before subsequent stimulation with *E. coli* O111:B4 LPS for 20 h. Data were combined from n=3 independent experiments; error bars indicate standard error of the mean.

#### Activation of human bronchial epithelial cells by ββ‐DLAMs

Calu‐3 cells (a human lung epithelial cell line; ATCC) were seeded in 96 well plates at 10E5 cells/well in 100 μL of complete medium (RPMI1640 (PAA), 1 % PS (PAA), 10 % FCS (Biochrom, Germany). On the next day, cells were washed once with complete medium and stimulated with increasing concentrations of *ββ*‐DLAMs or *E. coli* O111:B4 LPS. The cells were incubated for 20–24 h and the supernatants were analyzed for IL‐8 and IL‐6 by ELISA (Invitrogen). Data were combined from n=3 independent experiments, error bars indicate standard error of the mean.

#### Expression of TNF‐α in a bone marrow‐derived mouse wt macrophage cell line stimulated by ββ‐DLAMs

Immortalized C57BL/6 wt mouse macrophage cell lines were kindly provided by D.T. Golenbock^22^ (Worcester, USA) and propagated in RPMI medium (PAA, Austria) containing 10 % FCS, 20 mM HEPES buffer, 2 mM L‐Glutamin (PAA, Austria) and 20 μg/mL gentamicin (Sigma). Cells were stimulated with increasing concentrations of compounds or *E. coli* O111:B4 LPS for 20 h. For inhibition experiments, cells were preincubated with *ββ*‐DLAMs (1 μg/ml) for 1 h before subsequent stimulation with increasing amounts of *E. coli* O111:B4 LPS for 20 h. TNF‐α production was measured by mouse TNF‐α CytoSet ELISA (Invitrogen) according to the manufacturer's instruction. Data shown were combined from n=3 independent experiments, error bars indicate standard error of the mean.

#### Activation of human monocyte‐derived dendritic cells (moDCs)

Preparation of monocyte‐derived dendritic cells was performed by harvesting MNCs as described above and subsequent isolation of CD14^+^ monocytes by magnetic sorting (MojoSort, BioLegend). MoDCs were generated by addition of interleukin 4 and granulocyte‐macrophage colony stimulating factor (both 500 U/mL, ImmunoTools, Germany) to monocytes in 6‐well plates for 7 days as described previously, harvested, and resuspended in complete RPMI for stimulation experiments. MoDCs were grown and incubated in a humidified atmosphere of 5 % carbon dioxide at 37 °C. For the stimulation, compounds were added to moDCs (1x10^6^/mL) in the indicated concentrations and incubated for 20 h. Cytokine releases of stimulated moDCs were quantified after 20 h by using commercial ELISA Kits (Life Technologies GmbH, Darmstadt, Germany) specific for IL‐6, IL‐10 and IL‐12‐p70 in supernatants. Data were combined from n=3 independent experiments; error bars indicate standard error of the mean.

#### Ethics statement

Approval for these studies was obtained from the Institutional Ethics Committee at the University of Lübeck (Lübeck, Germany; Az. 12–202 A) according to the Declaration of Helsinki. All donors gave written informed consent.

#### Statistics

All statistics were calculated with Prism 9. Calculations of EC_50_ and R square were done by using nonlinear fit equation ([agonist] vs. response (three parameters); least squares fit).

## Conflict of interest

The authors declare no conflict of interest.

1

## Supporting information

As a service to our authors and readers, this journal provides supporting information supplied by the authors. Such materials are peer reviewed and may be re‐organized for online delivery, but are not copy‐edited or typeset. Technical support issues arising from supporting information (other than missing files) should be addressed to the authors.

Supporting Information

## Data Availability

The data that support the findings of this study are available in the supplementary material of this article.
